# Supplementary material for: A rare case of brominated small molecule acceptors for high-efficiency organic solar cells
Source: Nat Commun. 2023 Aug 5;14:4707. doi: 10.1038/s41467-023-40423-6 (PMC10404295; doi:10.1038/s41467-023-40423-6)
Supplement: Supplementary file 1 — Supplementary Information [file 41467_2023_40423_MOESM1_ESM.pdf]

Supplementary Information

A Rare Case of Brominated Small Molecule Acceptors for High-Efficiency Organic Solar Cells

Huazhe Liang[1,†], Xingqi Bi[1,†], Hongbin Chen[1], Tengfei He[1], Yi Lin[2], Yunxin Zhang[3], Kangqiao Ma[1], Wanying Feng[1], Zaifei Ma[2], Guankui Long[3], Chenxi Li[1], Bin Kan[3], Hongtao Zhang[1], Oleg A. Rakitin[4], Xiangjian Wan[1], Zhaoyang Yao[1,*], Yongsheng Chen[1,*]

[1]State Key Laboratory and Institute of Elemento-Organic Chemistry, The Centre of Nanoscale Science and Technology and Key Laboratory of Functional Polymer Materials, Renewable Energy Conversion and Storage Center (RECAST), College of Chemistry, Nankai University, Tianjin, 300071, China.

[2]State Key Laboratory for Modification of Chemical Fibers and Polymer Materials, Center for Advanced Low-dimension Materials, College of Materials Science and Engineering, Donghua University, Shanghai, 201620, China.

[3]School of Materials Science and Engineering, National Institute for Advanced Materials, Renewable Energy Conversion and Storage Center (RECAST), Nankai University, Tianjin, 300350, China.

[4]N. D. Zelinsky Institute of Organic Chemistry, Russian Academy of Sciences, 119991 Moscow, Russia.

[†]These authors contributed equally: Huazhe Liang, Xingqi Bi.
Corresponding E-mails: yschen99@nankai.edu.cn (Y.C.); zyao@nankai.edu.cn.

1. Supplementary Methods

The overall synthesis of compound 2,2'-((2Z,2'Z)-((15,16-bis(2-hexyldecyl)-3,12-diundecyl-15,16-dihydrothieno[2'',3'':4',5']thieno[2',3':4,5]pyrrolo[3,2-a]thieno[2'',3'':4',5']thieno[2',3':4,5]pyrrolo[2,3-c]phenazine-2,13-diyl)bis(methaneylylidene))bis(5,6-difluoro-3-oxo-2,3-dihydro-1H-indene-2,1-diylidene))dimalononitrile (CH20), 2,2'-((2Z,2'Z)-((7-bromo-15,16-bis(2-hexyldecyl)-3,12-diundecyl-15,16-dihydrothieno[2'',3'':4',5']thieno[2',3':4,5]pyrrolo[3,2-a]thieno[2'',3'':4',5']thieno[2',3':4,5]pyrrolo[2,3-c]phenazine-2,13-diyl)bis(methaneylylidene))bis(5,6-difluoro-3-oxo-2,3-dihydro-1H-indene-2,1-diylidene))dimalononitrile (CH21) and 2,2'-((2Z,2'Z)-((7,8-dibromo-15,16-bis(2-hexyldecyl)-3,12-diundecyl-15,16-dihydrothieno[2'',3'':4',5']thieno[2',3':4,5]pyrrolo[3,2-a]thieno[2'',3'':4',5']thieno[2',3':4,5]pyrrolo[2,3-c]phenazine-2,13-diyl)bis(methaneylylidene))bis(5,6-difluoro-3-oxo-2,3-dihydro-1H-indene-2,1-diylidene))dimalononitrile (CH22).

As shown in Supplementary Fig. 1, the reagents and conditions used were as follows: (I) $LiAlH_4$, THF, reflux; (II) DDQ, $CHCl_3$, then benzene-1,2-diamine, 4-bromobenzene-1,2-diamineor 4,5-dibromobenzene-1,2-diamine, 0 °C to room temperature; (III) $POCl_3$, DMF, $ClCH_2CH_2Cl$, reflux; (IV) 2-(6,7-difluoro-3-oxo-2,3-dihydro-1H-cyclopenta[b] naphthalen-1-ylidene) malononitrile or 2-(5,6-dichloro-3-oxo-2,3-dihydro-1H-inden-1-ylidene) malononitrile, pyridine, $CHCl_3$, reflux.

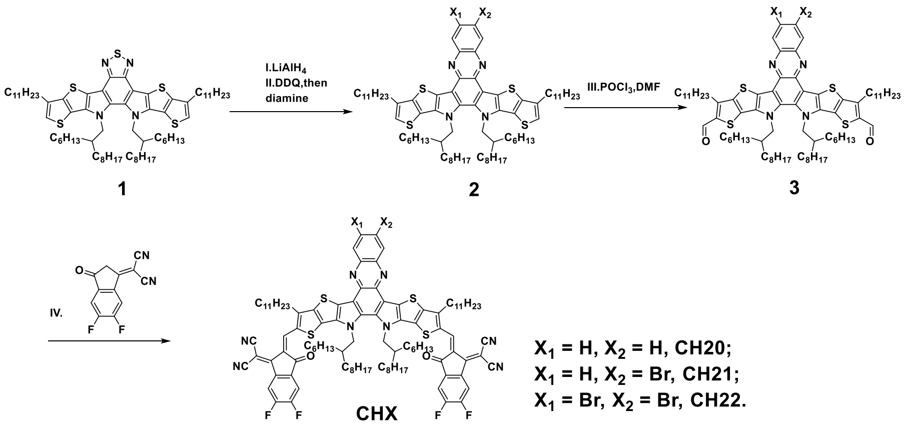

Supplementary Fig. 1 The overall synthetic route to CH20, CH21 and CH22.

Synthesis of 15,16-bis(2-hexyldecyl)-3,12-diundecyl-15,16-dihydrothieno[2'',3'':4',5']thieno[2',3':4,5]pyrrolo[3,2-a]thieno[2'',3'':4',5']thieno[2',3':4,5]pyrrolo[2,3-c]phenazine (2-0), 7-bromo-15,16-bis(2-hexyldecyl)-3,12-diundecyl-15,16-dihydrothieno[2'',3'':4',5']thieno[2',3':4,5]pyrrolo[3,2-a]thieno[2'',3'':4',5']thieno[2',3':4,5]pyrrolo[2,3-c]phenazine (2-1) and 7,8-dibromo-15,16-bis(2-hexyldecyl)-3,12-diundecyl-15,16-dihydrothieno[2'',3'':4',5']thieno[2',3':4,5]pyrrolo[3,2-a]thieno[2'',3'':4',5']thieno[2',3':4,5]pyrrolo[2,3-c]phenazine (2-2).

As shown in Supplementary Fig. 2, under the protection of argon, $LiAlH_4$ (119 mg, 3.14 mmol, 5.0 eq.) was added to a solution of 12,13-bis(2-hexyldecyl)-3,9-diundecyl-12,13-dihydro-[1,2,5]thiadiazolo[3,4-e]thieno[2",3":4',5'] thieno[2',3':4,5]pyrrolo[3,2-g]thieno[2',3':4,5]thieno[3,2-b]indole (**1**) (750 mg, 0.63 mmol, 1.0 eq.) in tetrahydrofuran (THF, 50 mL). The resulting mixture was stirred and heated to reflux for 12 h. After being cooled to 0 °C, water (30 mL) is slowly dropped into the reaction and extracted with dichloromethane. The organic layer was dried over anhydrous Na_2SO_4 for 1 h. After removal of solvent, the crude product was dissolved in chloroform (45 mL), then 3-dichloro-5, 6-dicyano-1, 4-benzoquinone (DDQ, 285 mg, 1.26 mmol, 2.0 eq.) and benzene-1,2-diamine, (339 mg, 3.14 mmol, 5.0 eq.) were added to the solution in turn. The reaction was stirred at room temperature for 6 h, and the solvent was removed under vacuum. Finally, the residue was purified by column chromatography to give compound **2-0** as a red solid. (625 mg, 79%). Compound **2-1** is obtained by a similar method with a yield of 75% as a red solid. Compound **2-2** is obtained by a similar method with a yield of 78% as a red solid.

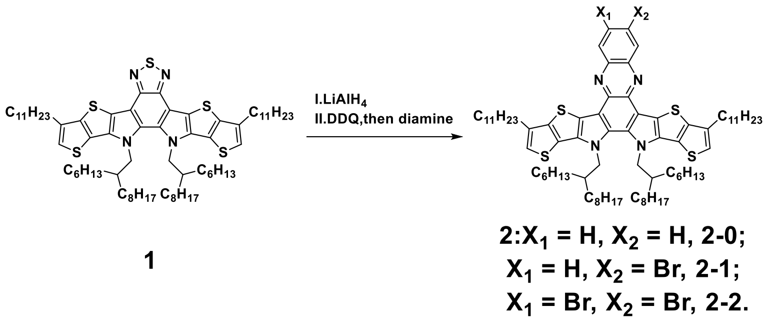

Supplementary Fig. 2 Synthesis of compound **2**.

Data for compound **2-0**: ^{1}H NMR (400 MHz, Chloroform-*d*) δ 8.50 (dd, *J* = 6.5, 3.4 Hz, 2H), 7.86 (dd, *J* = 6.5, 3.4 Hz, 2H), 7.06 (s, 2H), 4.72 (d, *J* = 7.8 Hz, 4H), 2.92 (t, *J* = 7.7 Hz, 4H), 2.23 (h, *J* = 6.4 Hz, 2H), 1.94 (q, *J* = 7.6 Hz, 4H), 1.17 (s, 86H), 0.83 (s, 6H), 0.71 (t, *J* = 7.0 Hz, 6H). ^{13}C NMR (101 MHz, $CDCl_3$) δ 143.04, 141.46, 138.44, 136.93, 136.89, 131.58, 129.47, 129.40, 128.26, 128.09, 123.54, 123.48, 118.83, 117.14, 55.03, 38.76, 38.72, 32.00, 31.84, 31.69, 30.51, 30.42, 29.75, 29.62, 29.57, 29.45, 29.20, 28.99, 25.57, 22.77, 22.64, 22.58, 22.53, 14.23, 14.17, 14.13, 14.02.

Data for compound **2-1**: ^{1}H NMR (400 MHz, Chloroform-*d*) δ 8.68 (d, *J* = 2.1 Hz, 1H), 8.34 (d, *J* = 9.0 Hz, 1H), 7.90 (dd, *J* = 9.0, 2.2 Hz, 1H), 7.05 (s, 2H), 4.69 (d, *J* = 7.8 Hz, 4H), 2.90 (t, *J* = 7.7 Hz, 4H), 2.18 (h, *J* = 6.5 Hz, 2H), 1.92 (q, *J* = 7.6 Hz, 4H), 1.16 (d, *J* = 124.3 Hz, 86H), 0.81 (d, *J* = 7.3 Hz, 6H), 0.69 (t, *J* = 7.0 Hz, 6H). ^{13}C NMR (101 MHz, $CDCl_3$) δ 143.13, 141.73, 139.98, 138.68, 138.47, 136.94, 131.83, 131.64, 131.43, 130.70, 130.61, 123.49, 123.46, 123.37, 121.95, 119.02, 116.96, 116.86, 55.07, 38.73, 31.97, 31.82, 31.65, 30.40, 29.76, 29.71, 29.62, 29.54, 29.45, 29.42, 29.39, 29.18, 28.96, 25.54, 22.75, 22.62, 22.50, 14.20, 14.15, 14.11, 13.99.

Data for compound **2-2**: ^{1}H NMR (400 MHz, Chloroform-*d*) δ 8.75 (s, 2H), 7.05 (s, 2H), 4.70 (d, *J* = 7.9 Hz, 4H), 2.88 (t, *J* = 7.7 Hz, 4H), 2.20 (h, *J* = 6.6 Hz, 2H), 1.92 (p, *J* = 7.5 Hz, 4H), 1.43 – 0.90 (m, 86H), 0.81 (s, 6H), 0.69 (t, *J* = 7.0 Hz, 6H). ^{13}C NMR (101 MHz, $CDCl_3$) δ 143.17, 138.90, 136.98, 136.90, 133.06, 131.86, 124.01, 123.48, 123.41, 119.11, 116.77, 55.15, 38.76, 31.97, 31.82, 31.64, 29.62, 29.55, 29.46, 29.41, 29.18, 28.95, 25.56, 22.75, 22.62, 22.50, 14.14, 13.99.

Synthesis of 15,16-bis(2-hexyldecyl)-3,12-diundecyl-15,16-dihydrothieno[2'',3'':4',5']thieno[2',3':4,5]pyrrolo[3,2-a]thieno[2'',3'':4',5']thieno[2',3':4,5]pyrrolo[2,3-c]phenazine-2,13-dicarbaldehyde (3-0), 7-bromo-15,16-bis(2-hexyldecyl)-3,12-diundecyl-15,16-dihydrothieno[2'',3'':4',5']thieno[2',3':4,5]pyrrolo[3,2-a]thieno[2'',3'':4',5']thieno[2',3':4,5]pyrrolo[2,3-c]phenazine-2,13-dicarbaldehyde (3-1) and 7,8-dibromo-15,16-bis(2-hexyldecyl)-3,12-diundecyl-15,16-dihydrothieno[2'',3'':4',5']thieno[2',3':4,5]pyrrolo[3,2-a]thieno[2'',3'':4',5']thieno[2',3':4,5]pyrrolo[2,3-c]phenazine-2,13-dicarbaldehyde (3-2).

As shown in Supplementary Fig. 3, under the protection of argon, phosphorus oxychloride (0.3 mL) was added to a solution of compound **2** (550 mg) and N, N-Dimethylformamide (DMF, 0.3 mL) in 1, 2-dichloroethane ($ClCH_2CH_2Cl$, 50 mL). The resulting mixture was stirred and heated to reflux for 12 h, then cooled to 0 °C. The resulting mixture was slowly added to a saturated solution of sodium acetate (40 mL), then stirred at room temperature for 2 h. The resulting mixture was extracted with dichloromethane and the organic layer was dried over anhydrous Na_2SO_4 for 1 h. After removal of solvent, the crude product was then purified by column chromatography on silica gel with hexanes/dichloromethane (v/v=1:2) as eluent to afford compound **3-0** as a red solid (498 mg, 82%). Compound **3-1** is obtained by a similar method with a yield of 85% as a red solid. Compound **3-2** is obtained by a similar method with a yield of 86% as a red solid.

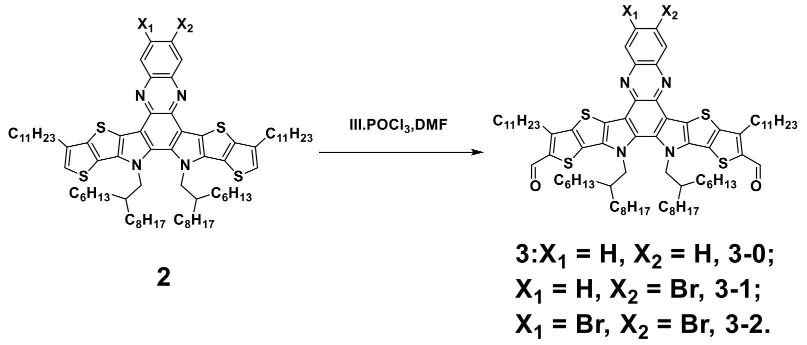

Supplementary Fig. 3 Synthesis of compound **3**.

Data for compound **3-0**: ^{1}H NMR (400 MHz, Chloroform-*d*) δ 10.18 (s, 2H), 8.46 (dd, *J* = 6.7, 3.5 Hz, 2H), 7.88 (dd, *J* = 6.7, 3.5 Hz, 2H), 4.73 (d, *J* = 7.9 Hz, 4H), 3.26 (t, *J* = 7.7 Hz, 4H), 2.17 (m, 2H), 1.99 (m, 4H), 1.51 (m, 4H), 1.29 (m, 82H), 0.78 (m, *J* = 7.3 Hz, 6H), 0.69 (t, *J* = 6.9 Hz, 6H). ^{13}C NMR (101 MHz, $CDCl_3$) δ 181.68, 146.94, 144.16, 141.73, 138.27, 136.71, 136.65, 132.77, 129.61, 129.40, 128.92, 128.22, 118.04, 55.29, 38.96, 31.93, 31.76, 31.58, 30.55, 30.48, 30.38, 29.70, 29.65, 29.57, 29.43, 29.37, 29.31, 29.15, 28.19, 26.93, 25.52, 22.70, 22.57, 22.48, 14.13, 14.08, 13.95.

Data for compound **3-1**: ^{1}H NMR (400 MHz, Chloroform-*d*) δ 10.18 (s, 2H), 8.56 (d, *J* = 2.2 Hz, 1H), 8.25 (d, *J* = 9.0 Hz, 1H), 7.88 (dd, *J* = 9.0, 2.2 Hz, 1H), 4.73 (d, *J* = 8.0 Hz, 4H), 3.24 (q, *J* = 8.6, 8.0 Hz, 4H), 2.19 (dt, *J* = 12.5, 6.7 Hz, 2H), 1.97 (q, *J* = 7.7 Hz, 4H), 1.51 (s, 4H), 1.35 – 0.88 (m, 82H), 0.79 (m, 6H), 0.69 (t, *J* = 6.9 Hz, 6H). ^{13}C NMR (101 MHz, $CDCl_3$) δ 181.71, 146.90, 146.87, 144.21, 141.90, 140.19, 138.52, 138.27, 136.83, 136.79, 136.72, 136.67, 132.93, 132.77, 132.28, 131.29, 130.51, 129.53, 129.46, 128.19, 128.06, 122.84, 117.84, 117.74, 55.39, 39.02, 31.92, 31.76, 31.57, 30.55, 30.51, 30.43, 29.76, 29.72, 29.69, 29.64, 29.58, 29.44, 29.36, 29.31, 29.15, 28.20, 25.56, 22.70, 22.57, 22.47, 14.13, 14.07, 13.95.

Data for compound **3-2**: ^{1}H NMR (400 MHz, Chloroform-*d*) δ 10.18 (s, 2H), 8.67 (s, 2H), 4.72 (d, *J* = 8.0 Hz, 4H), 3.23 (t, *J* = 7.8 Hz, 4H), 2.22 – 2.14 (m, 2H), 1.96 (t, *J* = 7.7 Hz, 4H), 1.51 (d, *J* = 7.6 Hz, 4H), 1.30 (s, 82H), 0.78 (m,, 6H), 0.69 (m,, 6H). ^{13}C NMR (101 MHz, $CDCl_3$) δ 181.74, 146.86, 144.31, 140.47, 138.66, 136.91, 136.71, 132.90, 129.43, 128.09, 125.05, 117.55, 55.45, 39.05, 31.91, 31.75, 31.56, 30.53, 30.45, 29.76, 29.74, 29.68, 29.64, 29.60, 29.44, 29.35, 29.32, 29.15, 28.20, 25.60, 25.56, 22.69, 22.56, 22.46, 14.13, 14.06, 13.95.

Synthesis of compound CH20, CH21 and CH22.

As shown in Supplementary Fig. 4, under argon protection, compound **3** (221 mg, 0.07 mmol, 1.0 eq.), 2-(5,6-difluoro-3-oxo-2,3-dihydro-1H-inden-1-ylidene)malononi trile (49 mg, 0.21 mmol, 3 eq.) and 30 mL dry chloroform were added to 100 mL two-necked round bottom flask. Then 0.5 mL pyridine was dropped into the mixture. The r

eaction mixture was stirred at 70 °C for 12 h. After cooling to room temperature, the r eaction mixture was precipitated in 70 mL methanol. The precipitate was purified by c olumn chromatography on silica gel with petroleum ether/ chloroform (v/v=4/5) as el uent to give black compound CH20 (182 mg, 79%); Compound CH21 was obtained b y a similar method with a yield of 76% as a black solid. Compound CH22 was obtaine d by a similar method with a yield of 82% as a black solid.

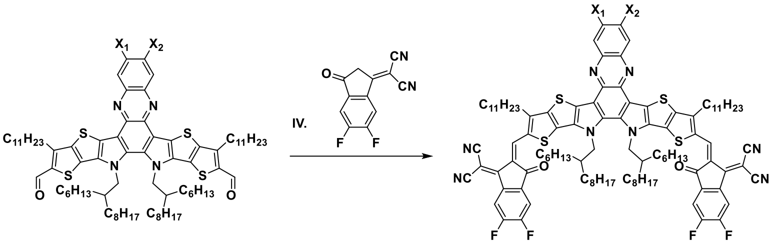

X_1 = H, X_2 = H, CH20;
X_1 = H, X_2 = Br, CH21;
X_1 = Br, X_2 = Br, CH22.

Supplementary Fig. 4 Synthesis of compound CH20, CH21 and CH22.

Data for compound CH20: ^{1}H NMR (400 MHz, Chloroform-*d*) δ 9.12 (s, 2H), 8.48 (dd, *J* = 9.9, 6.5 Hz, 2H), 8.38 (dd, *J* = 6.5, 3.4 Hz, 2H), 7.90 (dd, *J* = 6.6, 3.4 Hz, 2H), 7.69 (t, *J* = 7.5 Hz, 2H), 4.87 (d, *J* = 8.0 Hz, 4H), 3.28 (t, *J* = 7.9 Hz, 4H), 2.36 – 2.25 (m, 2H), 1.91 (p, *J* = 7.8 Hz, 4H), 1.58 (d, *J* = 4.2 Hz, 4H), 1.42 (t, *J* = 7.3 Hz, 4H), 1.29 -1.04 (m, 72H), 0.87 (d, *J* = 7.0 Hz, 6H), 0.77 (t, *J* = 7.1 Hz, 6H), 0.71 (t, *J* = 6.6 Hz, 6H). ^{13}C NMR (101 MHz, $CDCl_3$) δ 186.06, 158.67, 155.62, 155.49, 154.10, 153.04, 152.90, 146.36, 141.86, 138.25, 137.70, 136.62, 136.54, 136.21, 135.09, 134.47, 134.40, 133.91, 133.17, 131.83, 129.55, 129.38, 119.42, 119.39, 115.04, 114.96, 114.75, 114.61, 112.37, 112.19, 68.12, 55.74, 39.33, 31.92, 31.85, 31.66, 31.42, 30.67, 30.58, 29.88, 29.73, 29.65, 29.58, 29.55, 29.42, 29.37, 29.22, 25.72, 22.69, 22.59, 22.52, 14.11, 14.06, 14.03.

Data for compound CH21: ^{1}H NMR (400 MHz, Chloroform-*d*) δ 9.08 (d, *J* = 13.4 Hz, 2H), 8.47 (q, *J* = 7.4 Hz, 2H), 8.38 (s, 1H), 8.09 (d, *J* = 9.0 Hz, 1H), 7.83 (d, *J* = 9.1 Hz, 1H), 7.72 (t, *J* = 7.5 Hz, 2H), 4.86 (d, *J* = 8.0 Hz, 4H), 3.22 (q, *J* = 8.9 Hz,

4H), 2.29 (s, 2H), 1.87 (q, J = 8.1 Hz, 4H), 1.52 (s, 4H), 1.38 (s, 4H), 1.03 (s, 72H), 0.87 – 0.83 (m, 6H), 0.72 (dt, J = 19.5, 6.9 Hz, 12H). ^{13}C NMR (101 MHz, $CDCl_3$) δ 186.13, 158.56, 155.70, 155.57, 154.01, 153.12, 152.96, 146.38, 141.90, 140.25, 138.43, 138.16, 137.74, 137.70, 136.60, 136.08, 136.04, 135.07, 134.48, 133.98, 133.78, 133.20, 133.17, 131.73, 131.59, 123.56, 119.55, 119.12, 118.99, 115.00, 114.97, 114.58, 112.42, 68.28, 39.45, 31.94, 31.87, 31.69, 31.47, 30.70, 29.94, 29.75, 29.68, 29.61, 29.46, 29.39, 29.26, 25.82, 22.71, 22.66, 22.62, 22.54, 14.13, 14.07.

Data for compound CH22: ^{1}H NMR (400 MHz, Chloroform-*d*) δ 9.12 (s, 2H), 8.55 (dd, J = 9.9, 6.4 Hz, 2H), 8.41 (s, 2H), 7.76 (t, J = 7.5 Hz, 2H), 4.90 (d, J = 7.9 Hz, 4H), 3.22 (s, 4H), 2.34 (s, 2H), 1.87 (q, J = 8.0, 7.5 Hz, 4H), 1.55 (d, J = 7.1 Hz, 4H), 1.39 (s, 4H), 1.37 – 1.04 (m, 72H), 0.90 – 0.85 (m, 6H), 0.74 (dt, J = 13.3, 6.2 Hz, 12H). ^{13}C NMR (101 MHz, $CDCl_3$) δ 186.13, 158.60, 155.77, 155.64, 153.87, 146.47, 140.29, 138.32, 137.65, 136.60, 135.88, 135.09, 134.46, 133.72, 133.20, 132.59, 131.49, 125.75, 119.71, 118.67, 114.95, 114.51, 112.56, 112.37, 68.41, 55.98, 39.60, 31.93, 31.85, 31.71, 31.42, 30.78, 30.04, 30.01, 29.94, 29.73, 29.69, 29.67, 29.63, 29.61, 29.49, 29.37, 29.24, 25.98, 25.91, 22.69, 22.59, 22.53, 14.12, 14.03.

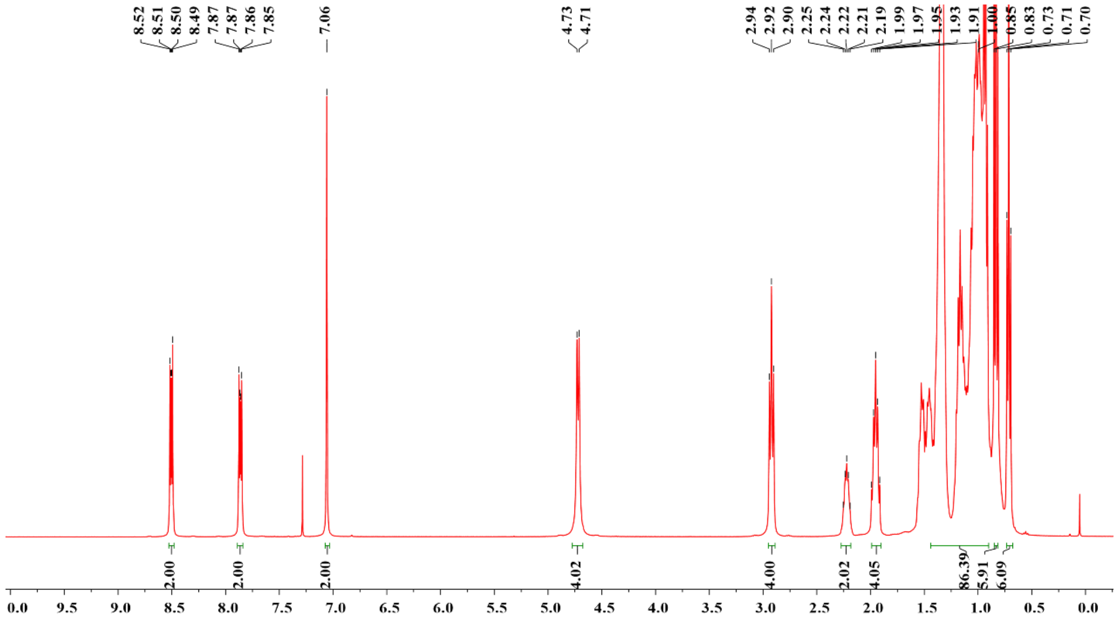

Supplementary Fig. 5 ^{1}H NMR spectrum of compound 2-0 at 300K in $CDCl_3$.

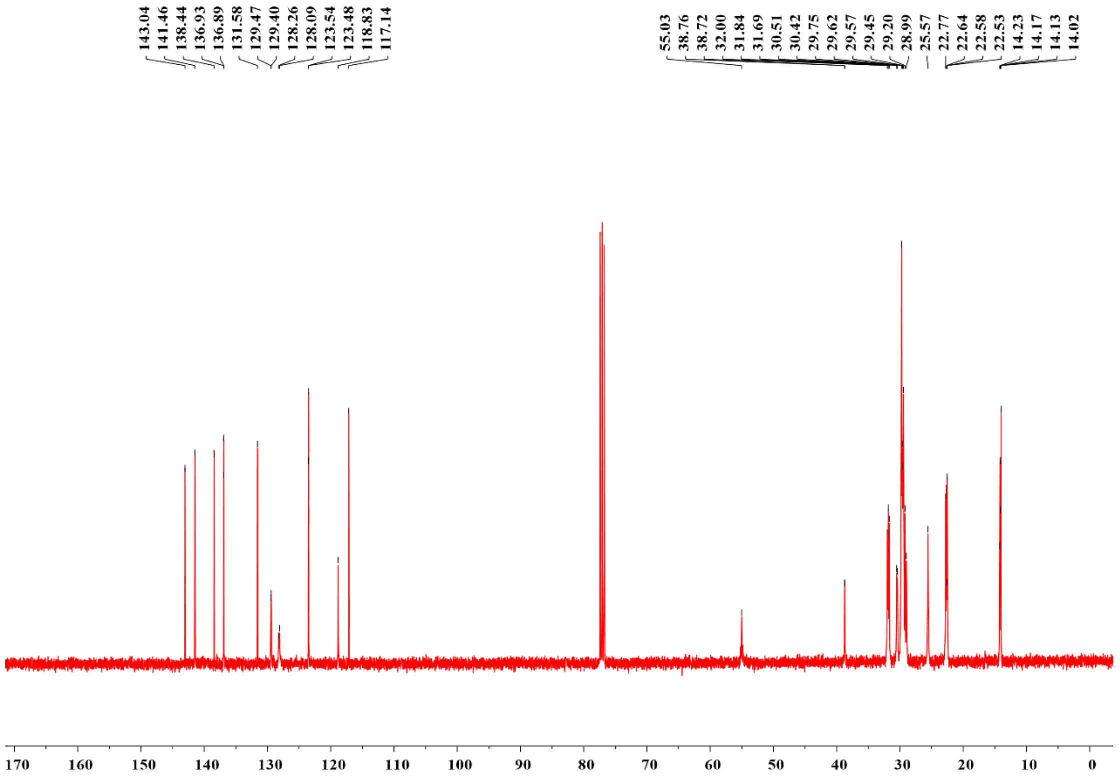

Supplementary Fig. 6 ^{13}C NMR spectrum of compound 2-0 at 300K in $CDCl_3$.

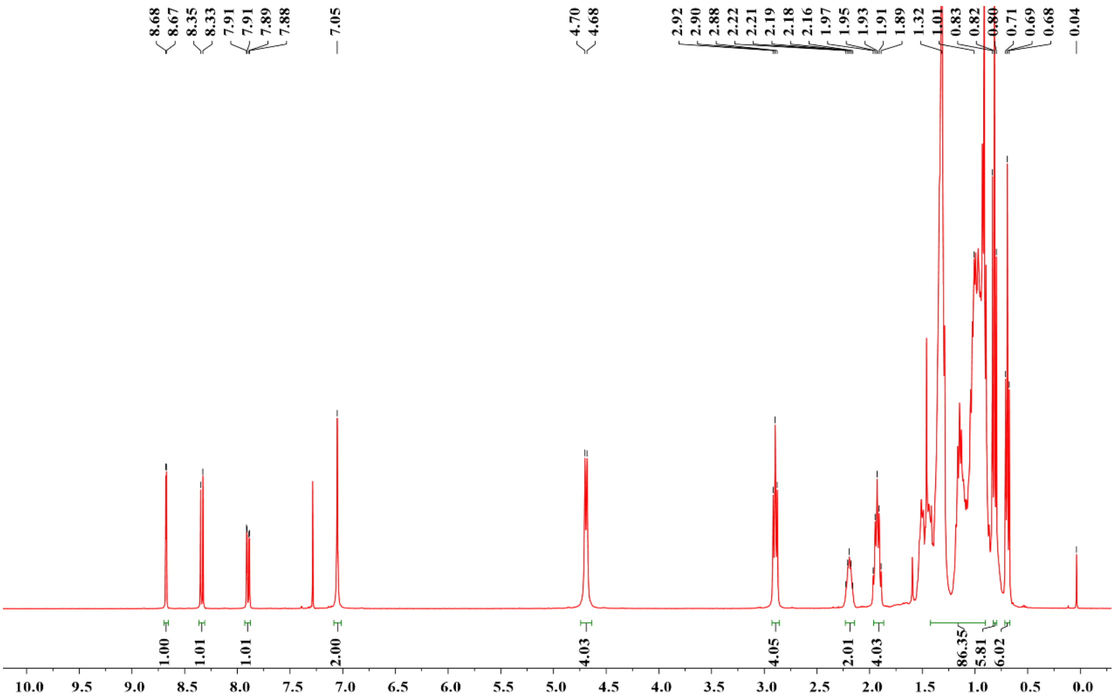

Supplementary Fig. 7 ^{1}H NMR spectrum of compound 2-1 at 300K in $CDCl_3$.

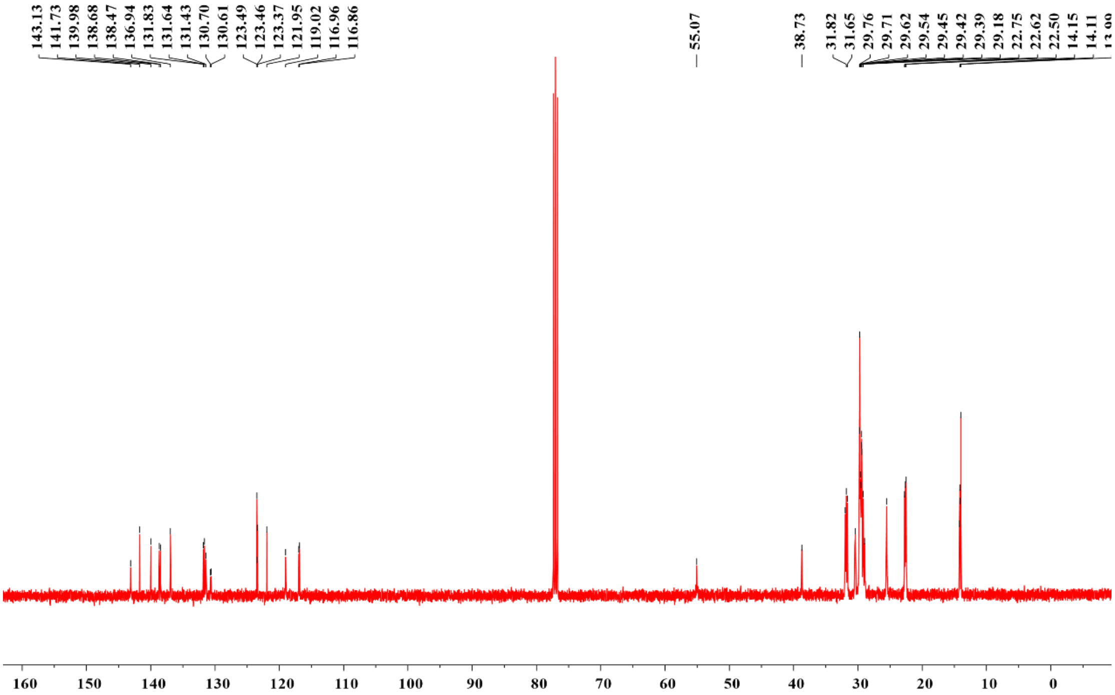

Supplementary Fig. 8 ^{13}C NMR spectrum of compound 2-1 at 300K in $CDCl_3$.

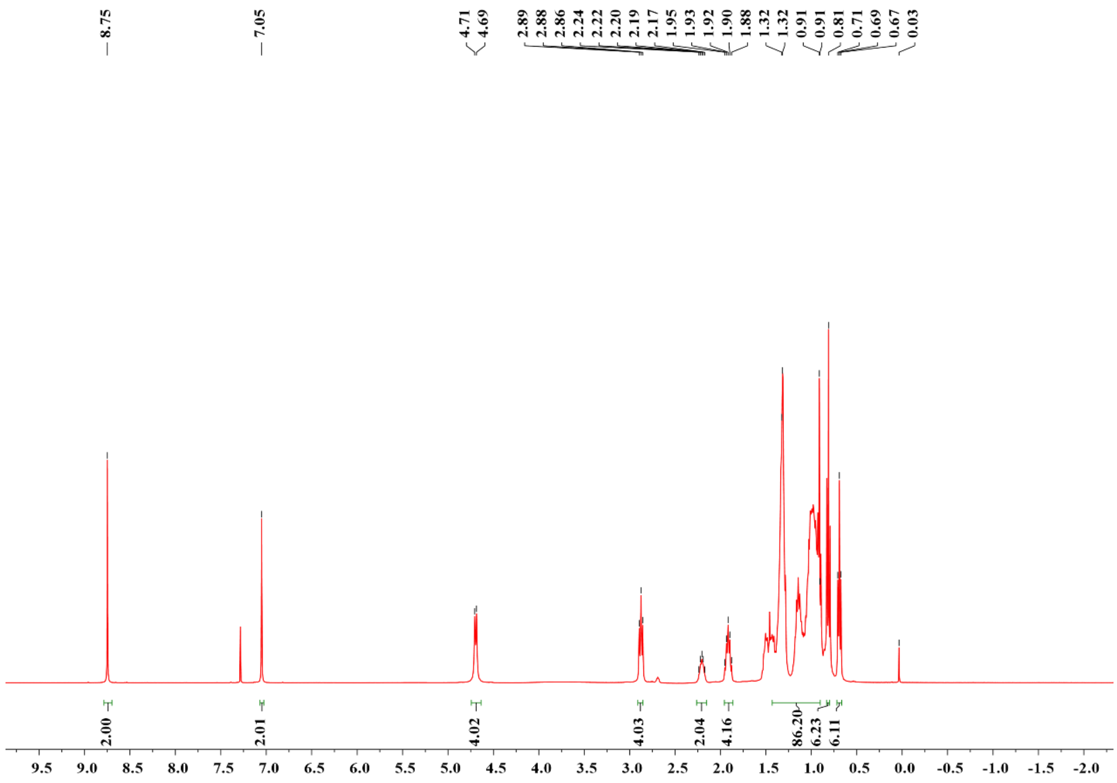

Supplementary Fig. 9 ^{1}H NMR spectrum of compound 2-2 at 300K in $CDCl_3$.

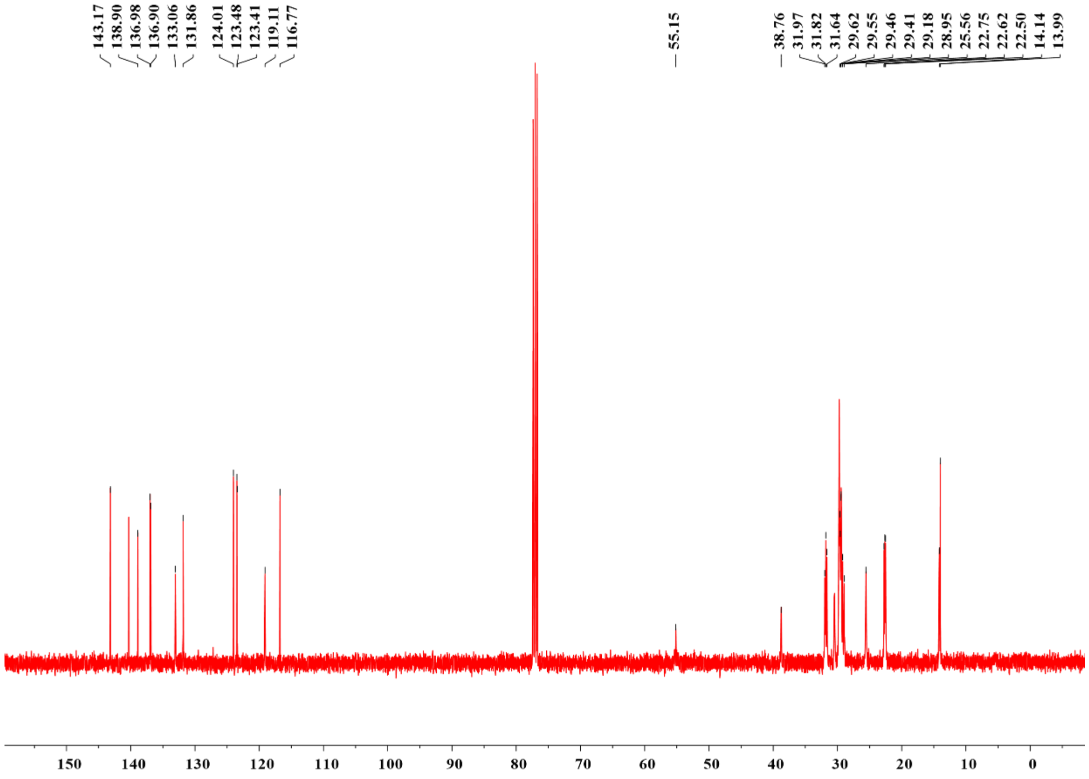

Supplementary Fig. 10 ^{13}C NMR spectrum of compound 2-2 at 300K in $CDCl_3$.

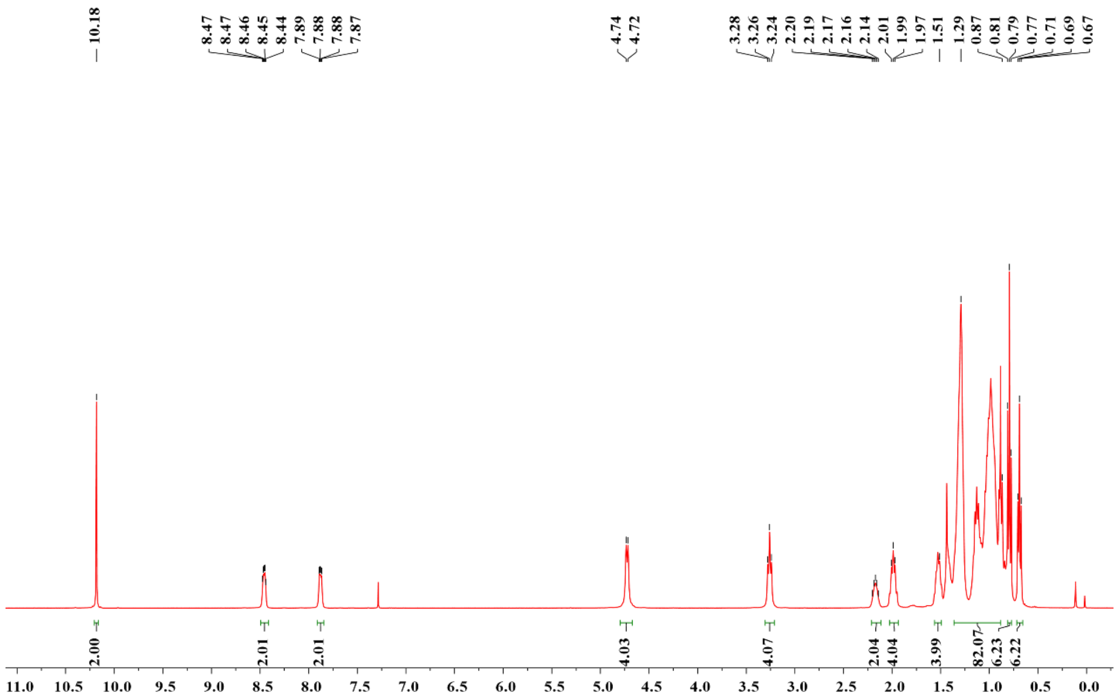

Supplementary Fig. 11 ^{1}H NMR spectrum of compound 3-0 at 300K in $CDCl_3$.

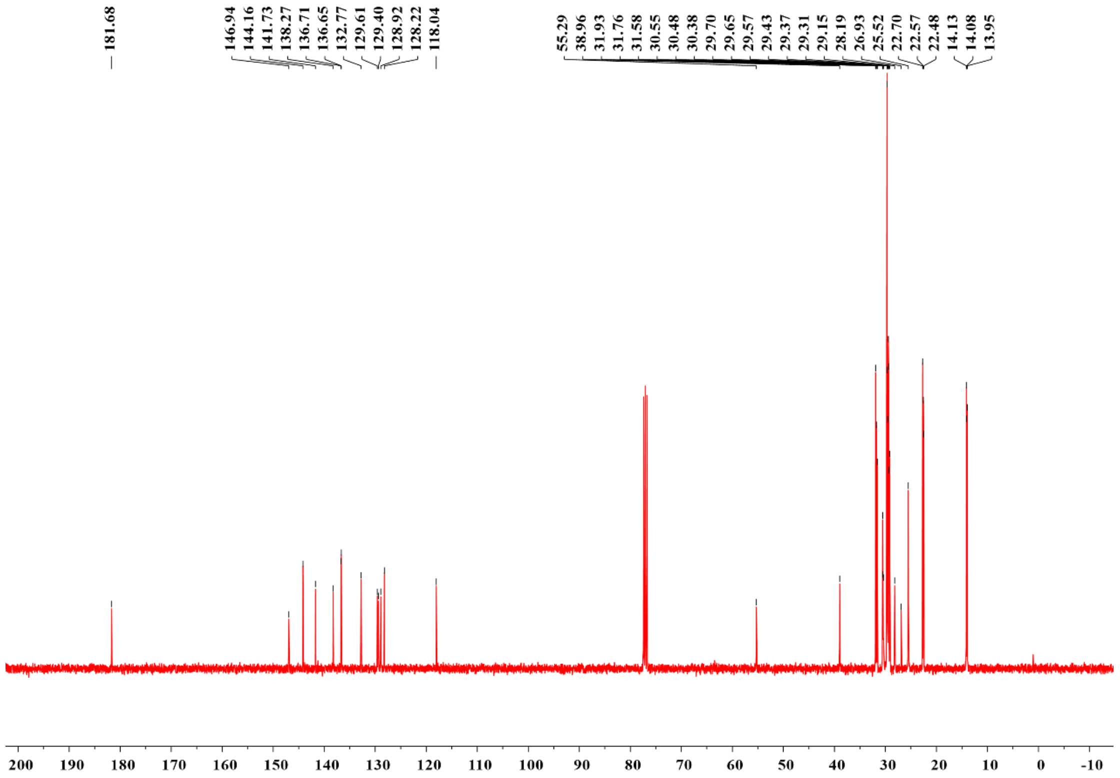

Supplementary Fig. 12 ^{13}C NMR spectrum of compound 3-0 at 300K in $CDCl_3$.

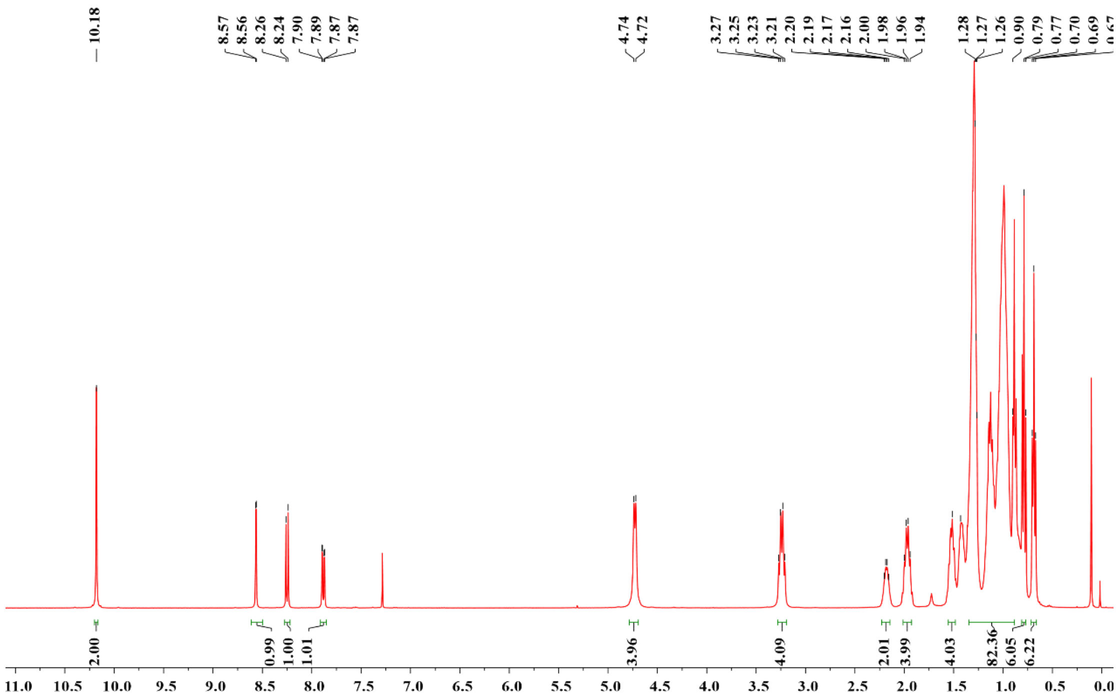

Supplementary Fig. 13 ^{1}H NMR spectrum of compound 3-1 at 300K in $CDCl_3$.

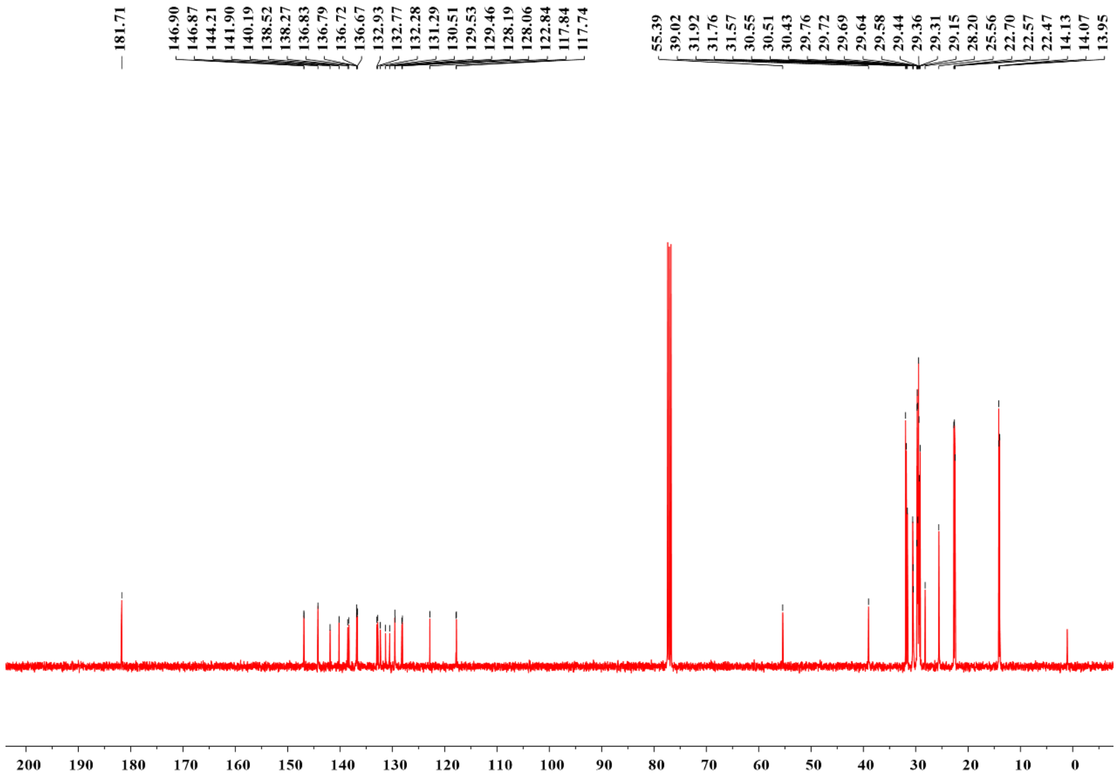

Supplementary Fig. 14 ^{13}C NMR spectrum of compound 3-1 at 300K in $CDCl_3$.

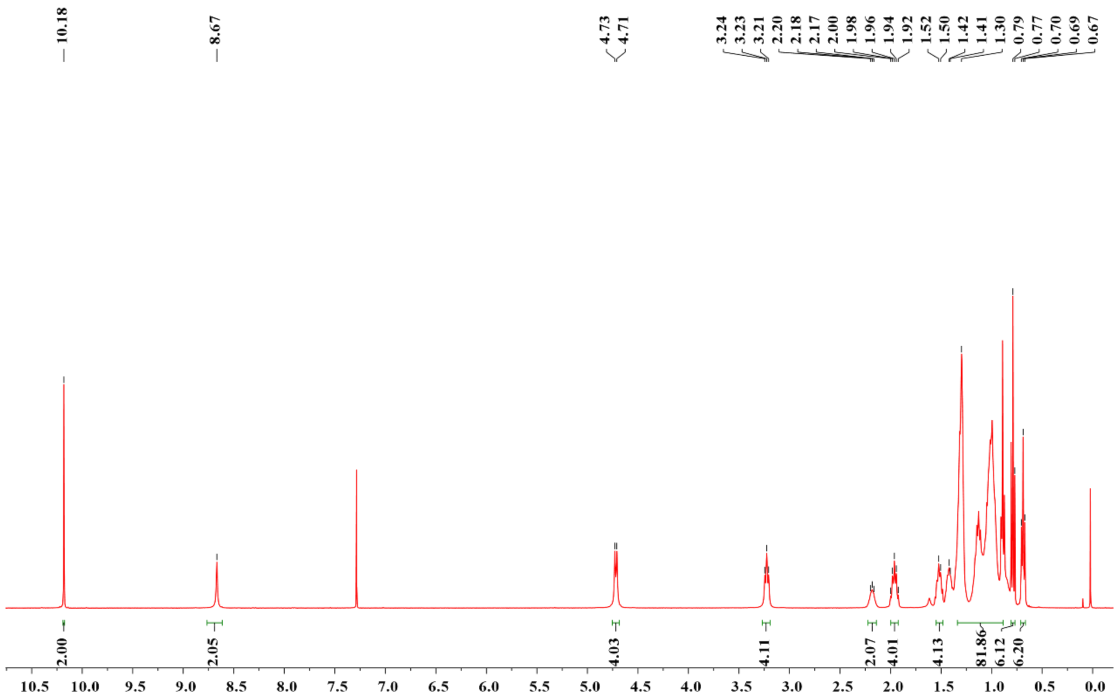

Supplementary Fig. 15 ^{1}H NMR spectrum of compound 3-2 at 300K in $CDCl_3$.

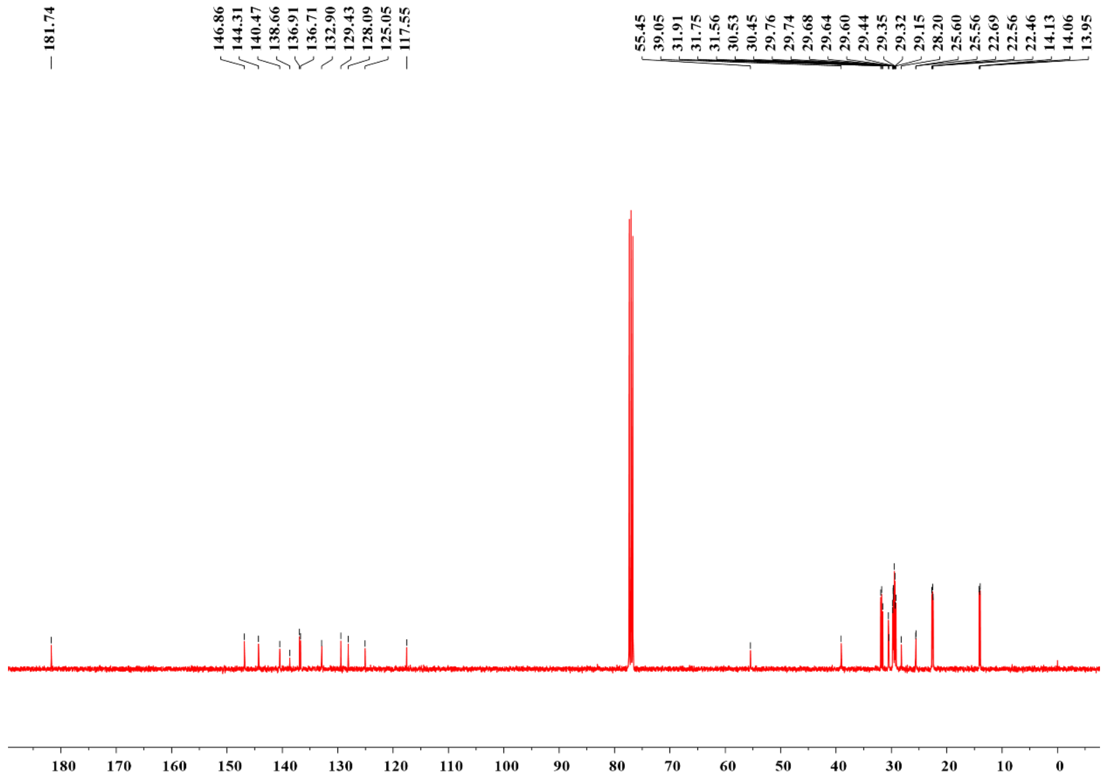

Supplementary Fig. 16 ^{13}C NMR spectrum of compound 3-2 at 300K in $CDCl_3$.

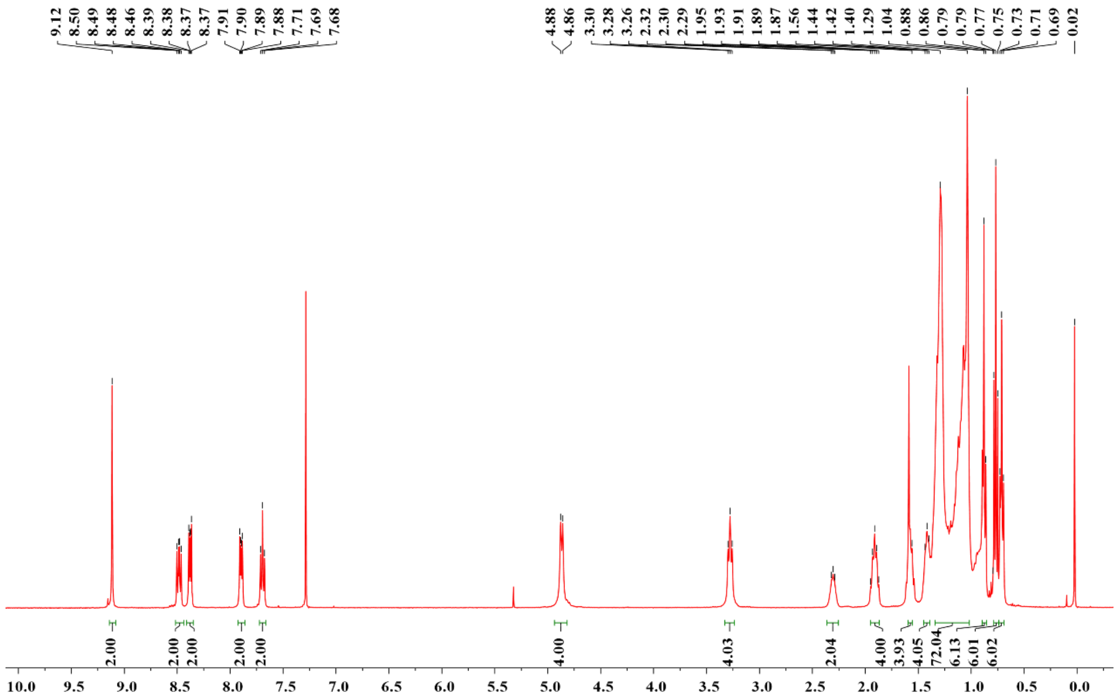

Supplementary Fig. 17 ^{1}H NMR spectrum of compound CH20 at 300K in $CDCl_3$.

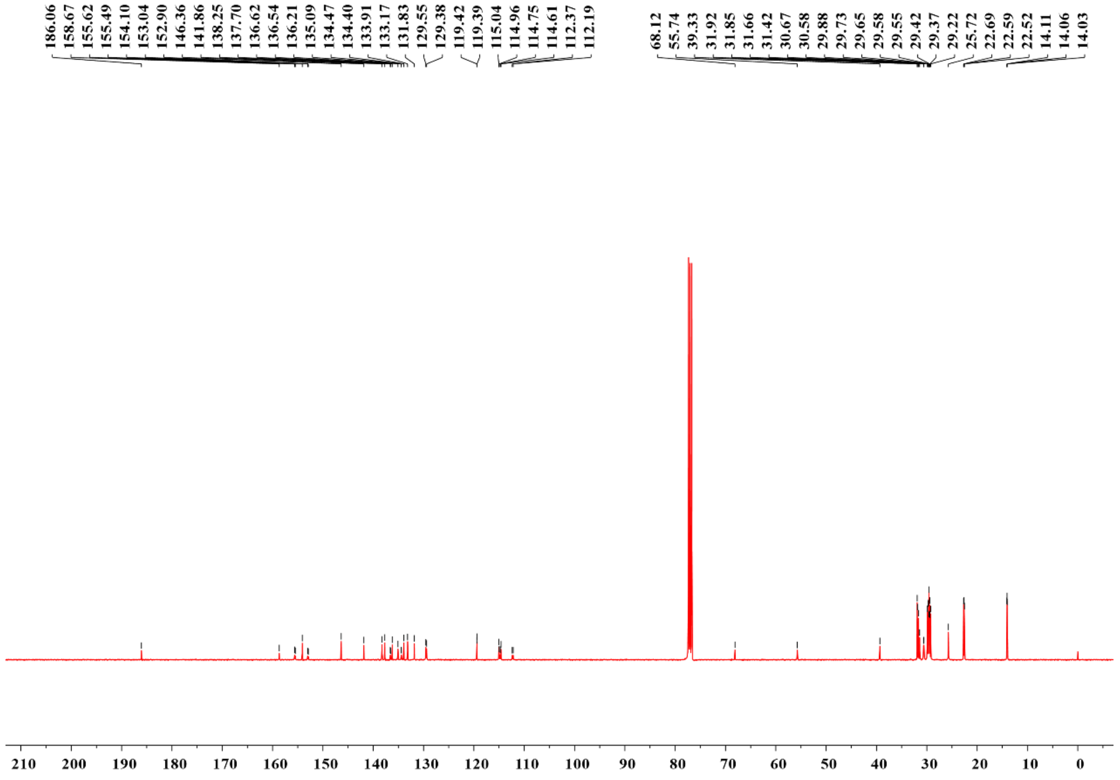

Supplementary Fig. 18 ^{13}C NMR spectrum of compound CH20 at 300K in $CDCl_3$.

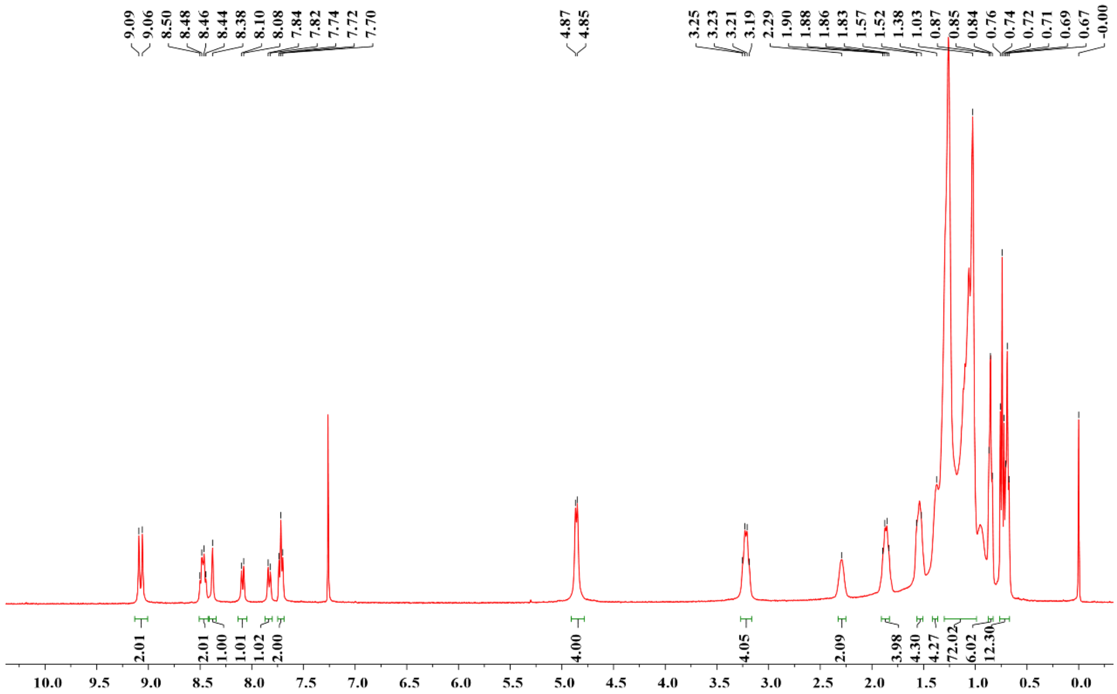

Supplementary Fig. 19 ^{1}H NMR spectrum of compound CH21 at 300K in $CDCl_3$.

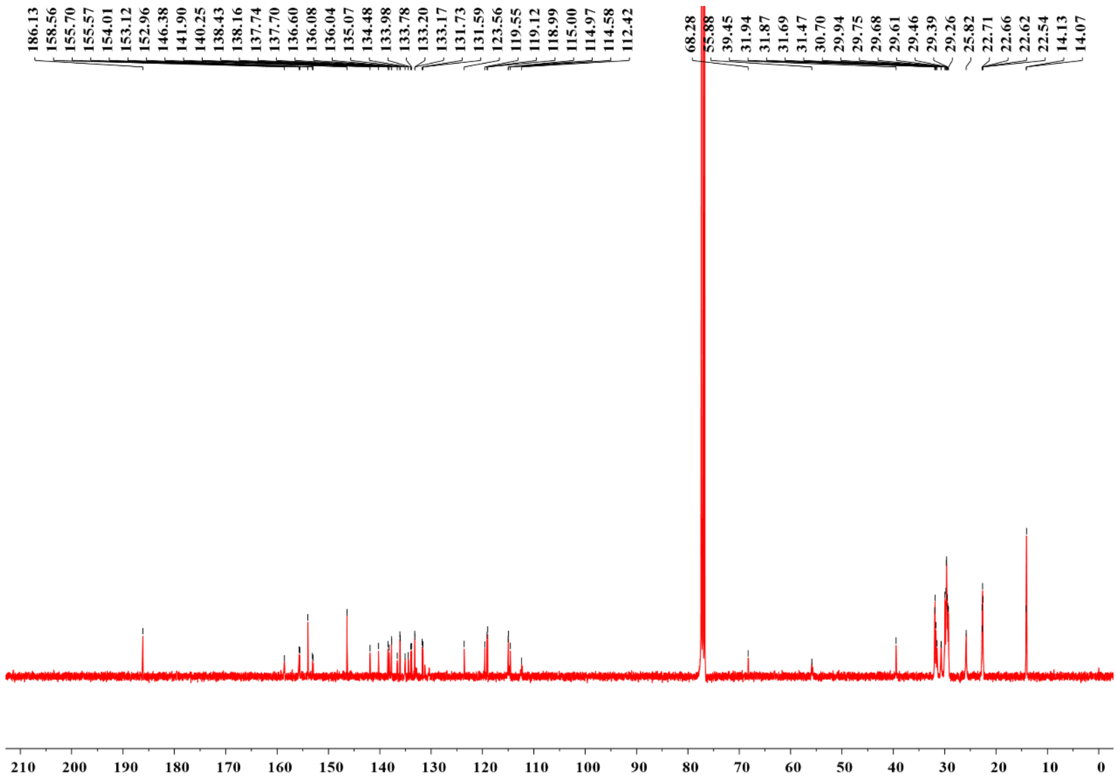

Supplementary Fig. 20 ^{13}C NMR spectrum of compound CH21 at 300K in $CDCl_3$.

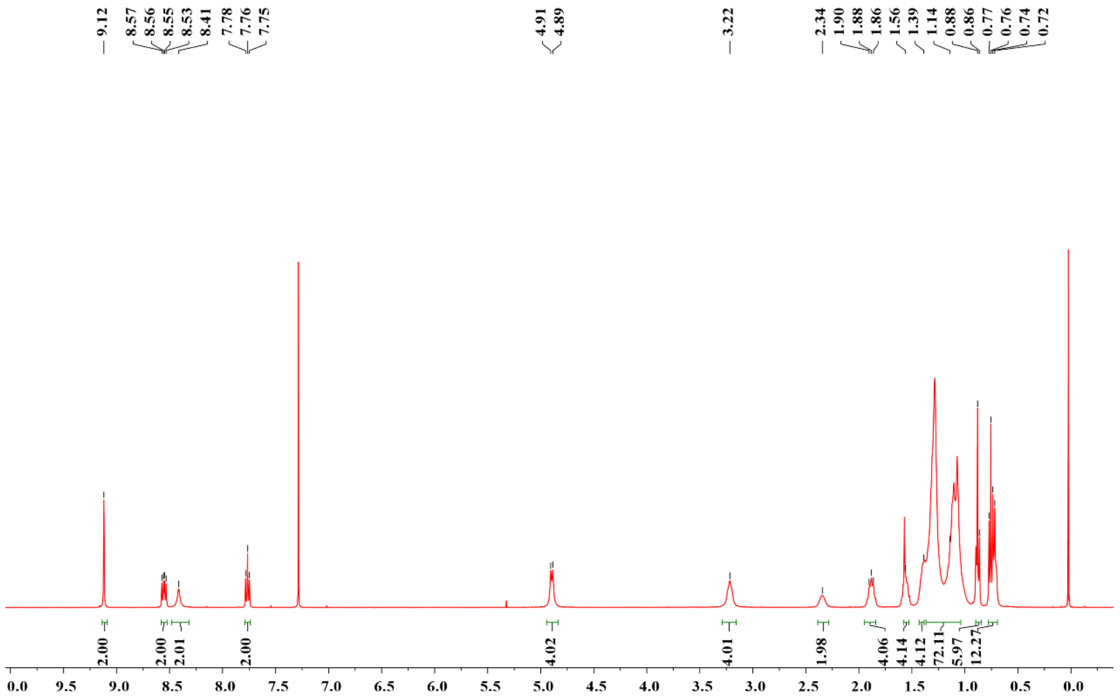

Supplementary Fig. 21 ^{1}H NMR spectrum of compound CH22 at 300K in $CDCl_3$.

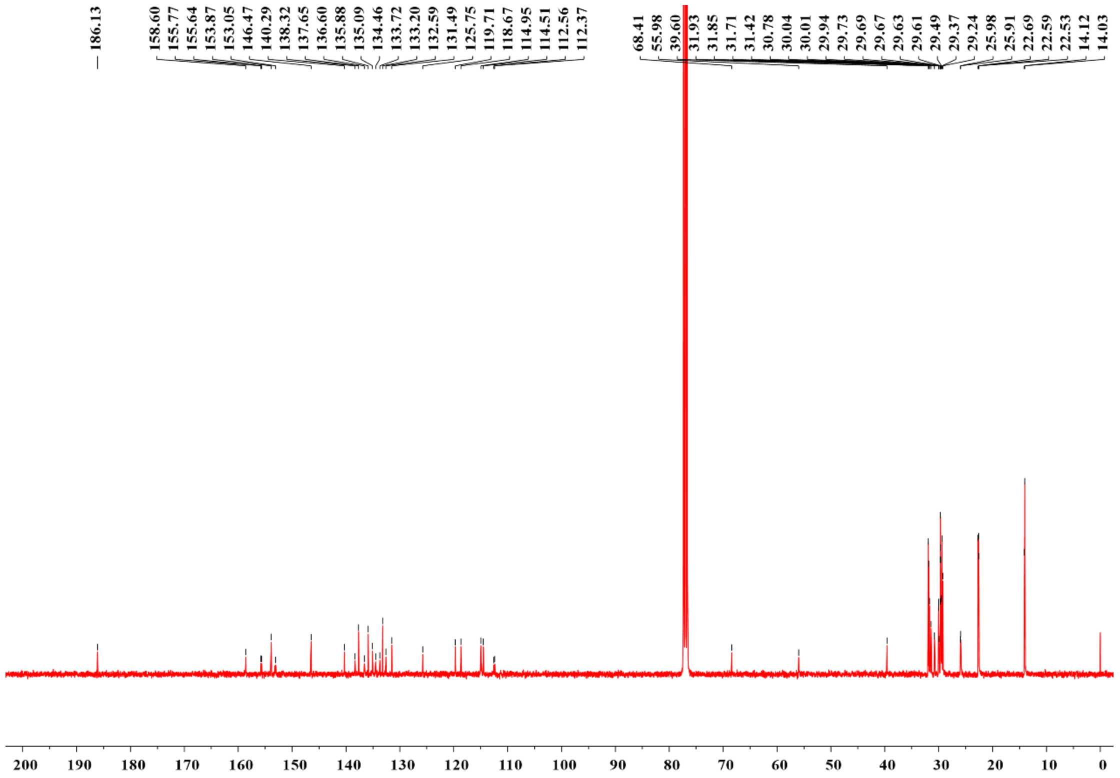

Supplementary Fig. 22 ^{13}C NMR spectrum of compound CH22 at 300K in $CDCl_3$.

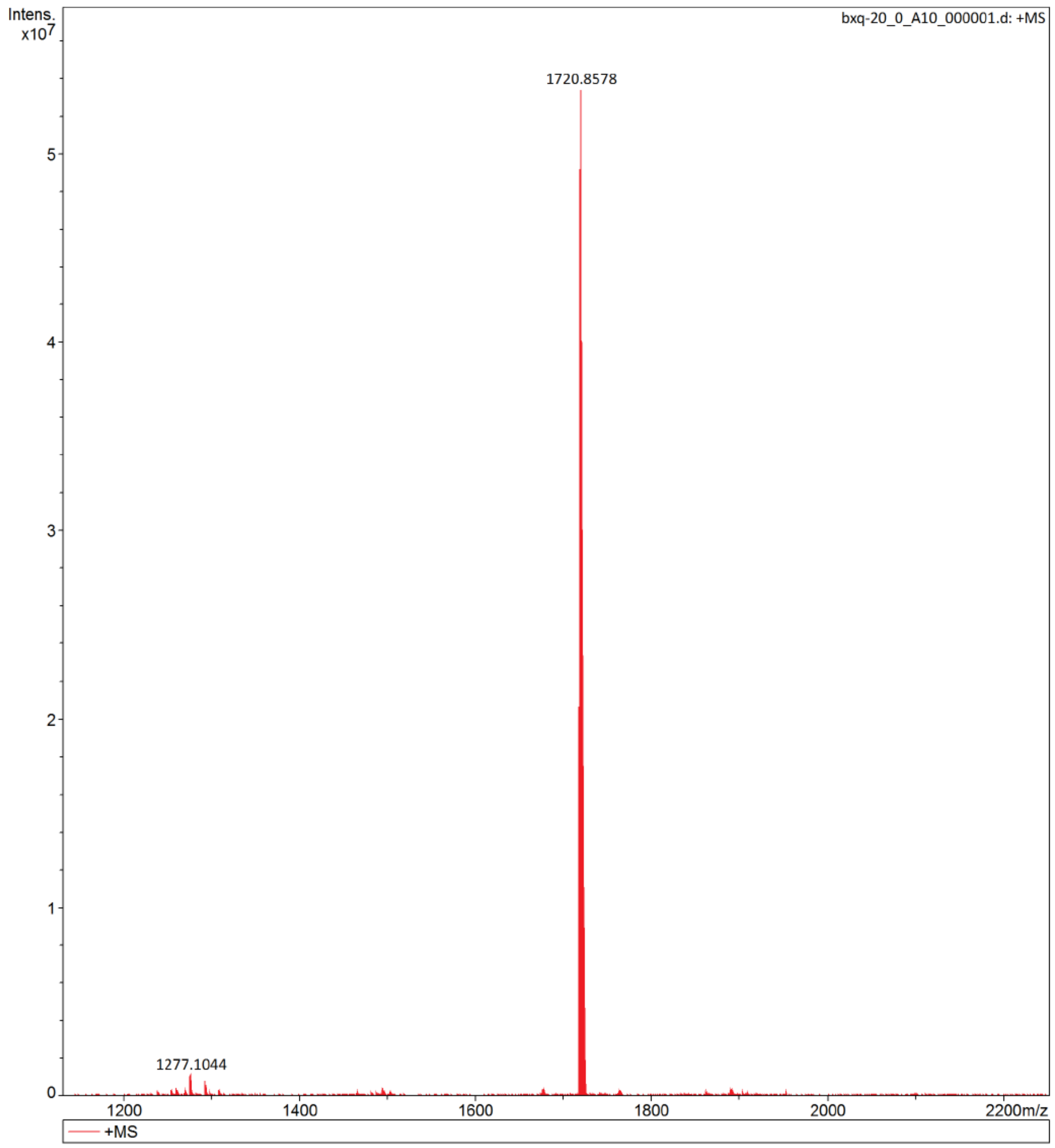

Supplementary Fig. 23 High resolution mass spectrometry (HRMS) of CH20.

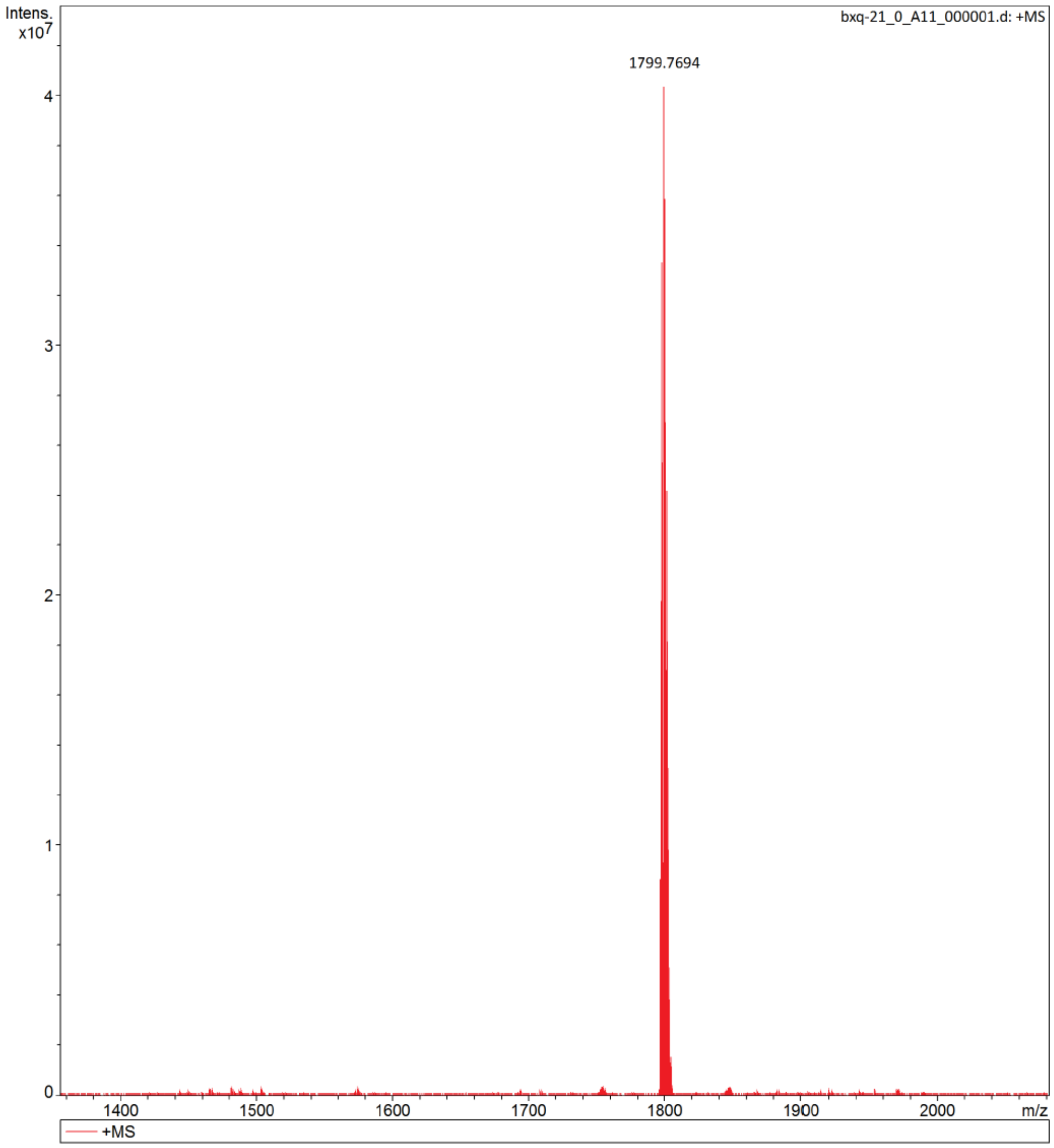

Supplementary Fig. 24 HRMS of CH21.

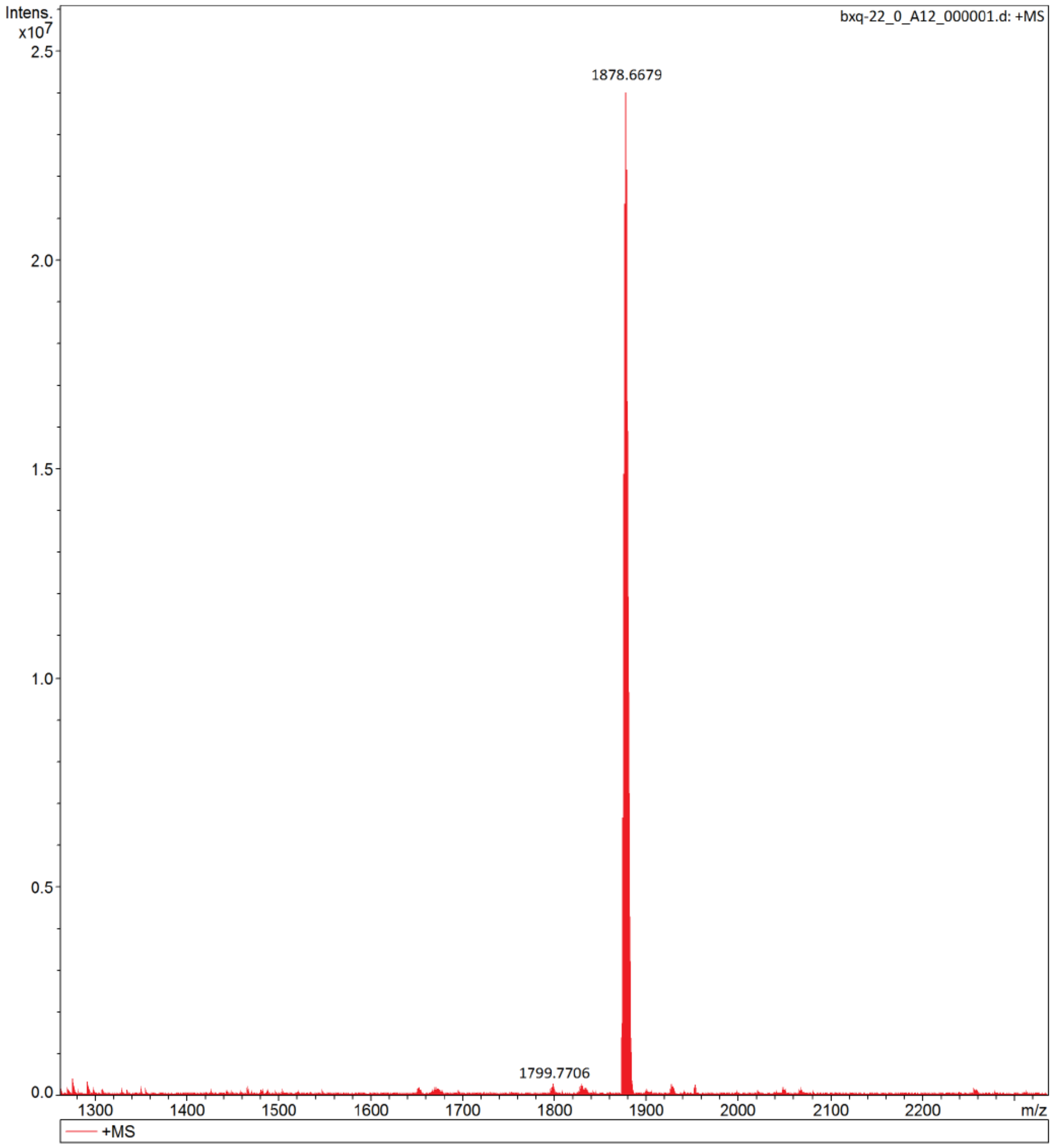

Supplementary Fig. 25 HRMS of CH22.

2. Supplementary Note 1

Computational Methods in This Work

The ground-state (S_0) geometries of studied molecules, energy level of frontier molecular orbital and the reorganization energy were optimized by density functional theory (DFT) using the Becke three-parameter Lee-Yang-Parr (B3LYP) hybrid functional.[1] The 6-31G(d)[2] and the quasi-relativistic pseudopotentials LANL2DZ[3] basis sets were employed for light C, H, O, N, S F atoms and heavy Br atom, respectively. All the alkyl chains were replaced with methyl groups ($-CH_3$) to reduce the computational requirements. And the vibrational frequencies were calculated after geometries optimization and no imaginary frequency was found. All the calculations were performed in the Gaussian 16 package.[4] The electronic coupling between dimer can be obtained by using Prof. Shuai's code based on the Supplementary Equation 1:

$$V=\frac{H_{12}-\frac{1}{2}(H_{11}+H_{22})S_{12}}{1-S_{12}^{2}} \qquad \text{(S1)}$$

Here, $H_{12}=\langle\psi_1|H|\psi_2\rangle$, $S_{12}=\langle\psi_1|\psi_2\rangle$, $H_{11}=\langle\psi_1|H|\psi_1\rangle$, $H_{22}=\langle\psi_2|H|\psi_2\rangle$. H is the Kohn-Sham Hamiltonian of the dimer, $\psi_{1/2}$ means the LUMO of the monomer in the dimer for electron transport, S_{12} is the overlap integral. We obtained the transfer integrals at DFT/PW91PW91/6-31G(d) level. Besides, the Mercury software was employed to obtain the total packing energy and the π-π stacking interaction energy were calculated in details.

3. Supplementary Note 2

Single-crystal Growth

Single crystals of CH20 were grown by the liquid diffusion method at room temperature. In detail, 1.5 mL of methanol was transferred to 0.15 mL of concentrated chloroform solution of CH20 slowly, and the beautiful cuboid-shape dark purple crystals were formed on the inner glassy tube after about 3 days. The X-ray diffraction signals of single crystal were collected on Rigaku XtalAB PRO MM007 DW. The crystal was kept at 193.0 K during data collection. The single crystal growth methods of CH21 and CH22 are the same as CH20.

4. Supplementary Note 3

UV-Visible (UV-vis) Absorption. The UV-vis spectra were obtained by a Cary 5000 UV-vis spectrophotometer.

Thermogravimetric Analysis (TGA) and Differential Scanning Calorimeter (DSC) analysis. The TGA analysis and the DSC were carried out on a TG209 DSC204 DMA242 TMA202 (NETZSCH) instrument with a heating rate of 5 °C min^{-1} under a nitrogen atmosphere.

Cyclic Voltammetry (CV). The CV experiments were performed with a LK98B II Microcomputer-based Electrochemical Analyzer. All measurements were conducted at room temperature with a three-electrode configuration. Among them, a glassy carbon electrode was employed as the working electrode, a saturated calomel electrode (SCE) was used as the reference electrode, and a Pt wire was used as the counter electrode. Tetrabutyl ammonium phosphorus hexafluoride (n-Bu_4NPF_6, 0.1 M) in acetonitrile was employed as the supporting electrolyte, and the scan rate was kept at 100 mV s^{-1}. Electrochemically reversible ferrocene was employed as internal reference. The HOMO and LUMO energy levels were calculated from the onset oxidation and the onset reduction potentials, respectively, by following the Supplementary Equation 2-3:

$$E_{\mathrm{HOMO}} = -\left(4.80 + E_{\mathrm{ox}}^{\mathrm{onset}}\right)\ \mathrm{eV} \qquad \text{(S2)}$$

$$E_{\mathrm{LUMO}} = -\left(4.80 + E_{\mathrm{re}}^{\mathrm{onset}}\right)\ \mathrm{eV} \qquad \text{(S3)}$$

Temperature-dependent Photoluminescence (PL) spectra.[5, 6] The temperature-dependent PL spectra varied from 130 to 300 K were conducted by using FLS1000 equipment. The E_b values were obtained by fitting the temperature-varying PL intensities, I(T), with the Supplementary Equation 4:

$$\mathrm{I(T)} = \frac{\mathrm{I_0}}{1 + \mathrm{A}e^{-\frac{E_b}{k_b T}}} \qquad \text{(S4)}$$

Where I_0 is the PL intensity at the lowest temperature; T is the temperature; A is a constant; k_b is the Boltzmann constant.

Exciton Binding Energy Calculations.[5, 6, 7] Exciton binding energy is defined as the difference between the fundamental transport gap (E_g^t) and the optical gap (E_g^O):

$$E_b = E_g^t - E_g^O \quad \text{(S5)}$$

Where, E_g^t is calculated as the energy difference between the ionization potential (IP) and electron affinity (EA), by following the Supplementary Equation 6-8:

$$E_g^t = \text{IP} - \text{EA} \quad \text{(S6)}$$

$$\text{IP} = E_+ - E_0 \quad \text{(S7)}$$

$$\text{EA} = E_0 - E_- \quad \text{(S8)}$$

Where E_0, E_+, and E_- denote the total potential energies of the ground state (S_0) and the cationic and anionic states, respectively. E_g^O is the excitation energy of the first singlet excited state (S_1). In the gas phase, the calculations were performed on the isolated molecules extracted directly from the optimized crystal structure. The S_0 and ionic states were calculated by DFT while the S_1 state was calculated by time-dependent DFT (TDDFT) with the long-range corrected (LRC) functional ωB97XD and the def2-SVP basis set.

Relative Dielectric Constant (ε_r) Test.[8, 9] The dielectric constant should be calculated in terms of the material's geometric capacitance, which represents the capacitance derived from only the material itself (the electronic, atomic, and ionic polarization). The capacitance-frequency of CH20, CH21 and CH22 neat and blended films were performed with a capacitor architecture of ITO/active layer/Ag at difference frequency from 100 Hz to 1M Hz using Zennium-E under dark conditions and analyzed with the Zahner Analysis software. Between 10^4 Hz and 2×10^5 Hz, a flat capacitive response with respect to frequency is obtained. Relative dielectric constant (ε_r) can be calculated according to the Supplementary Equation 9:

$$\varepsilon_r = \frac{C_p \times D}{A \times \varepsilon_0} \quad \text{(S9)}$$

Where C_p is the measured capacitance; D is the thickness of film; A is the contact area and ε_0 is the permittivity of free space.

Electroluminescence (EL) and Electroluminescence External Quantum Efficiency (EQE_{EL}). For the EQE_{EL} measurements, a digital source meter (Keithley 2400) was employed to inject electric current into the solar cells, and the emitted

photons were collected by a Si diode (Hamamatsu s1337-1010BQ) and indicated by a picometer (Keithley 6482). The injection current to the OSCs was kept at 1 mA by the direct current meter (PWS2326 Tectronix).

Fourier Transform Photocurrent Spectrometer EQE (FTPS-EQE). The FTPS-EQE measurement was carried out on an Enlitech FTPS PECT-600 instrument.

Measurements of Transient Photovoltage (TPV)/Transient Photocurrent (TPC). A white light bias was generated from an array of diodes (Molex 180081-4320) with light intensity about 0.5 sun. The light intensity of the diode pumped laser passing through an attenuator is about 1132.5 μW cm^{-2}. A diode pumped laser (Lapa-80) was used as the perturbation source, with a pulse duration of 10 ns and a repetition frequency of 20 Hz. The perturbation light intensity was attenuated to keep the amplitude of transient V_{OC} (ΔV_{OC}) below 10 mV so that $\Delta V_{OC} \ll V_{OC}$. Voltage and current dynamics were recorded on a digital oscilloscope (Tektronix MDO4104C), and voltages at open circuit and currents under short circuit conditions were measured over a 1M Ω and a 50 Ω resistor, respectively.

Space-Charge-Limited Current (SCLC) Measurement. The SCLC method was used to measure the hole and electron mobilities, by using a diode configuration of ITO/PEDOT:PSS/active layer/MoO_3/Ag for hole and ITO/ZnO/active layer/PNDIT-F3N/Ag for electron. The SMAs were fully dissolved in CF with 15 mg mL^{-1} and then the solutions were stirred 4 hours at room temperature and spin-casted at 1500 rpm for 30s. After spin-coating, the neat films were annealed at 80 °C for 5 mins. The fabrication method of blended films was consistent with that of active layer of device. The dark current density curves were recorded with a bias voltage in the range of 0~8 V. The mobilities were estimated by taking current-voltage curves and fitting the results based on the Supplementary Equation 10 listed below:

$$J=\frac{9\varepsilon_0\varepsilon_r\mu V^2}{8L^3} \tag{S10}$$

Where J is the current density, ε_0 is the vacuum permittivity, ε_r is the relative dielectric constant, μ is the mobility, and L is the film thickness. $V=(V_{app}-V_{bi})$ is the internal voltage in the device, where V_{app} is the applied voltage to the device and V_{bi} is the

built-in voltage due to the relative work function difference between the two electrodes.

Atomic Force Microscopy (AFM) and Transmission Electron Microscopy (TEM). The AFM images were performed using in tapping mode on a Bruker Dimension Icon atomic force microscope. The TEM was performed on a Talos F200X G2.

Grazing Incidence Wide Angle X-ray Scattering (GIWAXS). The GIWAXS samples were prepared on Si/PEDOT:PSS substrates by use of the same preparation conditions with devices and were carried out at XEUSS SAXS/WAXS equipment.

The Calculation of Energy Loss. The E_g of the blended films were calculated from the intersection of the normalized UV-vis absorption and PL spectra of acceptors.[10, 11] In general, the total E_{loss} was calculated by using the Supplementary Equation 11:

$$E_{loss}=E_g-qV_{oc} \tag{S11}$$

The total E_{loss} can be divided into three parts: the energy loss during the CT process or the formation of CT state at D/A interfaces (ΔE_{CT}), the radiative recombination loss ($q\Delta V_r$) and the non-radiative recombination loss ($q\Delta V_{nr}$). E_{CT} is obtained by fitting the low-energy region of highly sensitive EQE and EL spectra.

$$\Delta E_{CT}=E_g-E_{CT} \tag{S12}$$

$V_{OC,rad}$ is the radiative recombination limit for the V_{OC} of the solar cell, which can be determined by the Supplementary Equation 13:

$$V_{OC,rad}\approx\frac{kT_a}{q}\ln\left(\frac{J_{ph}}{J_{0,rad}}\right) \tag{S13}$$

Where, the $J_{0,rad}$ values calculated by the Supplementary Equation 14:

$$J_{0,rad}=q\int_0^{+\infty}\mathrm{EQE_{PV}(E)}\phi_{BB}\mathrm{(E)dE} \tag{S14}$$

Thus, ΔV_r and ΔV_{nr} can be determined by the Supplementary Equation 15-16:

$$q\Delta V_r=qE_{CT}-qV_{OC,rad} \tag{S15}$$

$$q\Delta V_{nr}=qV_{OC}^{rad}-qV_{OC} \tag{S16}$$

5. Supplementary Figures 26-56 and Supplementary Tables 1-18

Supplementary Table 1. Comparison of brominated OSCs performance between this work and references.

Active layer	V_{OC} (V)	J_{SC} (mA cm^{-2})	FF (%)	PCE (%)	Ref.
PTPDBDT:Br-ITIC	0.93	15.4	66.00	9.4	16
PBDB-T:F-Br	0.87	18.22	76.00	12.05	17
PBDB-T:IT-2Br	0.83	17.93	71.00	10.66	18
PM6:ITIC-2Br-γ	0.89	19.01	71.21	12.05	19
PM6:ITC-2Br2	0.90	19.8	73.80	13.10	20
PBDB-T:BTTPC-Br	0.86	24.71	71.00	15.22	21
PM6:TSeIC4Br	0.77	21.27	72.40	11.92	22
PM7:BDSe-2(BrCl)	0.83	22.91	76.50	14.54	23
PM6:ZB	0.90	26.38	64.16	15.23	24
PM6:BTIC-2Br-m	0.88	25.03	73.13	16.11	25
PM6:BTP-ClBr	0.906	23.48	79.00	16.82	26
PM6:C8IDT-Br	0.97	15.66	64.81	9.85	27
PM6:BTIC-4EO-4Br	0.84	22.78	65.21	12.41	28
PM6:BTP-(Br,Me)-1	0.92	21.38	68.25	13.43	29
PM6:IT-2Br	0.845	21.98	69.53	12.92	30
PTQ10:IDIC-Br	0.92	16.3	65.00	10.80	31
PBDB-T:6TIC-2Br	0.76	22.74	68.27	11.77	32
D18:F-ThBr	1.089	16.68	71.69	13.03	33
PM6:Y-BO-FBr	0.85	25.83	75.02	16.47	34
PM6:BTP-H2	0.932	25.33	78.50	18.50	35
PM6:CH22-6Br	0.871	26.25	74.31	17.00	This work
PM6:CH21	0.873	26.57	78.13	18.12	This work
PM6:CH22	0.884	26.74	80.62	19.06	This work

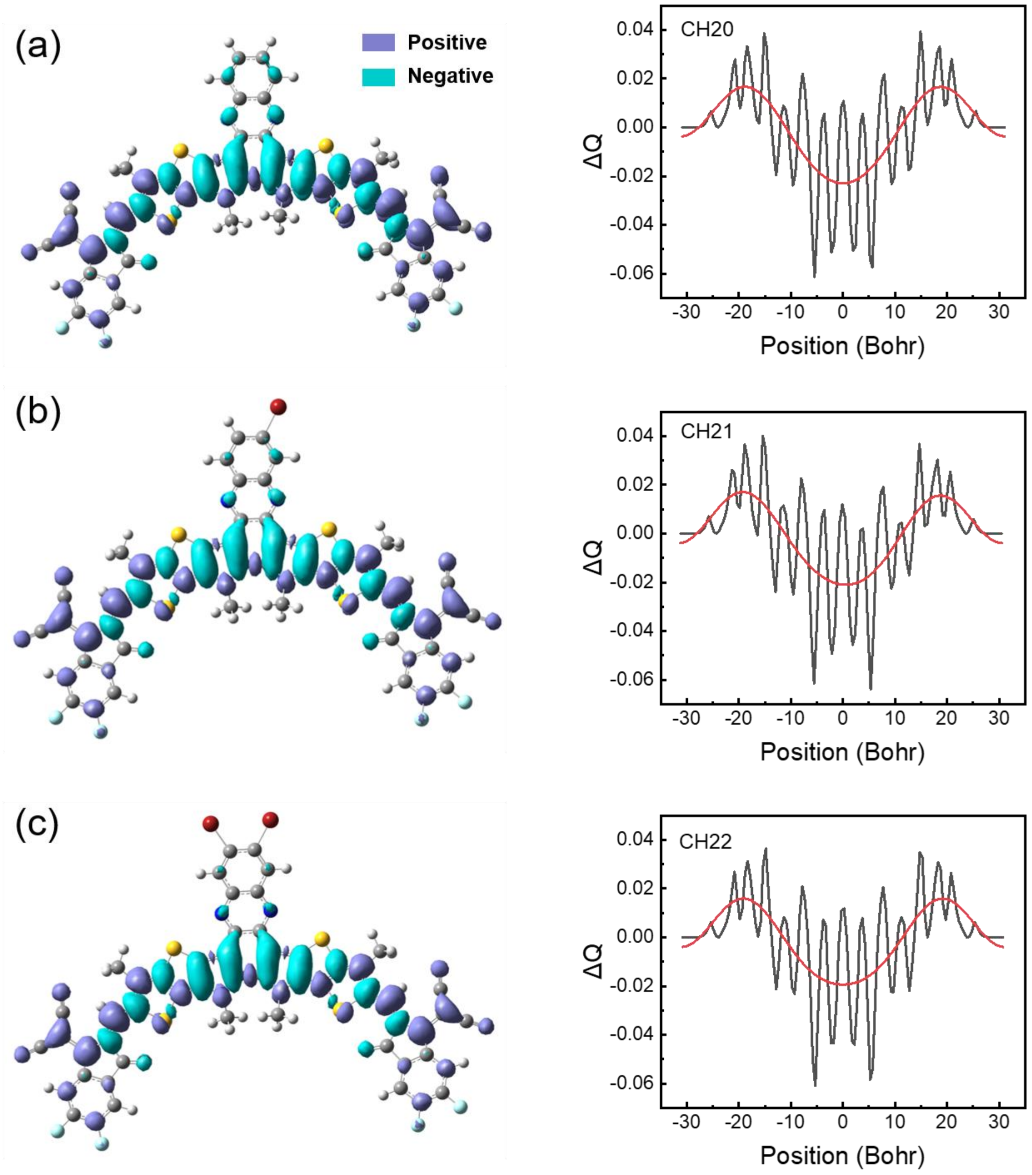

Supplementary Fig. 26 Theoretical density distribution ΔQ. ΔQ ($\Delta Q = \Psi^2_{LUMO} - \Psi^2_{HOMO}$) along the longest axis (backbone) of (a) CH20, (b) CH21 and (c) CH22.[12] Black curves are the integral lines of the charge density difference (ΔQ) along the longest axis (backbone) of the defined molecules. Red curves are the simulated results with low frequencies wave functions by fast flourier transform filters.

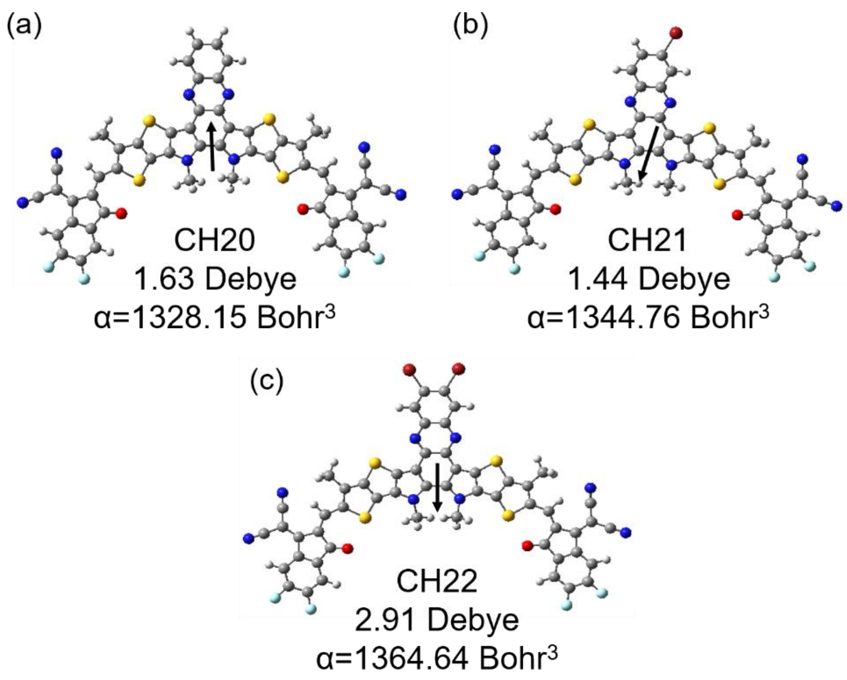

Supplementary Fig. 27 The isotropic polarizability and dipole moment of (a) CH20, (b) CH21 and (c) CH22.

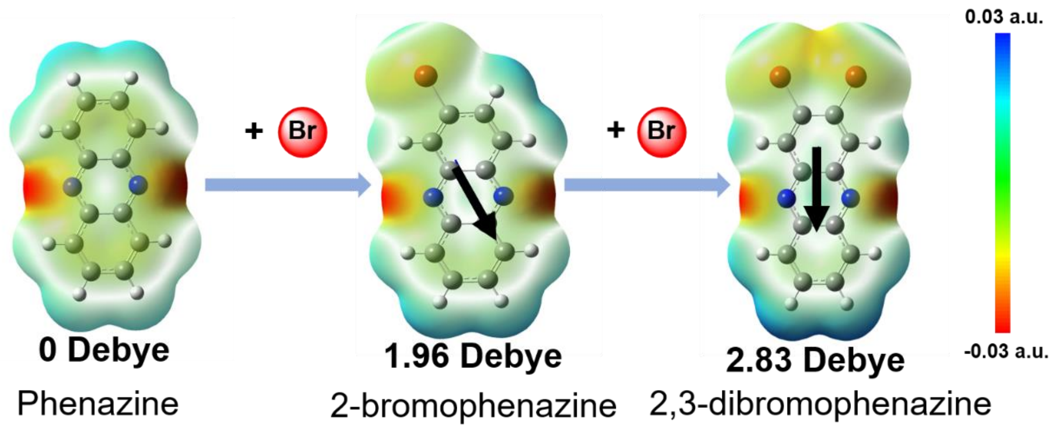

Supplementary Fig. 28 Electrostatic surface potential (ESP) maps of central units for CH20, CH21 and CH22.

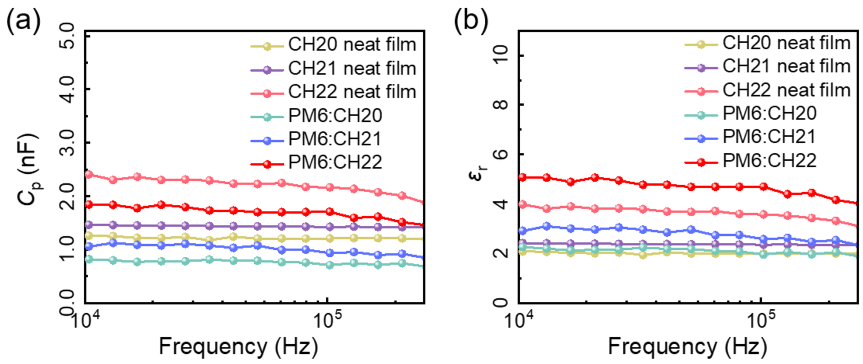

Supplementary Fig. 29 Relative Dielectric Constant(ε_r) Test. (a) Flat C_p response of CH20, CH21 and CH22 neat films and blended films at different frequency. (b) ε_r as a function of frequency measured by impedance spectroscopy.

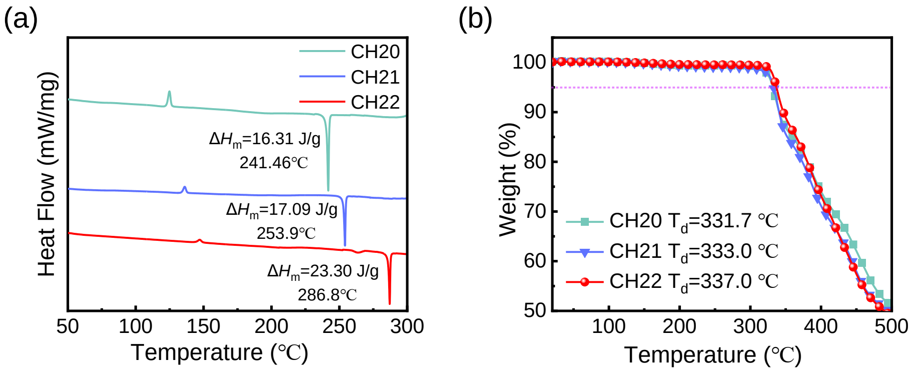

Supplementary Fig. 30 DSC and TGA. (a) Differential Scanning Calorimeter (DSC) curves of CH20, CH21 and CH22. (b) Thermogravimetric analysis (TGA) curves of CH20, CH21 and CH22.

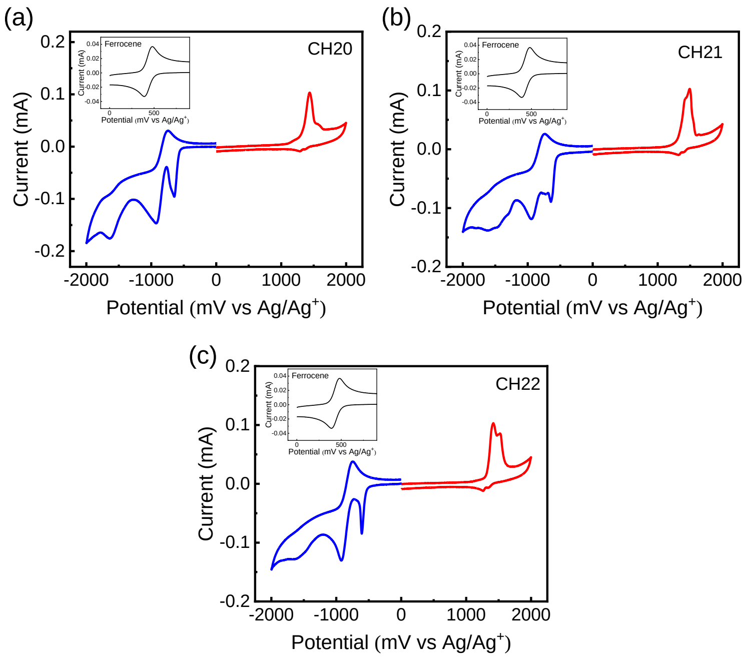

Supplementary Fig. 31 Cyclic voltammograms of (a) CH20, (b) CH21 and (c) CH22 films. Red line: oxidation cycle, blue line: reduction cycle.

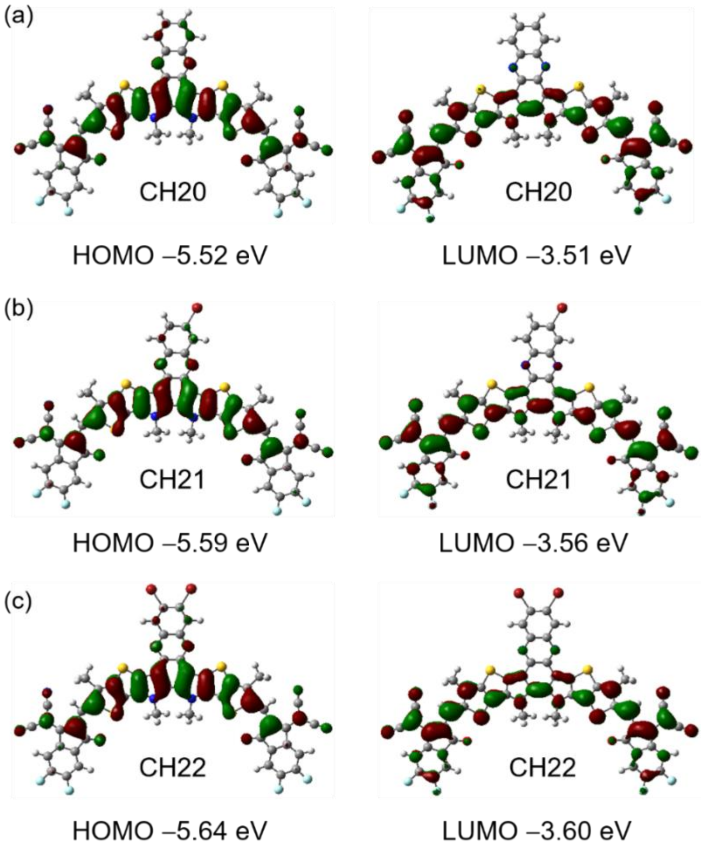

Supplementary Fig. 32 Theoretical density distribution for the frontier molecular orbits of (a) CH20, (b) CH21 and (c) CH22. Note that the relative alignment of energy levels derived from CVs is in accordance with the results from theoretical calculations.

Supplementary Table 2. The optical and electrochemical properties of CH20, CH21 and CH22 SMAs.

SMAs	λ_{max} [a] (nm)	ε [b] (10^5 L mol^{-1}cm^{-1})	λ_{max} [c] (nm)	λ_{onset} [c] (nm)	E_g^{opt} [d] (eV)	HOMO^{CV} [e] (eV)	LUMO^{CV} [e] (eV)
CH20	740	2.04	824	914	1.36	–5.61	–3.80
CH21	733	2.13	821	905	1.37	–5.63	–3.80
CH22	729	2.20	813	892	1.39	–5.67	–3.83

[a]In the chloroform solution. [b]The molar absorption coefficient of CH20, CH21, CH22 in the chloroform solution. [c]In the neat films. [d]Optical band gap was calculated by $1240/\lambda_{onset}^{film}$. [e]The highest occupied molecular orbital (HOMO) and lowest unoccupied molecular orbital (LUMO) energy levels were calculated from the onset oxidation potential and the onset reduction potential using the equation: $E_{HOMO} = -(4.80 + E_{ox}^{onset})\ eV$, $E_{LUMO} = -(4.80 + E_{re}^{onset})\ eV$.

Supplementary Table 3. Capacitance, film thickness, device area and dielectric constant at 2×10^5 Hz of CH20, CH21, CH22 neat films and blended films. [a]

Materials	C (10^{-9}F)	d (nm)	A (mm^2)	$\varepsilon_{r\ max}$	$\varepsilon_{r\ avg}$
CH20	1.15±0.05	60	4.10	2.00	1.91±0.08
CH21	1.39±0.02	60	4.10	2.35	2.30±0.03
CH22	1.93±0.05	60	4.10	3.32	3.19±0.09
PM6:CH20	0.72±0.02	100	4.10	2.06	1.98±0.06
PM6:CH21	0.89±0.03	100	4.10	2.54	2.45±0.08
PM6:CH22	1.42±0.08	100	4.10	4.17	3.92±0.22

[a]The average relative dielectric constant calculated from 5 independent devices.

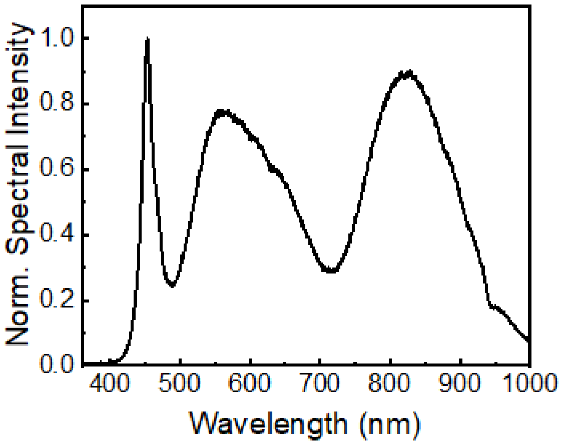

Supplementary Fig. 33 Light spectrum of the LEDs used for photo stability test in this work as extracted from the company's datasheet.

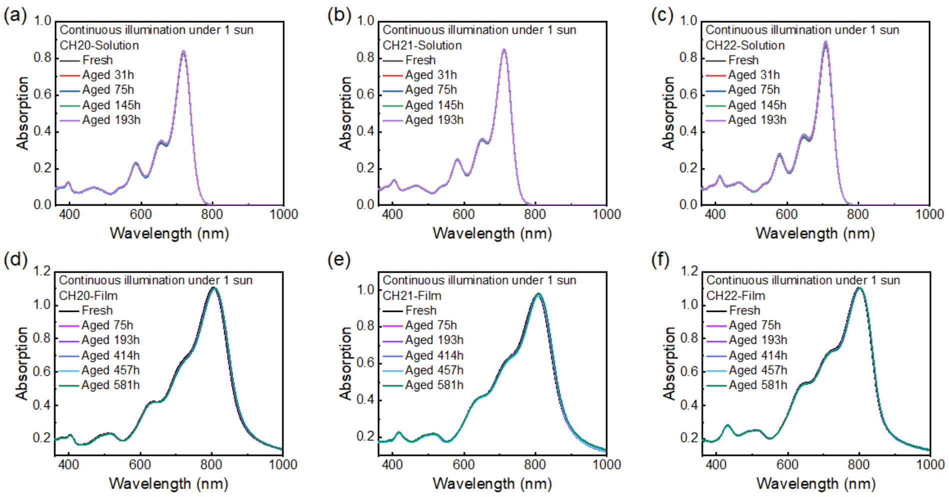

Supplementary Fig. 34 Photo stability. UV-vis absorption spectra plotted vs. aging time under continuous illumination of (a-c) CH20, CH21 and CH22 in dilute toluene solutions and (d-f) CH20, CH21 and CH22 in films.

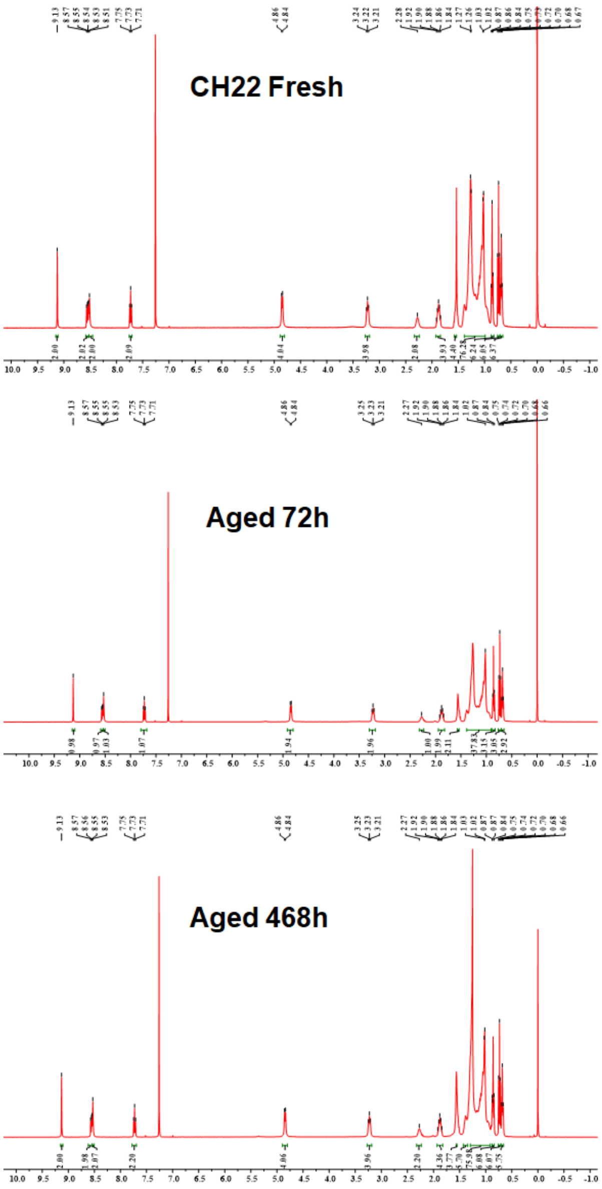

Supplementary Fig. 35 Photo stability. ^{1}H NMR spectra of CH22 fresh and aged samples in $CDCl_3$. The samples of spin-coated films of CH22 were aged under continuous illumination for 468 h, then collected and dried before conducting ^{1}H NMR.

Supplementary Table 4. Crystal data and structure refinement for CH20, CH21 and CH22.

Compound	CH20	CH21	CH22
Empirical formula	$C_{104}H_{122}F_4N_8O_2S_4$	$C_{104}H_{121}BrF_4N_8O_2S_4$	$C_{104}H_{120}Br_2F_4N_8O_2S_4$
Formula weight	1720.33	1799.23	1878.13
Temperature/k	193.0	193.0	193.0
Crystal system	triclinic	triclinic	triclinic
Space group	P-1	P-1	P-1
Cell a/Å	13.2412(10)	15.5364(10)	15.4802(4)
b/Å	17.7848(10)	17.0821(13)	17.2861(5)
c/Å	21.2417(10)	19.3025(14)	19.2225(5)
α/°	93.403(4)	97.034(4)	96.771(2)
β/°	103.507(5)	102.772(4)	102.9300(10)
γ/°	99.261(5)	102.220(4)	101.9740(10)
Volume/$Å^3$	4775.9(5)	4805.2(6)	4830.9(2)
Z	2	2	2
ρ_{calc}, g/cm^{-3}	1.196	1.244	1.288
μ/mm^{-1}	1.394	1.231	1.544
F(000)	1836.0	1904.0	1968.0
Crystal size/mm^3	0.13×0.12×0.1	0.12×0.11×0.1	0.15×0.12×0.1
Radiation	CuKα (λ=1.54178)	GaKα (λ = 1.34139)	GaKα (λ=1.34139)
2Θ range for data collection/°	5.06 to 136.484	6.786 to 115.212	4.616 to 114.274
Index ranges	$-15 \leq h \leq 15$, $-19 \leq k \leq 21$, $-25 \leq l \leq 25$	$-19 \leq h \leq 19$, $-21 \leq k \leq 15$, $-23 \leq l \leq 24$	$-17 \leq h \leq 19$, $-21 \leq k \leq 21$, $-23 \leq l \leq 24$
Reflections collected	48102	49324	55804
Independent reflections	17068 [R_{int}=0.1084, R_{sigma}=0.1304]	19555 [Rint=0.0673, Rsigma=0.0988]	19703 [R_{int}=0.0723, R_{sigma}=0.0808]
Data/restraints/parameters	17068/426/1105	19555/475/1122	19703/775/1211
Goodness-of-fit on F^2	1.120	1.067	1.099
Final R indexes [I>=2σ (I)]	R_1=0.1330, wR_2=0.3100	R_1=0.1498, wR_2=0.3473	R_1=0.1300, wR_2=0.3079
Final R indexes [all data]	R_1=0.2034, wR_2=0.3544	R_1=0.2286, wR_2=0.3810	R_1=0.2058, wR_2=0.3445
Largest diff. peak/hole/e $Å^{-3}$	0.77/–0.82	1.15/–0.87	1.42/–1.23

The X-ray diffraction signals of single crystals were collected on Rigaku XtalAB PRO MM007 DW at 193 K.

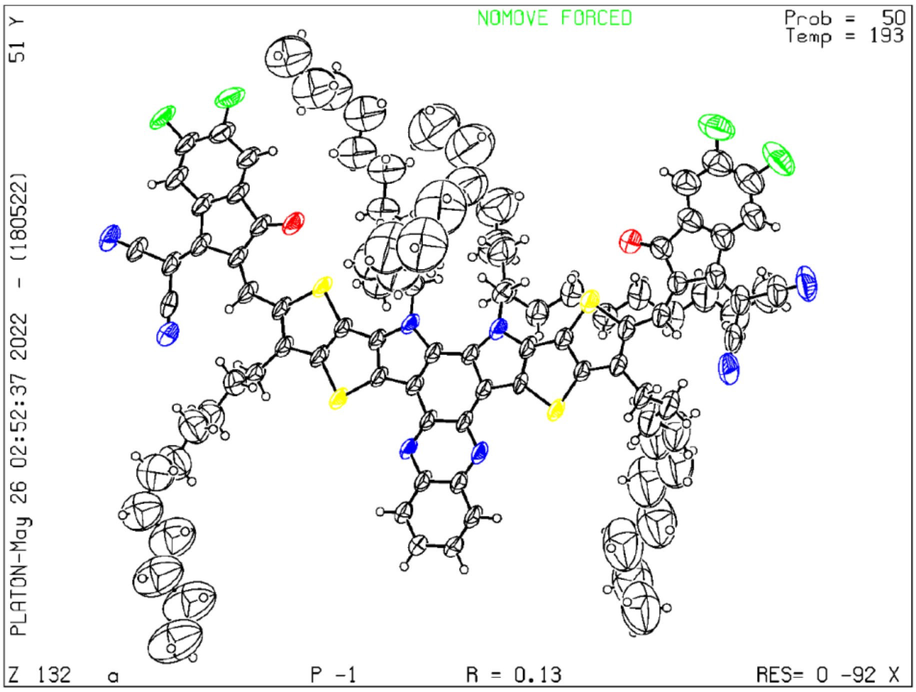

Supplementary Fig. 36 The ORTEP-style illustration with probability ellipsoids of CH20 (CCDC: 2244842).

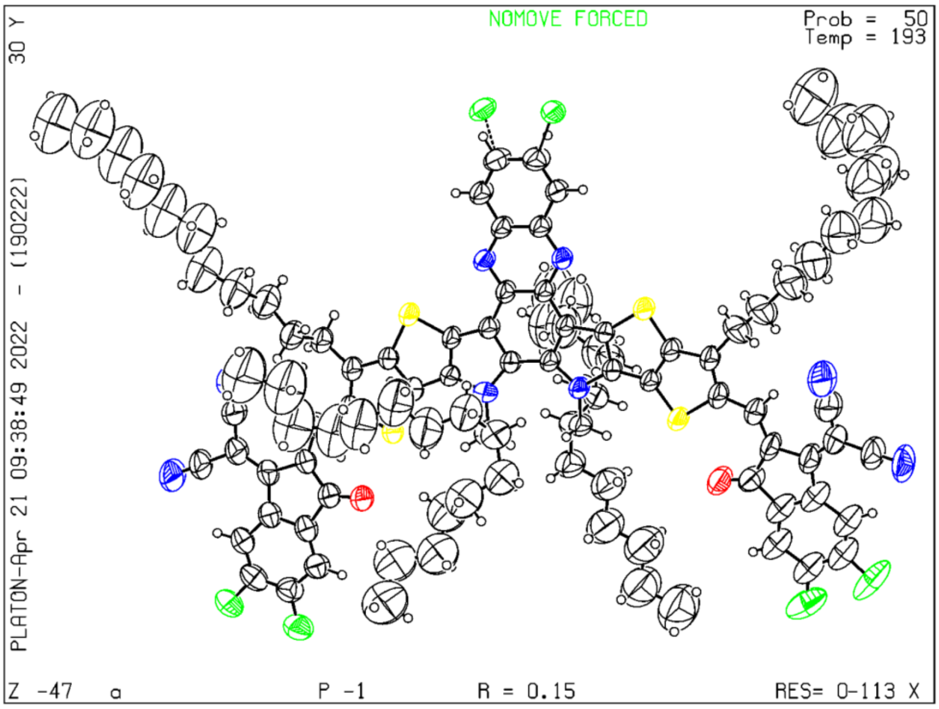

Supplementary Fig. 37 The ORTEP-style illustration with probability ellipsoids of CH21 (CCDC: 2244846).

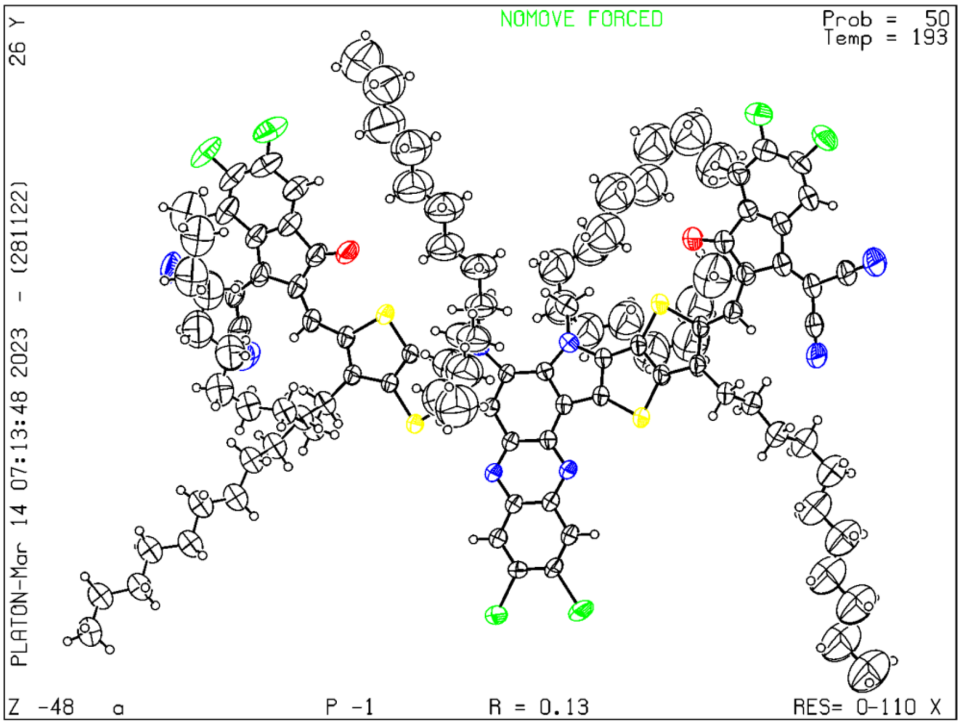

Supplementary Fig. 38 The ORTEP-style illustration with probability ellipsoids of CH22 (CCDC: 2244848).

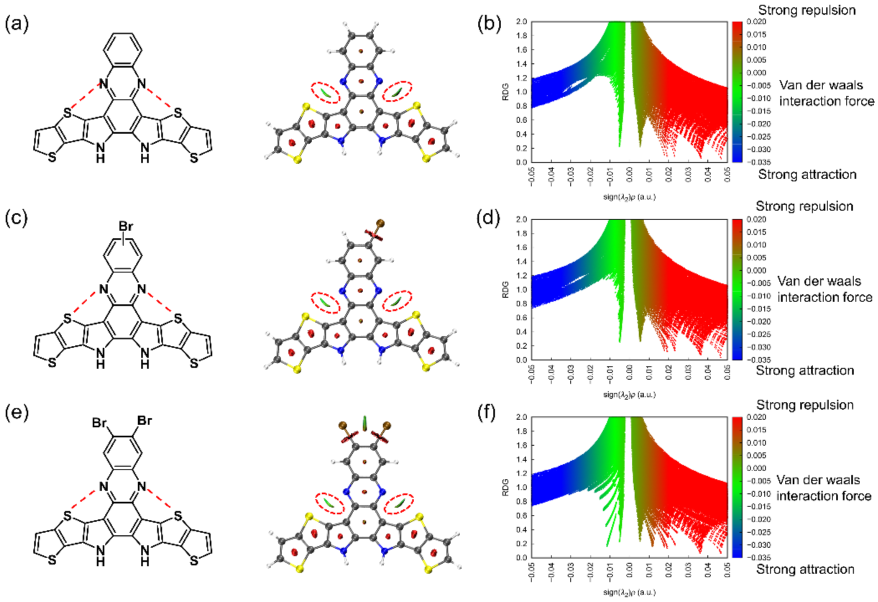

Supplementary Fig. 39 Reduced density gradient (RDG) versus sign(λ_2)ρ with a (RDG) iso-surface of the central phenazine cores and bridged thiophene structures of (a, b) CH20, (c, d) CH21 and (e, f) CH22.

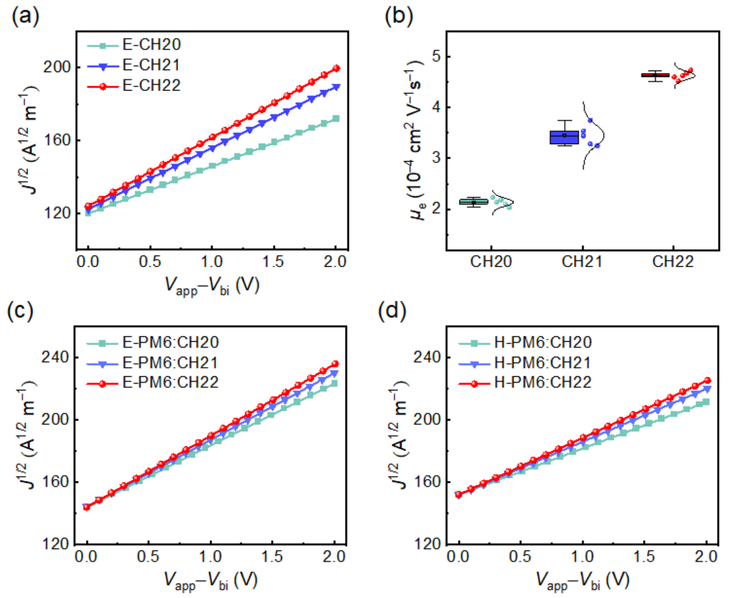

Supplementary Fig. 40 Mobilities of the neat and blended films of three SMAs. (a) Electron mobilities of the three SMAs. (b) Electron mobilities distributions counted by 5 devices of the three SMAs. (c) Electron mobilities and (d) hole mobilities of the blended films.

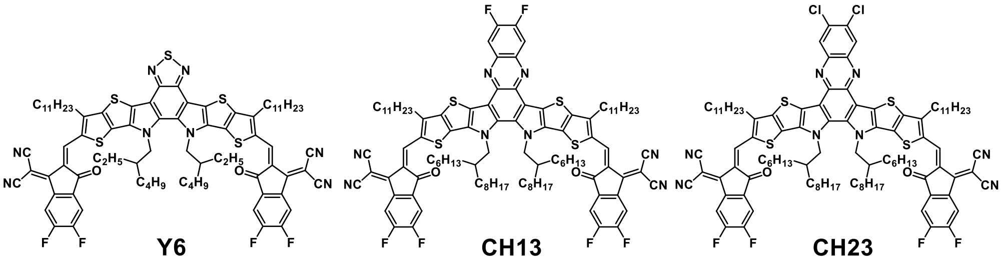

Supplementary Fig. 41 Molecular structures of Y6, CH13 and CH23.

Supplementary Table 5. Ionization potential (IP), electron affinity (EA), transport gap (E_g^t), optical gap (E_g^O), and exciton binding energy (E_b) of Y6, CH20, CH13, CH23, CH22 calculated in gas phase.

Molecule	IP/eV	EA/eV	E_g^t/eV	E_g^O/eV	E_b/eV
Y6	6.374	2.844	3.531	1.803	1.728
CH20	6.243	2.791	3.452	1.792	1.660
CH13	6.317	2.845	3.471	1.842	1.630
CH23	6.346	2.882	3.464	1.845	1.620
CH22	6.354	2.890	3.464	1.846	1.617

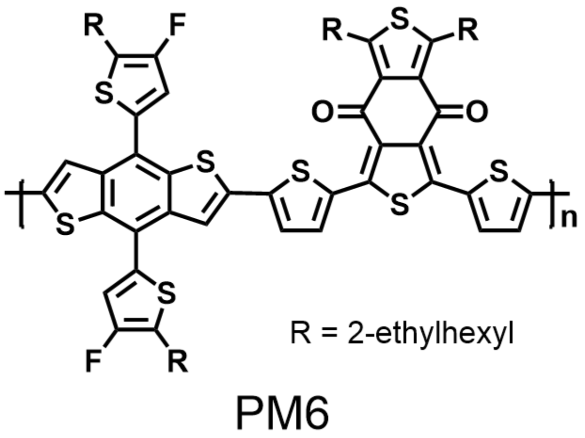

Supplementary Fig. 42 Chemical structure of PM6.

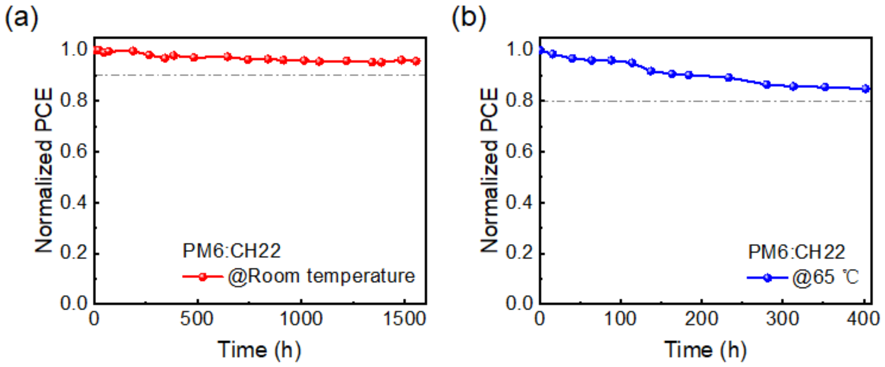

Supplementary Fig. 43 Stability. PCE variation versus operating time in a glovebox filled with nitrogen at (a) room temperature and (b) 65 ℃ of PM6:CH22-based devices.

Supplementary Table 6. Detailed photovoltaic parameters of the PM6:CH22 based devices processed by varied thermal annealing temperature under the illumination of AM 1.5 G, 100 mW cm^{-2}. [a]

TA (°C)	V_{OC} (V)	J_{SC} (mA cm^{-2})	FF (%)	PCE (%)
w/o	0.900	25.38	74.26	16.97
75	0.887	25.46	74.59	16.84
**80**	**0.887**	**25.85**	**75.56**	**17.32**
90	0.884	25.79	74.84	17.06

[a]The device architecture is ITO/PEDOT:PSS/active layer/PNDIT-F3N/Ag, D_{donor}=6 mg mL^{-1} in chloroform, the D:A ratio was 1:1.2, the resulting active layer solutions were spin-casted at 1800 rpm for 30 s.

Supplementary Table 7. Detailed photovoltaic parameters of the PM6:CH22 based devices processed by varied D:A ratios under the illumination of AM 1.5 G, 100 mW cm^{-2}. [a]

D:A (w/w)	V_{OC} (V)	J_{SC} (mA cm^{-2})	FF (%)	PCE (%)
1:1	0.887	25.46	74.59	16.84
1:1.1	0.887	25.58	75.85	17.22
**1:1.2**	**0.887**	**25.79**	**76.01**	**17.38**
1:1.3	0.881	25.83	75.09	17.09

[a]The device architecture is ITO/PEDOT:PSS/active layer/PNDIT-F3N/Ag, D_{donor}=6 mg mL^{-1} in chloroform, and the thermal annealing temperature was 80 °C.

Supplementary Table 8. Detailed photovoltaic parameters of the PM6:CH22 based devices processed by varied addition ratio of 1-CN under the illumination of AM 1.5 G, 100 mW cm^{-2}. [a]

1-CN (v/v)	V_{OC} (V)	J_{SC} (mA cm^{-2})	FF (%)	PCE (%)
0.2%	0.888	26.28	78.68	18.36
**0.4%**	**0.884**	**26.74**	**80.62**	**19.06**
0.6%	0.878	26.59	78.93	18.43

[a]The device architecture is ITO/PEDOT:PSS/active layer/PNDIT-F3N/Ag, D_{donor}=6 mg mL^{-1} in chloroform, the D:A ratio was 1:1.2, the resulting active layer solutions were spin-casted at 1800 rpm for 30 s, and the thermal annealing temperature was 80 °C.

Supplementary Table 9. Detailed photovoltaic parameters of the PM6:CH20 based devices by optimal conditions under the illumination of AM 1.5 G, 100 mW cm^{-2}.

Active layer	V_{OC} (V)	J_{SC} (mA cm^{-2})	FF (%)	PCE (%)
PM6:CH20	0.885	25.17	74.48	16.59
	0.884	25.26	74.27	16.58
	0.882	25.30	74.24	16.57
	0.884	25.40	73.95	16.60
	0.881	25.44	74.92	16.79
	0.879	25.51	74.62	16.73
	0.878	25.28	75.02	16.65
	0.878	25.47	74.56	16.67
	0.882	25.13	74.83	16.59
	0.880	24.90	74.91	16.41
	0.882	25.07	74.70	16.52
	0.880	25.12	74.88	16.55
	0.880	25.29	74.58	16.60
	0.878	25.34	74.53	16.58
	0.878	25.38	74.78	16.66
Average	0.881	25.27	74.62	16.61

Supplementary Table 10. Detailed photovoltaic parameters of the PM6:CH21 based devices by optimal conditions under the illumination of AM 1.5 G, 100 mW cm^{-2}.

Active layer	V_{OC} (V)	J_{SC} (mA cm^{-2})	FF (%)	PCE (%)
PM6:CH21	0.875	26.66	77.57	18.10
	0.874	26.46	77.79	17.99
	0.874	26.48	77.90	18.03
	0.874	26.51	77.47	17.95
	0.874	26.54	77.62	18.00
	0.870	26.62	77.70	17.99
	0.872	26.57	77.58	17.97
	0.870	26.67	77.62	18.01
	0.873	26.57	78.13	18.12
	0.873	26.56	76.64	17.77
	0.871	26.60	77.40	17.93
	0.869	26.57	77.57	17.91
	0.872	26.48	77.92	17.99
	0.873	26.59	77.02	17.88
	0.873	26.70	77.41	18.04
Average	0.872	26.57	77.56	17.98

Supplementary Table 11. Detailed photovoltaic parameters of the PM6:CH22 based devices by optimal conditions under the illumination of AM 1.5 G, 100 mW cm^{-2}.

Active layer	V_{OC} (V)	J_{SC} (mA cm^{-2})	FF (%)	PCE (%)
PM6:CH22	0.885	26.78	80.19	19.01
	0.882	26.56	80.28	18.81
	0.884	26.53	80.23	18.82
	0.881	26.68	80.37	18.89
	0.884	26.56	80.44	18.89
	0.885	26.53	80.37	18.87
	0.884	26.74	80.62	19.06
	0.883	26.47	80.53	18.82
	0.884	26.61	80.46	18.93
	0.881	26.83	79.81	18.87
	0.885	26.55	80.68	18.96
	0.887	26.40	80.54	18.87
	0.887	26.84	79.93	19.03
	0.885	26.57	80.25	18.88
	0.883	26.60	80.43	18.89
Average	0.884	26.62	80.34	18.91

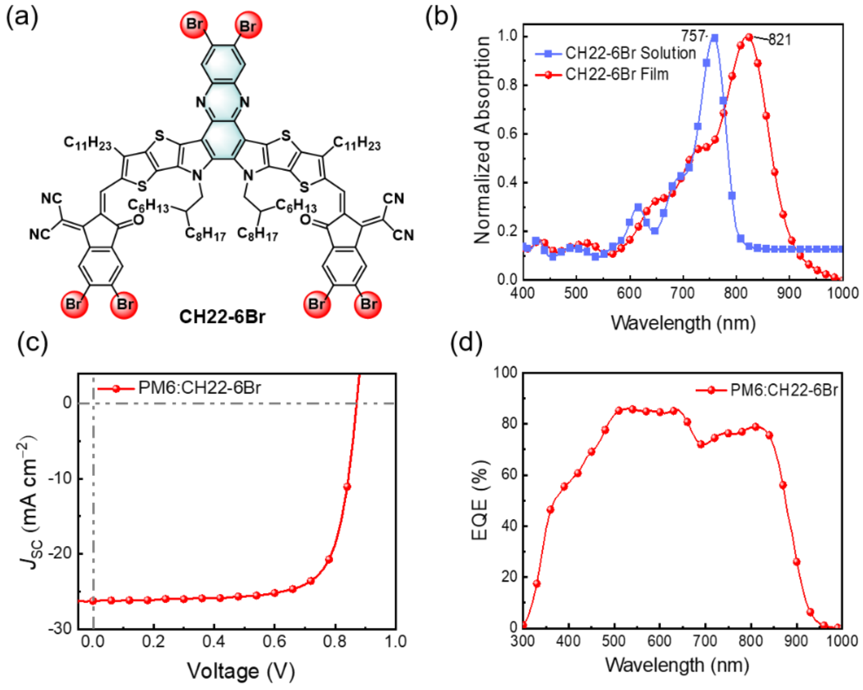

Supplementary Fig. 44 Molecular structures, UV-vis spectra of CH22-6Br and photovoltaic performances of the optimized OSCs. (a) Molecular structures of CH22-6Br. (b) The normalized absorption spectra of CH22-6Br in dilute chloroform solution and neat films. (c) Current density–voltage curves of PM6:CH22-6Br based OSCs. (d) The EQE spectra of PM6:CH22-6Br based OSCs.

Supplementary Table 12. Summary of device parameters for PM6:CH22-6Br and PM6:Y6 based OSCs.

Active layers	V_{OC} (V)	J_{SC} (mA cm^{-2})	Cal. J_{SC} (mA cm^{-2})	FF (%)	PCE (%)
PM6:CH22-6Br	0.871	26.25	25.34	74.31	17.00
PM6:Y6	0.852	25.90	25.16	74.54	16.45

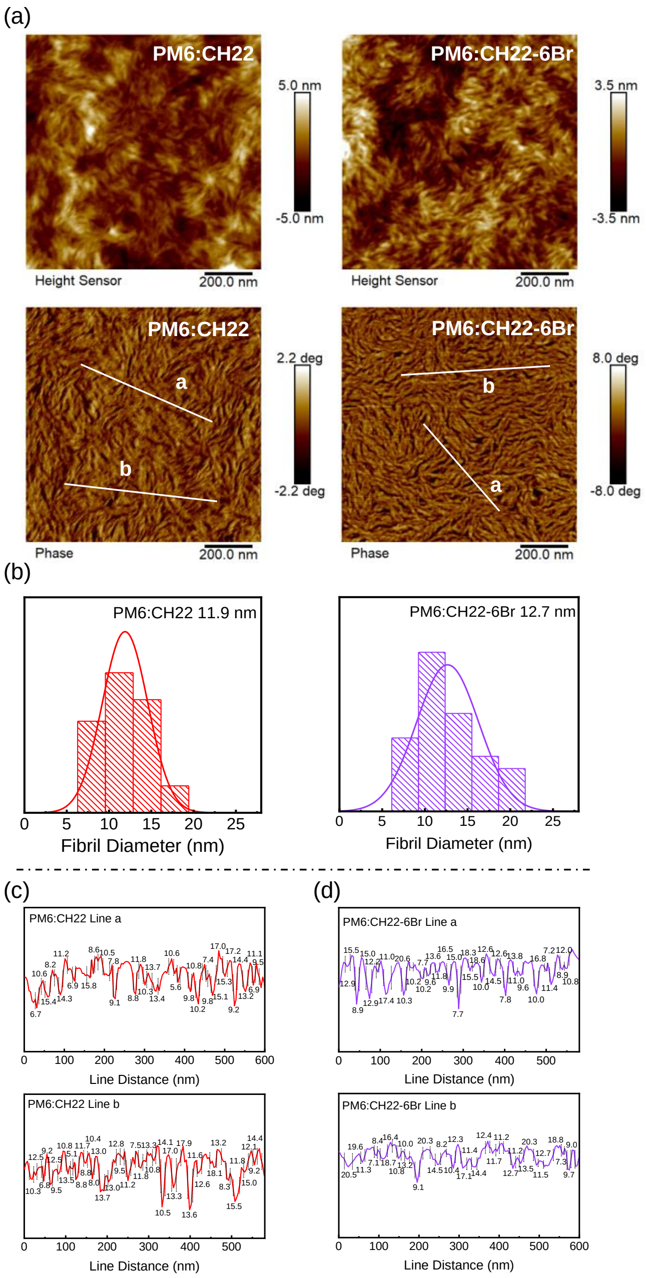

Supplementary Fig. 45 AFM characterization. (a) AFM height images and phase images of PM6:CH22 and PM6:CH22-6Br blended films. (b) The statistical distribution of the fibril diameter for PM6:CH22 and PM6: CH22-6Br blended films. (c, d) The line profile to obtain the fibril width form the AFM phase images of PM6:CH22 and PM6:CH22-6Br blended films.

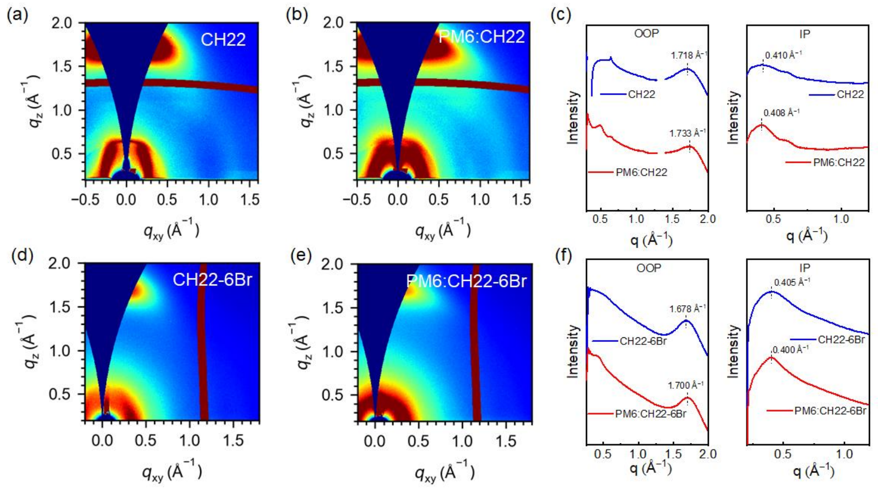

Supplementary Fig. 46 GIWAXS characterization. (a, b) 2D GIWAXS patterns of CH22-based neat and blended films. (c) The corresponding out of plane (OOP) and in plane (IP) extracted line-cut profiles of CH22-based neat and blended films. (d, e) 2D GIWAXS patterns of CH22-6Br-based neat and blended films. (f) The corresponding out of plane (OOP) and in plane (IP) extracted line-cut profiles of CH22-6Br-based neat and blended films.

Supplementary Table 13. The detailed parameters of corresponding 2D GIWAXS.

Materials	(010) Diffraction Peak				(100) Diffraction Peak	
	q (Å^{-1})	d[a] (Å)	FWHM (Å^{-1})	CCL[b] (Å)	q (Å^{-1})	d[a] (Å)
CH22	1.718	3.66	0.251	22.53	0.410	15.33
PM6:CH22	1.733	3.63	0.252	22.44	0.408	15.42
CH22-6Br	1.678	3.74	0.242	23.37	0.405	15.51
PM6:CH22-6Br	1.700	3.70	0.236	23.96	0.400	15.71

[a]Calculated from the equation: d-spacing=2π/q. [b]Obtained from the Scherrer equation: CCL= 2πK/FWHM, where FWHM is the full-width at half-maximum and K is a shape factor (K= 0.9 here).

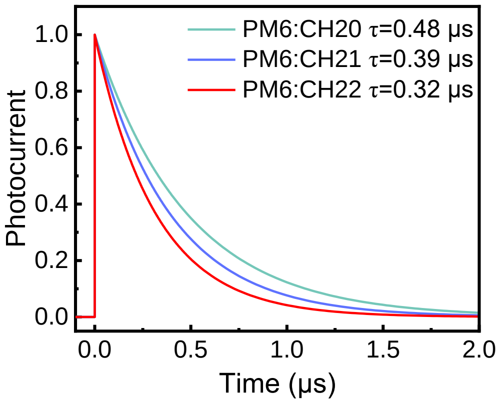

Supplementary Fig. 47 TPC. Transient photocurrent (TPC) measurements of the devices based on PM6:CH20, PM6:CH21 and PM6:CH22.

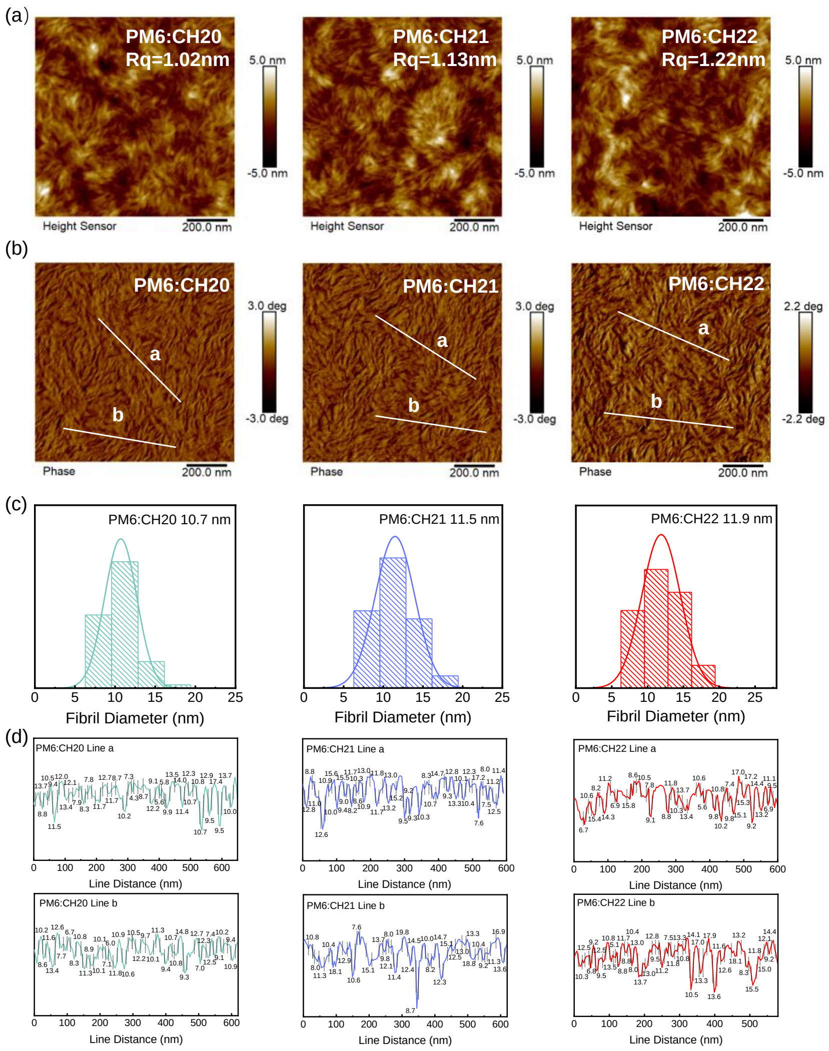

Supplementary Fig. 48 AFM characterization of the blended films. (a) AFM height and (b) phase images of PM6:CH20, PM6:CH21 and PM6:CH22 blended films. (c) The statistical distribution of fibril diameters existing in PM6:CH20, PM6:CH21 and PM6:CH22 blended films. (d) The line profile to obtain the fibril width form the AFM phase images of PM6:CH20, PM6:CH21 and PM6:CH22 blended films.

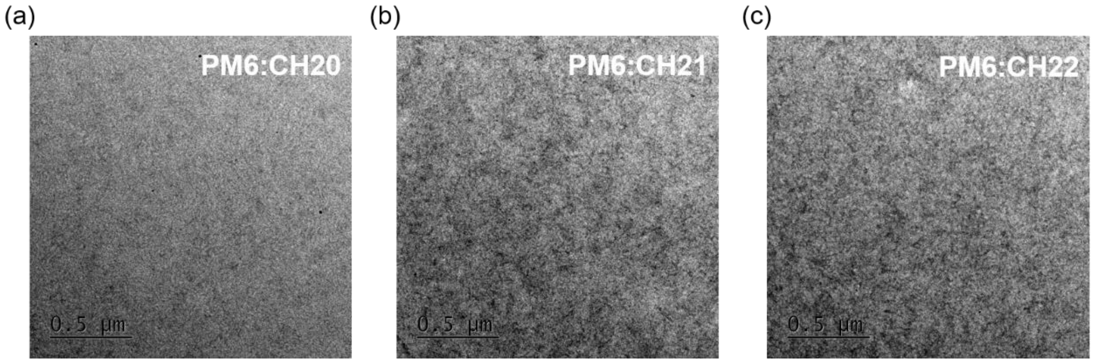

Supplementary Fig. 49 TEM characterization of the blended films. (a-c) TEM images of PM6:CH20, PM6:CH21 and PM6:CH22 blended films.

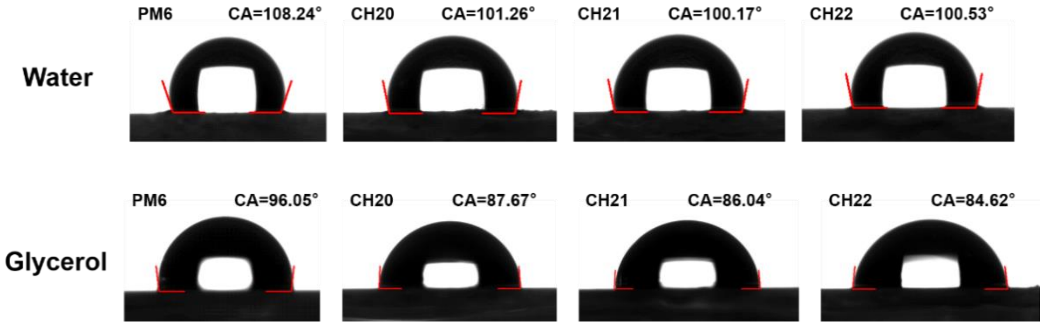

Supplementary Fig. 50 The images of water and glycerol drops on PM6, CH20, CH21 and CH22 neat films.

Supplementary Table 14. Information about surface energies of PM6, CH20, CH21, CH22 neat films calculated by water and glycerol contact angle.

Materials	θ_{water} (°)	$\theta_{glycerol}$ (°)	γ_d (mN m^{-1})	γ_p (mN m^{-1})	γ (mN m^{-1})	$\chi_{D:A}$ [a] (K)
PM6	108.24	96.05	0.60	16.07	16.67	–
CH20	101.26	87.67	0.79	21.91	22.70	0.46
CH21	100.17	86.04	0.80	23.00	23.80	0.63
CH22	100.53	84.62	1.02	23.40	24.42	0.74

[a]The molecular miscibility can be evaluated by Flory–Huggins interaction parameter χ, which is calculated by using the equation of: $\chi_{D:A} = K(\sqrt{\gamma_D} - \sqrt{\gamma_A})^2$.

Supplementary Table 15. The detailed parameters of corresponding 2D GIWAXS.

Materials	(010) Diffraction Peak				(100) Diffraction Peak			
	q (Å^{-1})	d[a] (Å)	FWHM (Å^{-1})	CCL[b] (Å)	q (Å^{-1})	d[a] (Å)	FWHM (Å^{-1})	CCL[b] (Å)
CH20	1.663	3.78	0.268	21.10	0.423	14.87	0.105	53.86
CH21	1.690	3.72	0.267	21.18	0.418	15.05	0.095	59.53
CH22	1.718	3.66	0.251	22.53	0.410	15.33	0.090	62.83
PM6:CH20	1.713	3.67	0.257	22.00	0.418	15.05	0.088	64.26
PM6:CH21	1.718	3.66	0.256	22.09	0.413	15.23	0.082	68.96
PM6:CH22	1.733	3.63	0.252	22.44	0.408	15.42	0.078	72.50

[a]Calculated from the equation: d-spacing=2π/q. [b]Obtained from the Scherrer equation: CCL= 2πK/FWHM, where FWHM is the full-width at half-maximum and K is a shape factor (K= 0.9 here).

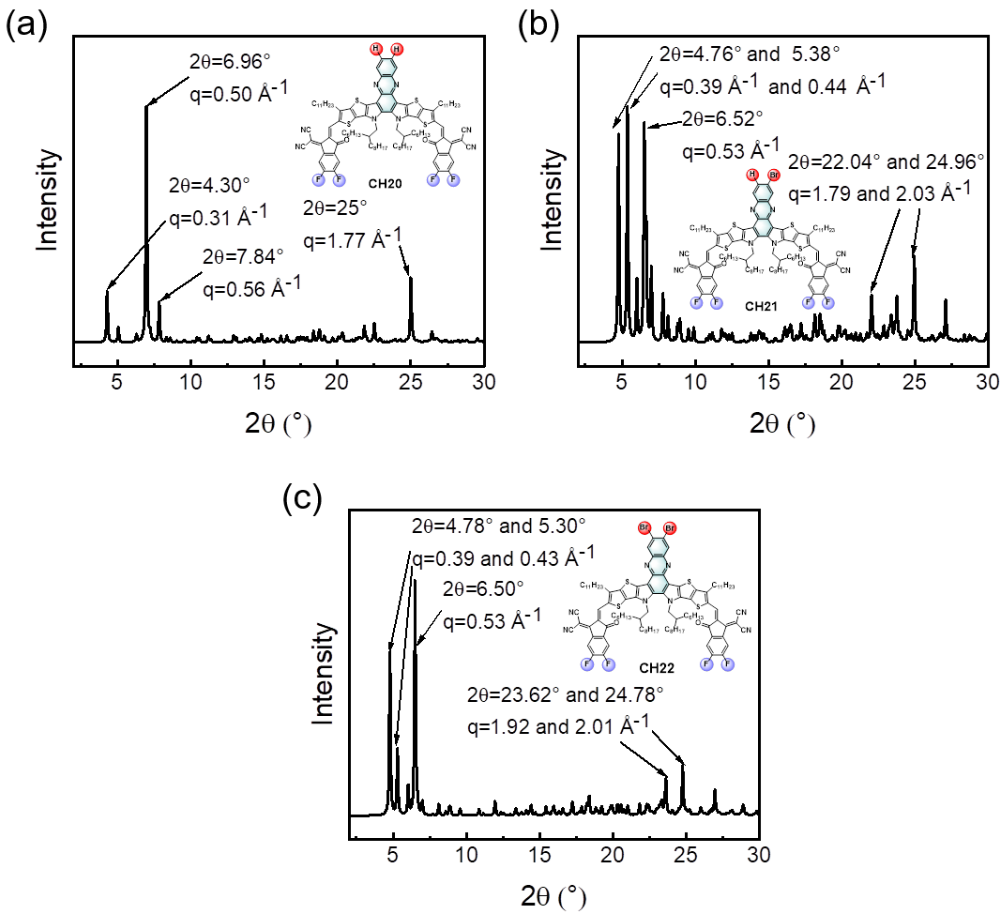

Supplementary Fig. 51 X-ray diffraction pattern generated with Mercury CSD software using single crystal diffraction data of (a) CH20, (b) CH21 and (c) CH22 respectively. According to the Bragg equation 2dsinθ =λ and d=2π/q, there are strong peaks of 0.31, 0.50, 0.56 and 1.77 Å^{-1} observed in X-ray diffraction pattern generated from single crystal structure data of CH20, corresponding to the packing distances of 20.52, 12.69, 11.26 and 3.56 Å, respectively. And there are strong peaks of 0.39, 0.44, 0.53, 1.79 and 2.03 Å^{-1} observed in X-ray diffraction pattern generated from single crystal structure data of CH21, corresponding to the packing distances of 16.13, 14.28, 11.78, 3.51 and 3.10 Å, respectively. And there are strong peaks of 0.39, 0.43, 0.53, 1.92 and 2.01 Å^{-1} observed in X-ray diffraction pattern generated from single crystal structure data of CH22, corresponding to the packing distances of 16.07, 14.49, 11.82, 3.27 and 3.12 Å, respectively.

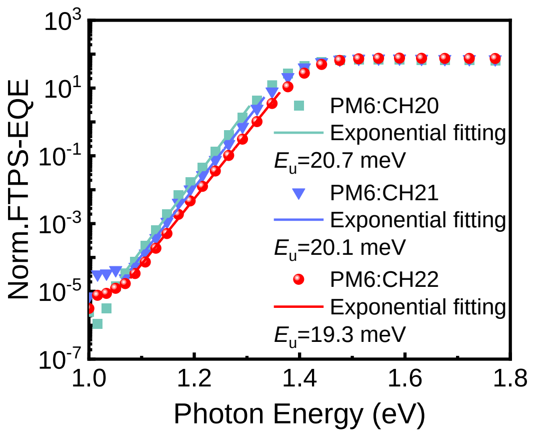

Supplementary Fig. 52 E_u of the PM6:CH20, PM6:CH21 and PM6:CH22-based devices.[13]

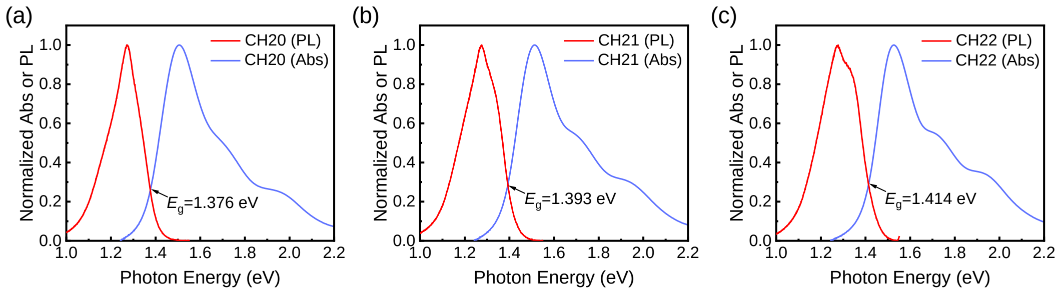

Supplementary Fig. 53 Details of optical E_g determination for (a) CH20, (b) CH21 and (c) CH22. E_g is estimated by the cross-point of normalized absorption and PL spectra[14] of the PM6:CH20, PM6:CH21 and PM6:CH22 blended films at 1.376, 1.393 and 1.414 eV, respectively.

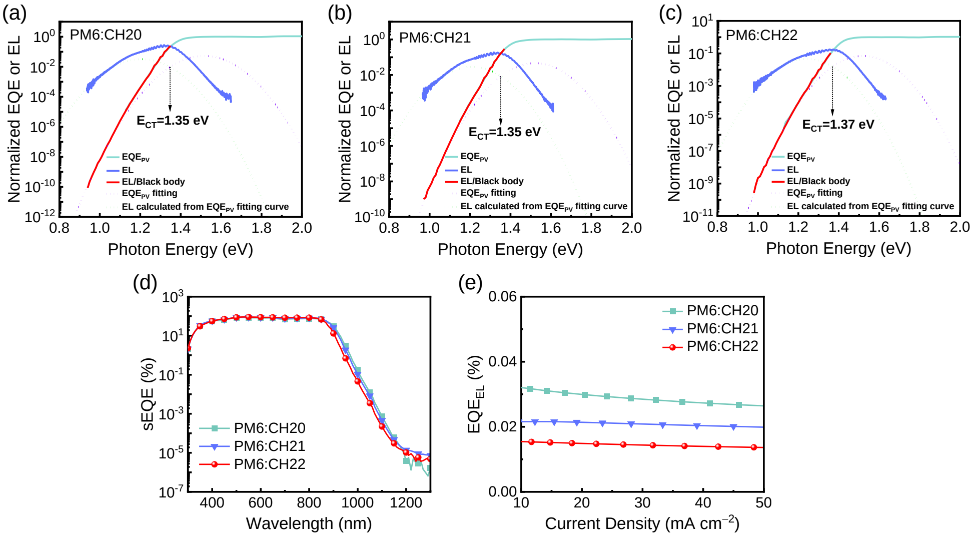

Supplementary Fig. 54 Energy loss analysis of the optimized OSCs. (a-c) Sensitive external quantum efficiency (sEQE) and Electroluminescence (EL) spectra for the optimized PM6:CH20, PM6:CH21 and PM6:CH22 blended films. (d) Sensitive external quantum efficiency (sEQE) spectra of the PM6:CH20, PM6:CH21 and PM6:CH22 based devices. (e) EQE_{EL} spectra for the PM6:CH20, PM6:CH21 and PM6:CH22 based devices.

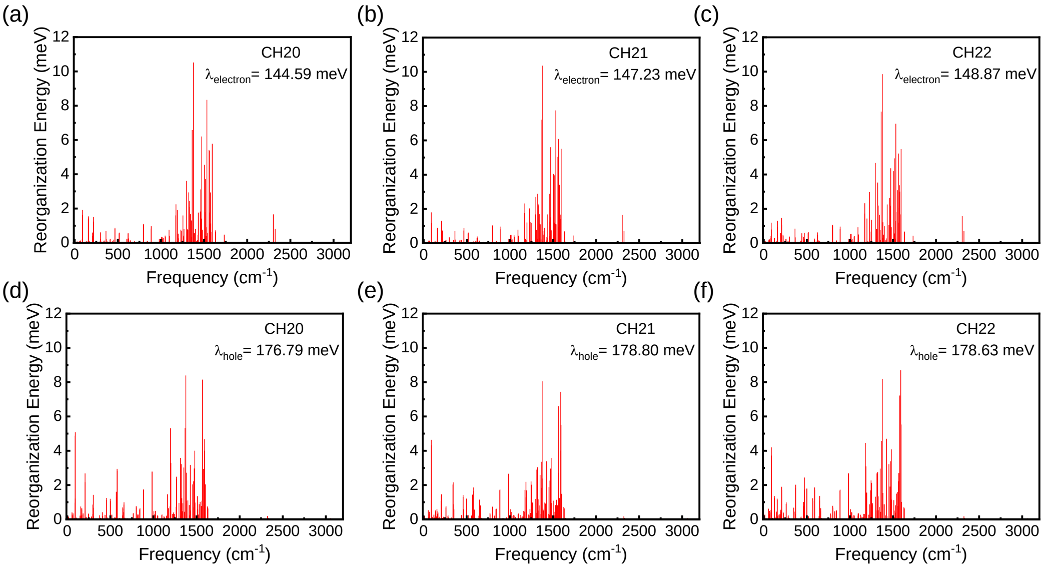

Supplementary Fig. 55 The calculated reorganization energies of (a, d) CH20, (b, e) CH21 and (c, f) CH22 at the level of ωB97XD/6-31G (d, p).[15]

Supplementary Table 16. Total energy loss values and different contributions in solar cells.

Active layer	E_g[a] (eV)	E_{CT}[b] (eV)	ΔE_{CT}[c] (eV)	$V_{oc,rad}$[d] (V)	$q\Delta V_r$[e] (eV)	V_{oc} (V)	$q\Delta V_{nr}$[f] (eV)	E_{loss} (eV)	$q\Delta V_{nr}'$[g] (eV)
PM6: CH20	1.376	1.35	0.026	1.089	0.261	0.881	0.208	0.495	0.211
PM6: CH21	1.393	1.35	0.043	1.094	0.256	0.872	0.222	0.525	0.220
PM6: CH22	1.414	1.37	0.044	1.112	0.258	0.884	0.228	0.530	0.230

[a]E_g is estimated via the intersection of normalized absorption and PL spectra. [b]E_{CT} is obtained by fitting the low-energy region of highly sensitive EQE and EL spectra. [c]ΔE_{CT}=E_g–E_{CT}. [d]$V_{OC,rad}$ is the radiative recombination limit for the V_{OC} of the solar cell, which can be determined by the equation: $V_{OC,rad} \approx \frac{kT_a}{q} ln\left(\frac{J_{ph}}{J_{0,rad}}\right)$, in which the $J_{0,rad}$ values are 1.603×10^{-17}, 1.388×10^{-17} and 6.848×10^{-18} for the CH20-, CH21- and CH22-based devices, calculated by the equation: $J_{0,rad} = q\int_0^{+\infty} EQE_{PV}(E)\phi_{BB}(E)dE$. [e] $q\Delta V_r = qE_{CT} - qV_{OC,rad}$. [f] $q\Delta V_{nr} = qV_{OC}^{rad} - qV_{OC}$. [g] $q\Delta V_{nr}'$ can be calculated by the equation: $q\Delta V_{nr}' = -qkTln\left(\frac{1}{EQE_{EL}}\right)$, in which the EQE_{EL} values are 2.94×10^{-4}, 2.10×10^{-4}, and 1.46×10^{-4} for the CH20-, CH21- and CH22-based devices, measured by EL measurements. The $q\Delta V_{nr}'$ values agreed well with the $q\Delta V_{nr}$ values.

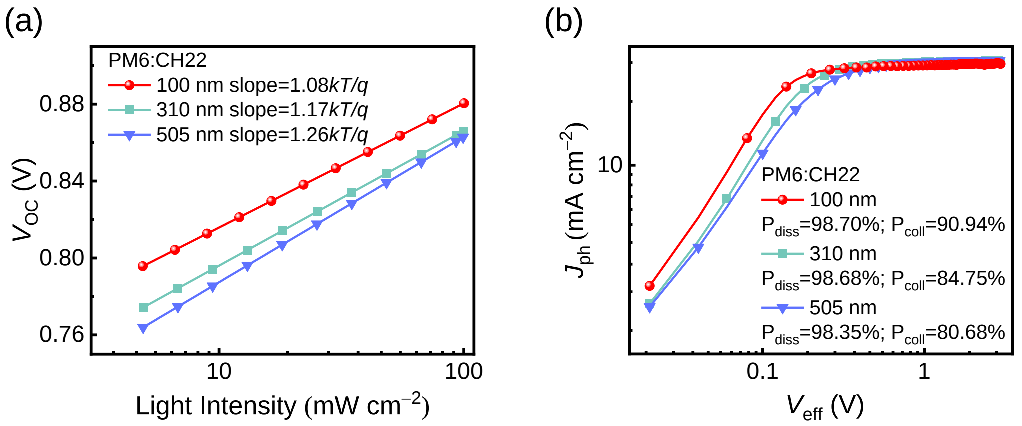

Supplementary Fig. 56 The thick-film device performance of PM6:CH22. (a) Dependences of V_{OC} on P_{light} of optimized OSCs of PM6:CH22 with different thicknesses (100 nm, 310 nm and 505 nm). (b) J_{ph} versus V_{eff} curves of optimized OSCs of PM6:CH22 with different thicknesses (100 nm, 310 nm and 505 nm).

Supplementary Table 17. Summary of device parameters for optimized PM6:CH22 OSCs with different thicknesses [a]

Active layers	V_{OC} (V)	J_{SC} (mA cm^{-2})	Cal. J_{SC} [b] (mA cm^{-2})	FF (%)	PCE (%)
100nm	0.884 (0.884±0.002)	26.74 (26.62±0.13)	26.17	80.62 (80.34±0.24)	19.06 (18.91±0.08)
220nm	0.870 (0.871±0.002)	27.44 (27.23±0.24)	26.58	73.00 (72.70±0.37)	17.44 (17.23±0.13)
310nm	0.873 (0.869±0.003)	27.76 (27.48±0.16)	26.97	70.10 (70.10±0.31)	16.99 (16.74±0.15)
405nm	0.869 (0.865±0.003)	27.93 (27.55±0.17)	27.09	66.93 (66.80±0.50)	16.25 (15.91±0.17)
505nm	0.867 (0.863±0.003)	28.14 (27.64±0.49)	27.40	64.37 (64.00±0.20)	15.70 (15.25±0.30)

[a]The average photovoltaic parameters calculated from 10 independent devices. [b]Current densities calculated from EQE curves.

Supplementary Table 18. The performance of the reported thick-film OSCs with ~500 nm.

Thickness (nm)	Active layer	V_{OC} (V)	J_{SC} (mA cm^{-2})	FF (%)	PCE (%)	Ref.
450nm	PBDB-T:a-IT-2OM	0.90	18.55	53.81	8.81	36
460nm	PM6:Y6	0.823	24.17	56.91	11.32	37
470nm	PM6:IDIC-C5Ph	0.921	20.15	70.12	13.01	38
480nm	PM6:Y6:F1	0.834	27.08	67.43	15.23	37
480nm	Si25:Y14	0.782	27.16	71.87	15.26	39
500nm	PM6:BTP-eC9:L8-BO-F	0.835	27.49	66.4	15.21	40
500nm	PT2:TTPTTT-4F:IDIC	0.86	22.0	61.4	11.6	41
500nm	PM7:MF2	0.953	19.20	54.90	10.04	42
500nm	PBDB-T:IDT-OB	0.84	17.23	47.9	6.93	43
505nm	PTQ10:IDTPC	0.91	17.81	56.9	9.2	44
505nm	PM6:CH22	0.867	28.14	64.37	15.70	This work
508nm	PTIQ4TFBT:PC71BM	0.79	17.80	57.9	8.13	45
510nm	PM7:MF1	0.929	16.97	63.9	10.07	42
519nm	PM6:F-2Cl	0.884	20.60	63	11.41	46
520nm	P2FEhp:Y6	0.70	26.10	56.90	10.50	47
520nm	PTzBI-Si:N2200	0.84	16.4	63.2	8.7	47
530nm	PM6:IDTN	0.912	17.93	52.00	8.5	48
540nm	PM6:EC9:L8-BO:BTP-S10	0.857	27.94	61.33	14.90	49
545nm	PM6:BTP-4Cl	0.841	28.2	58.0	13.8	50

Supplementary References

1. Becke AD. Density-functional thermochemistry. Iii. The role of exact exchange. *J. Chem. Phys.* **98**, 5648–5652 (1993).
2. Hariharan PC, Pople JA. Accuracy of AH_n equilibrium geometries by single determinant molecular orbital theory. *Mol. Phys.* **27**, 209–214 (1974).
3. Hay PJ, Wadt WR. Ab initio effective core potentials for molecular calculations. Potentials for k to au including the outermost core orbitals. *J. Chem. Phys.* **82**, (1985).
4. Frisch G, Robb J, Nakatsuji MC, Sonnenberg M, Farkas JBF, al. e. *Gaussian 16, Gaussian, Inc.: Wallingford, CT*, (2016).
5. Ji J, Zhu L, Xiong X, Liu F, Liang Z. Developing Y-branched polymer acceptor with 3d architecture to reconcile between crystallinity and miscibility yielding >15% efficient all-polymer solar cells. *Adv. Sci.* **9**, 2200864 (2022).
6. Zhu L, Zhang J, Guo Y, Yang C, Yi Y, Wei Z. Small exciton binding energies enabling direct charge photogeneration towards low-driving-force organic solar cells. *Angew. Chem. Int. Ed.* **60**, 15348–15353 (2021).
7. Li Y, Huang X, Sheriff HKM, Forrest SR. Semitransparent organic photovoltaics for building-integrated photovoltaic applications. *Nat. Rev. Mater.* **8**, 186–201 (2023).
8. Hughes MP, Rosenthal KD, Ran NA, Seifrid M, Bazan GC, Nguyen T-Q. Determining the dielectric constants of organic photovoltaic materials using impedance spectroscopy. *Adv. Funct. Mater.* **28**, 1801542 (2018).
9. Zhang X, *et al.* High fill factor organic solar cells with increased dielectric constant and molecular packing density. *Joule* **6**, 444–457 (2022).
10. Wang YM, *et al.* Optical gaps of organic solar cells as a reference for comparing voltage losses. *Adv. Energy Mater.* **8**, 1801352 (2018).
11. Cui Y, *et al.* Single-junction organic photovoltaic cells with approaching 18% efficiency. *Adv. Mater.* **32**, e1908205 (2020).
12. Wan X, Li C, Zhang M, Chen Y. Acceptor–Donor–Acceptor type molecules for high performance organic photovoltaics–chemistry and mechanism. *Chem. Soc. Rev.* **49**, 2828–2842 (2020).
13. Zhang Z, Li Y, Cai G, Zhang Y, Lu X, Lin Y. Selenium heterocyclic electron acceptor with small urbach energy for as-cast high-performance organic solar cells. *J. Am. Chem. Soc.* **142**, 18741–18745 (2020).
14. Wang Y, *et al.* Optical gaps of organic solar cells as a reference for comparing voltage losses. *Adv. Energy Mater.* **8**, 1801352 (2018).
15. Shi Y, *et al.* Small reorganization energy acceptors enable low energy losses in non-fullerene organic solar cells. *Nat. Commun.* **13**, 3256 (2022).
16. Yang F, Li C, Lai W, Zhang A, Huang H, Li W. Halogenated conjugated molecules for ambipolar field-effect transistors and non-fullerene organic solar cells. *Mater. Chem. Front.* **1**, 1389–1395 (2017).
17. Wang Y, *et al.* A halogenation strategy for over 12% efficiency nonfullerene organic solar cells. *Adv. Energy Mater.* **8**, 1702870 (2018).
18. Lu S, *et al.* Halogenation on terminal groups of itic based electron acceptors as an effective strategy for efficient polymer solar cells. *Sol. Energy* **195**, 429–435 (2020).

19. Qu J, *et al.* Bromination of the small-molecule acceptor with fixed position for high-performance solar cells. *Chem. Mater.* **31**, 8044–8051 (2019).
20. Luo Z, *et al.* Significantly improving the performance of polymer solar cells by the isomeric ending-group based small molecular acceptors: Insight into the isomerization. *Nano Energy* **66**, 104146 (2019).
21. Qin R, *et al.* Tuning terminal aromatics of electron acceptors to achieve high-efficiency organic solar cells. *J. Mater. Chem. A.* **7**, 27632–27639 (2019).
22. Zhang C, *et al.* Tetrabromination versus tetrachlorination: A molecular terminal engineering of nonfluorinated acceptors to control aggregation for highly efficient polymer solar cells with increased voc and higher jsc simultaneously. *Sol. RRL* **4**, 2000212 (2020).
23. Wan S-S, *et al.* A bromine and chlorine concurrently functionalized end group for benzo[1,2-b:4,5-b′]diselenophene-based non-fluorinated acceptors: A new hybrid strategy to balance the crystallinity and miscibility of blend films for enabling highly efficient polymer solar cells. *J. Mater. Chem. A.* **8**, 4856-4867 (2020).
24. Zhang M, *et al.* Effects of monohalogenated terminal units of non-fullerene acceptors on molecular aggregation and photovoltaic performance. *Sol. Energy* **208**, 866–872 (2020).
25. Wang H, *et al.* Bromination: An alternative strategy for non-fullerene small molecule acceptors. *Adv. Sci.* **7**, 1903784 (2020).
26. Luo Z, *et al.* Altering the positions of chlorine and bromine substitution on the end group enables high-performance acceptor and efficient organic solar cells. *Adv. Energy Mater.* **10**, 2002649 (2020).
27. Zhang L, Tu S, Wang W, Ling Q. Brominated small-molecule acceptors with a simple non-fused framework for efficient organic solar cells. *ACS Appl. Energ. Mater.* **4**, 4805–4814 (2021).
28. Mo D, Chen H, Zhu Y, Huang H-H, Chao P, He F. Effects of halogenated end groups on the performance of nonfullerene acceptors. *Acs Appl. Mater. Interfaces* **13**, 6147–6155 (2021).
29. Chen L, *et al.* End-group modifications with bromine and methyl in nonfullerene acceptors: The effect of isomerism. *Acs Appl. Mater. Interfaces* **13**, 29737–29745 (2021).
30. Liu Y, *et al.* Effects of brominated terminal groups on the performance of fused-ring electron acceptors in organic solar cells. *Dyes. Pigments* **194**, 109652 (2021).
31. Radford CL, Mudiyanselage PD, Stevens AL, Kelly TL. Heteroatoms as rotational blocking groups for non-fullerene acceptors in indoor organic solar cells. *ACS Energy Lett.* **7**, 1635–1641 (2022).
32. Liu X, *et al.* Near-infrared nonfullerene acceptors with halogenated terminated fused tris(thienothiophene) for efficient polymer solar cells. *Sol. Energy* **231**, 433–439 (2022).
33. Huang Y, *et al.* Tandem organic solar cells with 18.67% efficiency via careful subcell design and selection. *J. Mater. Chem. A.* **10**, 11238–11245 (2022).
34. Wang L, *et al.* Non-fullerene acceptors with hetero-dihalogenated terminals induce significant difference in single crystallography and enable binary organic solar cells with 17.5% efficiency. *Energy Environ. Sci.* **15**, 320–333 (2022).
35. He C, *et al.* Manipulating the D:A interfacial energetics and intermolecular packing for 19.2% efficiency organic photovoltaics. *Energy Environ. Sci.* **15**, 2537–2544 (2022).
36. Li M, Zhou Y, Zhang J, Song J, Bo Z. Tuning the dipole moments of nonfullerene acceptors with an asymmetric terminal strategy for highly efficient organic solar cells. *J. Mater. Chem. A.*

7, 8889–8896 (2019).
37. Zhao F, *et al.* Fullerene-liquid-crystal-induced micrometer-scale charge-carrier diffusion in organic bulk heterojunction. *Adv. Mater.* **35**, 2210463 (2023).
38. Li Y, *et al.* Subtle side chain triggers unexpected two-channel charge transport property enabling 80% fill factors and efficient thick-film organic photovoltaics. *The Innovation* **2**, 100090 (2021).
39. Pan F, *et al.* Binary non-fullerene-based polymer solar cells with a 430 nm thick active layer showing 15.39% efficiency and 73.38% fill factor. *J. Mater. Chem. A.* **9**, 7129−7136 (2021).
40. Cai Y, *et al.* Vertically optimized phase separation with improved exciton diffusion enables efficient organic solar cells with thick active layers. *Nat. Commun.* **13**, 2369 (2022).
41. Weng K, *et al.* High-efficiency organic solar cells with wide toleration of active layer thickness. *Sol. RRL* **4**, 2000476 (2020).
42. Gao W, *et al.* Thick-film organic solar cells achieving over 11% efficiency and nearly 70% fill factor at thickness over 400 nm. *Adv. Funct. Mater.* **30**, 1908336 (2020).
43. Feng S, *et al.* Fused-ring acceptors with asymmetric side chains for high-performance thick-film organic solar cells. *Adv. Mater.* **29**, 1703527 (2017).
44. Sun C, *et al.* A low cost and high performance polymer donor material for polymer solar cells. *Nat. Commun.* **9**, 743 (2018).
45. Li D, *et al.* A thieno[3,2-c]isoquinolin-5(4h)-one building block for efficient thick-film solar cells. *Adv. Energy Mater.* **8**, 1800397 (2018).
46. Zhang Y, *et al.* High performance thick-film nonfullerene organic solar cells with efficiency over 10% and active layer thickness of 600 nm. *Adv. Energy Mater.* **9**, 1902688 (2019).
47. Li Z-Y, *et al.* Achieving efficient thick film all-polymer solar cells using a green solvent additive. *Chin. J. Polym. Sci.* **38**, 323–331 (2020).
48. Li S, *et al.* Design of a new small-molecule electron acceptor enables efficient polymer solar cells with high fill factor. *Adv. Mater.* **29**, 1704051 (2017).
49. Zhan L, *et al.* Multiphase morphology with enhanced carrier lifetime via quaternary strategy enables high-efficiency, thick-film, and large-area organic photovoltaics. *Adv. Mater.* **34**, 2206269 (2022).
50. Ma L, *et al.* High-efficiency nonfullerene organic solar cells enabled by 1000 nm thick active layers with a low trap-state density. *Acs Appl. Mater. Interfaces* **12**, 18777−18784 (2020).
