# Supplementary material for: A rare case of brominated small molecule acceptors for high-efficiency organic solar cells
Source: Nat Commun. 2023 Aug 5;14:4707. doi: 10.1038/s41467-023-40423-6 (PMC10404295; doi:10.1038/s41467-023-40423-6)
Supplement: Supplementary file 3 — Description of Additional Supplementary Files [file 41467_2023_40423_MOESM3_ESM.pdf]

File name: Supplementary Data 1

Description: A ChemDraw file containing the experimental processes about the synthesis of all new compounds CH20, CH21 and CH22.

File name: Supplementary Data 2

Description: A checked PDF file using IUCr's CheckCIF routine about the structure factors and structural output of CH20 single crystal.

File name: Supplementary Data 3

Description: A checked PDF file using IUCr's CheckCIF routine about the structure factors and structural output of CH21 single crystal.

File name: Supplementary Data 4

Description: A checked PDF file using IUCr's CheckCIF routine about the structure factors and structural output of CH22 single crystal.
