# Supplementary material for: A rare case of brominated small molecule acceptors for high-efficiency organic solar cells
Source: Nat Commun. 2023 Aug 5;14:4707. doi: 10.1038/s41467-023-40423-6 (PMC10404295; doi:10.1038/s41467-023-40423-6)

checkCIF/PLATON report

Structure factors have been supplied for datablock(s) a

THIS REPORT IS FOR GUIDANCE ONLY. IF USED AS PART OF A REVIEW PROCEDURE FOR PUBLICATION, IT SHOULD NOT REPLACE THE EXPERTISE OF AN EXPERIENCED CRYSTALLOGRAPHIC REFEREE.

No syntax errors found. CIF dictionary Interpreting this report

Datablock: a

```
Bond precision:   C-C = 0.0141 A          Wavelength=1.54178

Cell:           a=13.2412(10)      b=17.7848(10)      c=21.2417(10)
                alpha=93.403(4)    beta=103.507(5)    gamma=99.261(5)
Temperature:    193 K
```

	Calculated	Reported
Volume	4775.9(5)	4775.9(5)
Space group	P -1	P -1
Hall group	-P 1	-P 1
Moiety formula	C104 H122 F4 N8 O2 S4 [+ solvent]	C104 H122 F4 N8 O2 S4
Sum formula	C104 H122 F4 N8 O2 S4 [+ solvent]	C104 H122 F4 N8 O2 S4
Mr	1720.34	1720.33
Dx,g cm-3	1.196	1.196
Z	2	2
Mu (mm-1)	1.394	1.394
F000	1836.0	1836.0
F000'	1843.45	
h,k,lmax	15,21,25	15,21,25
Nref	17498	17068
Tmin,Tmax	0.834,0.870	0.504,0.754
Tmin'	0.834	

```
Correction method= # Reported T Limits: Tmin=0.504 Tmax=0.754
AbsCorr = MULTI-SCAN

Data completeness= 0.975            Theta(max)= 68.242
```

R(reflections)= 0.1330(8332)

wR2(reflections)= 0.3544(17068)

S = 1.120

Npar= 1105

The following ALERTS were generated. Each ALERT has the format
test-name_ALERT_alert-type_alert-level.
Click on the hyperlinks for more details of the test.

Alert level B

PLAT220_ALERT_2_B NonSolvent Resd 1 C Ueq(max)/Ueq(min) Range 7.6 Ratio

Author Response: This Level B alert results from the presence of highly flexible carbon chains. This gives rise to larger values of Ueq for the carbon atoms in the structure.

PLAT340_ALERT_3_B Low Bond Precision on C-C Bonds .... 0.01408 Ang.

Author Response: The crystal does not diffract well at high angles, which lead to this B level alert.

Alert level C

```
PLAT026_ALERT_3_C Ratio Observed / Unique Reflections (too) Low ..        49% Check
PLAT029_ALERT_3_C _diffrn_measured_fraction_theta_full value Low .      0.976 Why?
PLAT082_ALERT_2_C High R1 Value ................................        0.13 Report
PLAT084_ALERT_3_C High wR2 Value (i.e. > 0.25) ..................       0.35 Report
PLAT222_ALERT_3_C NonSolvent Resd 1  H   Uiso(max)/Uiso(min) Range      7.3 Ratio
PLAT230_ALERT_2_C Hirshfeld Test Diff for    C63      --C64      .      5.9 s.u.
PLAT230_ALERT_2_C Hirshfeld Test Diff for    C65      --C66      .      5.2 s.u.
PLAT230_ALERT_2_C Hirshfeld Test Diff for    C81      --C82      .      5.9 s.u.
PLAT234_ALERT_4_C Large Hirshfeld Difference C56      --C57      .     0.22 Ang.
PLAT234_ALERT_4_C Large Hirshfeld Difference C67      --C68      .     0.22 Ang.
PLAT234_ALERT_4_C Large Hirshfeld Difference C70      --C71      .     0.16 Ang.
PLAT234_ALERT_4_C Large Hirshfeld Difference C71      --C72      .     0.20 Ang.
PLAT234_ALERT_4_C Large Hirshfeld Difference C73      --C74      .     0.20 Ang.
PLAT234_ALERT_4_C Large Hirshfeld Difference C82      --C83      .     0.18 Ang.
PLAT234_ALERT_4_C Large Hirshfeld Difference C87      --C88      .     0.20 Ang.
PLAT234_ALERT_4_C Large Hirshfeld Difference C96      --C97      .     0.17 Ang.
PLAT234_ALERT_4_C Large Hirshfeld Difference C99      --C100     .     0.23 Ang.
PLAT234_ALERT_4_C Large Hirshfeld Difference C100     --C101     .     0.21 Ang.
PLAT234_ALERT_4_C Large Hirshfeld Difference C101     --C102     .     0.19 Ang.
PLAT234_ALERT_4_C Large Hirshfeld Difference C102     --C103     .     0.22 Ang.
PLAT241_ALERT_2_C High   'MainMol' Ueq as Compared to Neighbors of      C72 Check
PLAT260_ALERT_2_C Large Average Ueq of Residue Including        S1    0.142 Check
PLAT410_ALERT_2_C Short Intra H...H Contact  H62B     ..H89B     .     1.91 Ang.
                                                  x,y,z  =      1_555 Check
PLAT411_ALERT_2_C Short Inter H...H Contact  H10D     ..H87A     .     2.04 Ang.
                                               -x,-y,-z  =      2_555 Check
PLAT411_ALERT_2_C Short Inter H...H Contact  H10D     ..H87B     .     2.05 Ang.
```

```
                                                -x,-y,-z  =      2_555 Check
PLAT411_ALERT_2_C Short Inter H...H Contact  H60A     ..H97A     .       2.05 Ang.
                                               1-x,-y,-z  =      2_655 Check
PLAT413_ALERT_2_C Short Inter XH3 .. XHn     H10I     ..H69A     .       2.10 Ang.
                                             1-x,1-y,1-z  =      2_666 Check
PLAT413_ALERT_2_C Short Inter XH3 .. XHn     H10K     ..H69A     .       2.08 Ang.
                                             1-x,1-y,1-z  =      2_666 Check
PLAT413_ALERT_2_C Short Inter XH3 .. XHn     H54A     ..H88C     .       2.14 Ang.
                                               1+x,y,1+z  =      1_656 Check
PLAT413_ALERT_2_C Short Inter XH3 .. XHn     H61B     ..H76A     .       2.07 Ang.
                                               2-x,-y,1-z  =     2_756 Check
PLAT413_ALERT_2_C Short Inter XH3 .. XHn     H61C     ..H77C     .       2.12 Ang.
                                               2-x,-y,1-z  =     2_756 Check
PLAT911_ALERT_3_C Missing FCF Refl Between Thmin & STh/L=    0.600        408 Report
PLAT913_ALERT_3_C Missing # of Very Strong Reflections in FCF ....          4 Note
PLAT977_ALERT_2_C Check Negative Difference Density on H61B      .      -0.31 eA-3
```

Alert level G

```
PLAT002_ALERT_2_G Number of Distance or Angle Restraints on AtSite         56 Note
PLAT003_ALERT_2_G Number of Uiso or Uij Restrained non-H Atoms ...         55 Report
PLAT083_ALERT_2_G SHELXL Second Parameter in WGHT  Unusually Large      15.00 Why ?
PLAT153_ALERT_1_G The s.u.'s on the Cell Axes   are Equal ..(Note)      0.001 Ang.
PLAT172_ALERT_4_G The CIF-Embedded .res File Contains DFIX Records         10 Report
PLAT173_ALERT_4_G The CIF-Embedded .res File Contains DANG Records         44 Report
PLAT178_ALERT_4_G The CIF-Embedded .res File Contains SIMU Records          6 Report
PLAT333_ALERT_2_G Large Aver C6-Ring C-C Dist C20      -C31      .       1.43 Ang.
PLAT343_ALERT_2_G Unusual sp3      Angle Range in Main Residue for        C87 Check
PLAT432_ALERT_2_G Short Inter X...Y Contact  C61      ..C77      .       2.98 Ang.
                                               2-x,-y,1-z  =     2_756 Check
PLAT432_ALERT_2_G Short Inter X...Y Contact  C61      ..C76      .       3.18 Ang.
                                               2-x,-y,1-z  =     2_756 Check
PLAT432_ALERT_2_G Short Inter X...Y Contact  C87      ..C101     .       3.05 Ang.
                                                -x,-y,-z  =      2_555 Check
PLAT605_ALERT_4_G Largest Solvent Accessible VOID in the Structure        129 A**3
PLAT793_ALERT_4_G Model has Chirality at C63         (Centro SPGR)          R Verify
PLAT793_ALERT_4_G Model has Chirality at C90         (Centro SPGR)          S Verify
PLAT860_ALERT_3_G Number of Least-Squares Restraints ............         426 Note
PLAT910_ALERT_3_G Missing # of FCF Reflection(s) Below Theta(Min).          1 Note
PLAT912_ALERT_4_G Missing # of FCF Reflections Above STh/L=  0.600         21 Note
PLAT941_ALERT_3_G Average HKL Measurement Multiplicity ..........         2.8 Low
PLAT978_ALERT_2_G Number C-C Bonds with Positive Residual Density.          0 Info
PLAT992_ALERT_5_G Repd & Actual _reflns_number_gt Values Differ by          9 Check
```

---

0 **ALERT level A** = Most likely a serious problem - resolve or explain
2 **ALERT level B** = A potentially serious problem, consider carefully
34 **ALERT level C** = Check. Ensure it is not caused by an omission or oversight
21 **ALERT level G** = General information/check it is not something unexpected

1 ALERT type 1 CIF construction/syntax error, inconsistent or missing data
26 ALERT type 2 Indicator that the structure model may be wrong or deficient
10 ALERT type 3 Indicator that the structure quality may be low
19 ALERT type 4 Improvement, methodology, query or suggestion
1 ALERT type 5 Informative message, check

It is advisable to attempt to resolve as many as possible of the alerts in all categories. Often the minor alerts point to easily fixed oversights, errors and omissions in your CIF or refinement strategy, so attention to these fine details can be worthwhile. In order to resolve some of the more serious problems it may be necessary to carry out additional measurements or structure refinements. However, the purpose of your study may justify the reported deviations and the more serious of these should normally be commented upon in the discussion or experimental section of a paper or in the "special_details" fields of the CIF. checkCIF was carefully designed to identify outliers and unusual parameters, but every test has its limitations and alerts that are not important in a particular case may appear. Conversely, the absence of alerts does not guarantee there are no aspects of the results needing attention. It is up to the individual to critically assess their own results and, if necessary, seek expert advice.

Publication of your CIF in IUCr journals

A basic structural check has been run on your CIF. These basic checks will be run on all CIFs submitted for publication in IUCr journals (*Acta Crystallographica*, *Journal of Applied Crystallography*, *Journal of Synchrotron Radiation*); however, if you intend to submit to *Acta Crystallographica Section C* or *E* or *IUCrData*, you should make sure that full publication checks are run on the final version of your CIF prior to submission.

Publication of your CIF in other journals

Please refer to the *Notes for Authors* of the relevant journal for any special instructions relating to CIF submission.

PLATON version of 18/05/2022; check.def file version of 17/05/2022

Datablock a - ellipsoid plot

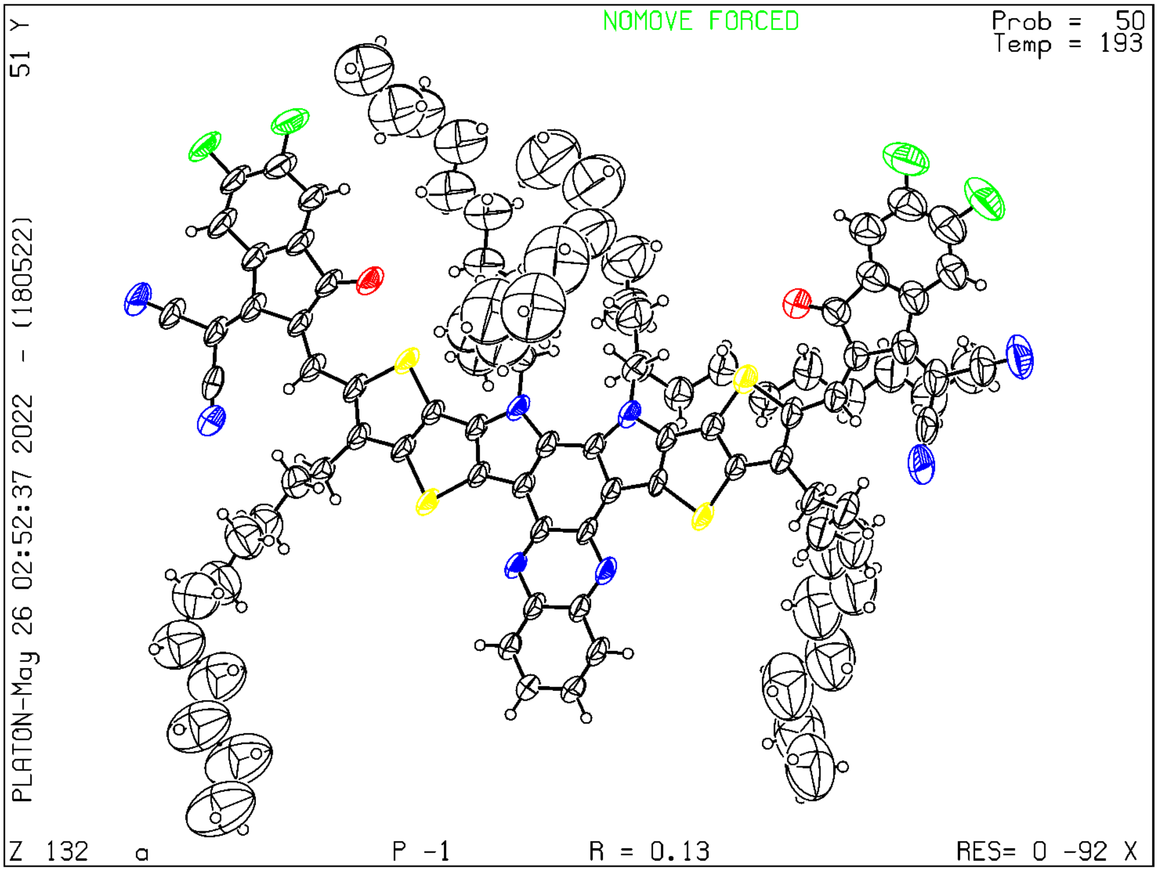

Supplement: Supplementary file 5 — Supplementary Data 2 [file 41467_2023_40423_MOESM5_ESM.pdf]
