# Supplementary material for: A rare case of brominated small molecule acceptors for high-efficiency organic solar cells
Source: Nat Commun. 2023 Aug 5;14:4707. doi: 10.1038/s41467-023-40423-6 (PMC10404295; doi:10.1038/s41467-023-40423-6)
Supplement: Supplementary file 8 — Solar Cells Reporting Summary [file 41467_2023_40423_MOESM8_ESM.pdf]

Corresponding author(s): Yongsheng Chen

Solar Cells Reporting Summary

Nature Research wishes to improve the reproducibility of the work that we publish. This form is intended for publication with all accepted papers reporting the characterization of photovoltaic devices and provides structure for consistency and transparency in reporting. Some list items might not apply to an individual manuscript, but all fields must be completed for clarity.

For further information on Nature Research policies, including our data availability policy, see Authors & Referees.

Experimental design

Please check: are the following details reported in the manuscript?

1. Dimensions

Item	Yes/No	Details
Area of the tested solar cells	[x] Yes [ ] No	2 x 2 mm. This information can be found in the "Methods, Device fabrication" section of the text.
Method used to determine the device area	[x] Yes [ ] No	Area of the tested solar cells is 4.1 mm^2 defined by an optical profilometer. This information can be found in the "Methods, Device fabrication" section of the text.

2. Current-voltage characterization

Item	Yes/No	Details
Current density-voltage (J-V) plots in both forward and backward direction	[ ] Yes [x] No	Current-voltage characteristics plotted only in forward direction, which can be found in "Fig. 5a" of the text. The hysteresis effect is negligible in organic photovoltaic cells.
Voltage scan conditions<br>*For instance: scan direction, speed, dwell times*	[x] Yes [ ] No	Forward direction, the current-voltage scan speed and dwell time were 0.02 V/s and 1 ms respectively, respectively. This information can be found in the "Methods, Characterization of the OSCs" section of the text.
Test environment<br>*For instance: characterization temperature, in air or in glove box*	[x] Yes [ ] No	Our devices were characterized at room temperature (ca. 25 Celsius degree) in N2 glove box. This information can be found in the "Methods, Characterization of the OSCs" section of the text.
Protocol for preconditioning of the device before its characterization	[x] Yes [ ] No	No preconditioning was applied. This information can be found in the "Methods, Characterization of the OSCs" section of the text.
Stability of the J-V characteristic<br>*Verified with time evolution of the maximum power point or with the photocurrent at maximum power point; see ref. 7 for details.*	[ ] Yes [x] No	Organic photovoltaic devices show no decay or instability during the test of J-V characteristics.

3. Hysteresis or any other unusual behaviour

Item	Yes/No	Details
Description of the unusual behaviour observed during the characterization	[x] Yes [ ] No	No hysteresis or other unusual behavior was observed during the characterization of organic solar cells. Generally, organic photovoltaic devices do not have hysteresis problems. This information can be found in the "Methods, Characterization of the OSCs" section of the text.
Related experimental data	[ ] Yes [x] No	No hysteresis or other unusual behavior was observed during the characterization of organic solar cells. Generally, organic photovoltaic devices do not have hysteresis problems.

4. Efficiency

Item	Yes/No	Details
External quantum efficiency (EQE) or incident photons to current efficiency (IPCE)	[x] Yes [ ] No	The EQE spectra were measured by using a QE-R Solar Cell Spectral Response Measurement System (Enli Technology Co., Ltd.) equipped with a standard Si diode in air condition. This information can be found in the "Fig. 5b" of the text.
A comparison between the integrated response under the standard reference spectrum and the response measure under the simulator	[x] Yes [ ] No	The measurement of EQE curves and integrated current densities of devices under the standard reference spectrum are shown in "Fig. 5b and Table 2". The current densities from J-V measurement under the simulator are shown in "Table 2", which are all consistent with the values obtained from the EQE measurements and are within <3% error. This information can be found in the "Results, Photovoltaic performances" section of the text.
For tandem solar cells, the bias illumination and bias voltage used for each subcell	[ ] Yes [x] No	In this work, no tandem device has been studied.

5. Calibration

Light source and reference cell or sensor used for the characterization	☒ Yes ☐ No	The J–V measurements were performed by using the solar simulator (SS-F5-3A, Enli Technology, xenon lamp, filter model AMFG2.0) along with AM 1.5G spectra (100 mW cm^-2), Which was calibrated by a standard Si solar cell (made by Enli Technology Co., Ltd., and calibrated report can be traced to NREL. This information can be found in the "Methods, Characterization of the OSCs" section of the text.
Confirmation that the reference cell was calibrated and certified	☒ Yes ☐ No	The reference cell was calibrated and certified. This information can be found in the "Methods, Characterization of the OSCs" section of the text.
Calculation of spectral mismatch between the reference cell and the devices under test	☒ Yes ☐ No	Less than 3%. This information can be found in the "Methods, Characterization of the OSCs" section of the text.

6. Mask/aperture

Size of the mask/aperture used during testing	☒ Yes ☐ No	1.6 x 1.6 mm. This information can be found in the "Methods, Device fabrication" section of the text.
Variation of the measured short-circuit current density with the mask/aperture area	☐ Yes ☒ No	In this work, the variation of the measured J-V with mask/aperture area has not been studied.

7. Performance certification

Identity of the independent certification laboratory that confirmed the photovoltaic performance	☐ Yes ☒ No	We didn't certify the performance of the devices in this work. The main reason for this is that during this compared study, we have used a control device of Y6 following the state of the art work for comparison and quality control (Supplementary Table 5), which gave quite consistent results with widely reported literatures (such as Nat. Energy 6, 605-613 (2021)). Also, the PCE we had is not the highest yet in the whole field.
A copy of any certificate(s) *Provide in Supplementary Information*	☐ Yes ☒ No	No certification.

8. Statistics

Number of solar cells tested	☒ Yes ☐ No	The average parameters were calculated from 15 independent devices. This information can be found in the "Results, Photovoltaic performances" section and "Table 2".
Statistical analysis of the device performance	☒ Yes ☐ No	Statistical average and error bars of a standard deviation from the average values of PCEs are listed in "Table 2 and Figure 5a".

9. Long-term stability analysis

Type of analysis, bias conditions and environmental conditions *For instance: illumination type, temperature, atmosphere humidity, encapsulation method, preconditioning temperature*	☒ Yes ☐ No	PCEs of PM6:CH22-based OSCs could be maintained above 96% and ~85% compared to its initial PCEs after 1500 h under room temperature and 400 h under heat treatment at 65 Celsius degree (Supplementary Fig. 15), respectively. This information can be found in the "Results, Photovoltaic performances" section and "Methods, Characterization of the OSCs" section of the text.
