# Supplementary material for: A rare case of brominated small molecule acceptors for high-efficiency organic solar cells
Source: Nat Commun. 2023 Aug 5;14:4707. doi: 10.1038/s41467-023-40423-6 (PMC10404295; doi:10.1038/s41467-023-40423-6)

checkCIF/PLATON report

Structure factors have been supplied for datablock(s) a

THIS REPORT IS FOR GUIDANCE ONLY. IF USED AS PART OF A REVIEW PROCEDURE FOR PUBLICATION, IT SHOULD NOT REPLACE THE EXPERTISE OF AN EXPERIENCED CRYSTALLOGRAPHIC REFEREE.

No syntax errors found. CIF dictionary Interpreting this report

Datablock: a

Bond precision: C-C = 0.0161 A Wavelength=1.34139

Cell:	a=15.5364(10)	b=17.0821(13)	c=19.3025(14)
	alpha=97.034(4)	beta=102.772(4)	gamma=102.220(4)
Temperature:	193 K		

	Calculated	Reported
Volume	4805.2(6)	4805.2(6)
Space group	P -1	P -1
Hall group	-P 1	-P 1
Moiety formula	C104 H121 Br F4 N8 O2 S4	C104 H121 Br F4 N8 O2 S4
Sum formula	C104 H121 Br F4 N8 O2 S4	C104 H121 Br F4 N8 O2 S4
Mr	1799.24	1799.23
Dx,g cm-3	1.243	1.244
Z	2	2
Mu (mm-1)	1.231	1.231
F000	1904.0	1904.0
F000'	1908.06	
h,k,lmax	19,21,24	19,21,24
Nref	20097	19555
Tmin,Tmax	0.863,0.884	0.497,0.751
Tmin'	0.863	

Correction method= # Reported T Limits: Tmin=0.497 Tmax=0.751
AbsCorr = MULTI-SCAN

Data completeness= 0.973 Theta(max)= 57.606

R(reflections)= 0.1498(8958) wR2(reflections)= 0.3810(19555)

S = 1.067 Npar= 1122

The following ALERTS were generated. Each ALERT has the format
test-name_ALERT_alert-type_alert-level.
Click on the hyperlinks for more details of the test.

Alert level B

```
PLAT084_ALERT_3_B High wR2 Value (i.e. > 0.25) ...................       0.38 Report
```

Author Response: Due to poor crystal quality and very weak diffraction.

```
PLAT341_ALERT_3_B Low Bond Precision on  C-C Bonds ...............    0.01608 Ang.
```

Author Response: Quality of the data is not enough to get good bond length parameters.

Alert level C

```
PLAT026_ALERT_3_C Ratio Observed / Unique Reflections (too) Low ..        46% Check
PLAT082_ALERT_2_C High R1 Value ..................................       0.15 Report
PLAT220_ALERT_2_C NonSolvent   Resd 1  C   Ueq(max)/Ueq(min) Range        5.9 Ratio
PLAT222_ALERT_3_C NonSolvent Resd 1  H   Uiso(max)/Uiso(min) Range        5.9 Ratio
PLAT230_ALERT_2_C Hirshfeld Test Diff for    N3      --C25      .         5.2 s.u.
PLAT230_ALERT_2_C Hirshfeld Test Diff for    C65     --C70      .         5.7 s.u.
PLAT230_ALERT_2_C Hirshfeld Test Diff for    C74     --C75      .         5.1 s.u.
PLAT234_ALERT_4_C Large Hirshfeld Difference N3      --C5       .        0.17 Ang.
PLAT234_ALERT_4_C Large Hirshfeld Difference N6      --C51      .        0.17 Ang.
PLAT234_ALERT_4_C Large Hirshfeld Difference N7      --C54      .        0.20 Ang.
PLAT234_ALERT_4_C Large Hirshfeld Difference C48     --C50      .        0.17 Ang.
PLAT234_ALERT_4_C Large Hirshfeld Difference C52     --C66      .        0.16 Ang.
PLAT234_ALERT_4_C Large Hirshfeld Difference C57     --C68      .        0.18 Ang.
PLAT234_ALERT_4_C Large Hirshfeld Difference C59     --C64      .        0.18 Ang.
PLAT234_ALERT_4_C Large Hirshfeld Difference C63     --C77      .        0.16 Ang.
PLAT234_ALERT_4_C Large Hirshfeld Difference C64     --C67      .        0.17 Ang.
PLAT234_ALERT_4_C Large Hirshfeld Difference C65     --C69      .        0.18 Ang.
PLAT234_ALERT_4_C Large Hirshfeld Difference C66     --C76      .        0.17 Ang.
PLAT234_ALERT_4_C Large Hirshfeld Difference C67     --C72      .        0.22 Ang.
PLAT234_ALERT_4_C Large Hirshfeld Difference C70     --C73      .        0.19 Ang.
PLAT234_ALERT_4_C Large Hirshfeld Difference C71     --C85      .        0.16 Ang.
PLAT234_ALERT_4_C Large Hirshfeld Difference C72     --C94      .        0.19 Ang.
PLAT234_ALERT_4_C Large Hirshfeld Difference C73     --C84      .        0.16 Ang.
PLAT234_ALERT_4_C Large Hirshfeld Difference C77     --C78      .        0.16 Ang.
PLAT234_ALERT_4_C Large Hirshfeld Difference C78     --C82      .        0.17 Ang.
PLAT234_ALERT_4_C Large Hirshfeld Difference C79     --C83      .        0.17 Ang.
PLAT234_ALERT_4_C Large Hirshfeld Difference C85     --C86      .        0.18 Ang.
PLAT234_ALERT_4_C Large Hirshfeld Difference C94     --C96      .        0.16 Ang.
PLAT241_ALERT_2_C High   'MainMol' Ueq as Compared to Neighbors of        C43 Check
PLAT242_ALERT_2_C Low    'MainMol' Ueq as Compared to Neighbors of        C18 Check
PLAT242_ALERT_2_C Low    'MainMol' Ueq as Compared to Neighbors of        C60 Check
PLAT242_ALERT_2_C Low    'MainMol' Ueq as Compared to Neighbors of        C61 Check
PLAT242_ALERT_2_C Low    'MainMol' Ueq as Compared to Neighbors of        C65 Check
PLAT260_ALERT_2_C Large Average Ueq of Residue Including      Br1      0.147 Check
PLAT360_ALERT_2_C Short  C(sp3)-C(sp3) Bond  C68     - C74      .        1.36 Ang.
PLAT361_ALERT_2_C Long   C(sp3)-C(sp3) Bond  C25     - C65      .        1.68 Ang.
```

```
PLAT410_ALERT_2_C Short Intra H...H Contact  H68A     ..H74A     .       1.94 Ang.
                                                x,y,z  =     1_555 Check
PLAT410_ALERT_2_C Short Intra H...H Contact  H70A     ..H73A     .       1.99 Ang.
                                                x,y,z  =     1_555 Check
PLAT410_ALERT_2_C Short Intra H...H Contact  H70B     ..H73A     .       1.95 Ang.
                                                x,y,z  =     1_555 Check
PLAT410_ALERT_2_C Short Intra H...H Contact  H86B     ..H89A     .       1.95 Ang.
                                                x,y,z  =     1_555 Check
PLAT411_ALERT_2_C Short Inter H...H Contact  H10D     ..H88B     .       2.09 Ang.
                                            -x,1-y,-z  =     2_565 Check
PLAT411_ALERT_2_C Short Inter H...H Contact  H10J     ..H97A     .       2.06 Ang.
                                          -1+x,y,-1+z  =     1_454 Check
PLAT411_ALERT_2_C Short Inter H...H Contact  H70B     ..H86A     .       2.08 Ang.
                                          1-x,2-y,1-z  =     2_676 Check
PLAT411_ALERT_2_C Short Inter H...H Contact  H72B     ..H76B     .       2.03 Ang.
                                              1+x,y,z  =     1_655 Check
PLAT411_ALERT_2_C Short Inter H...H Contact  H75A     ..H97A     .       2.09 Ang.
                                          -1+x,y,-1+z  =     1_454 Check
PLAT411_ALERT_2_C Short Inter H...H Contact  H75A     ..H99B     .       2.03 Ang.
                                          -1+x,y,-1+z  =     1_454 Check
PLAT411_ALERT_2_C Short Inter H...H Contact  H75B     ..H99B     .       2.08 Ang.
                                          -1+x,y,-1+z  =     1_454 Check
PLAT411_ALERT_2_C Short Inter H...H Contact  H96A     ..H96A     .       2.05 Ang.
                                          3-x,1-y,2-z  =     2_867 Check
PLAT413_ALERT_2_C Short Inter XH3 .. XHn     H10B     ..H90B     .       2.08 Ang.
                                            -x,1-y,-z  =     2_565 Check
PLAT413_ALERT_2_C Short Inter XH3 .. XHn     H10H     ..H88A     .       2.05 Ang.
                                            -x,1-y,-z  =     2_565 Check
PLAT413_ALERT_2_C Short Inter XH3 .. XHn     H80B     ..H81C     .       2.10 Ang.
                                          1-x,1-y,2-z  =     2_667 Check
PLAT413_ALERT_2_C Short Inter XH3 .. XHn     H81B     ..H98B     .       2.09 Ang.
                                             -1+x,y,z  =     1_455 Check
PLAT601_ALERT_2_C Unit Cell Contains Solvent Accessible VOIDS of .         65 Ang**3
PLAT906_ALERT_3_C Large K Value in the Analysis of Variance ......     19.544 Check
PLAT906_ALERT_3_C Large K Value in the Analysis of Variance ......      3.581 Check
PLAT910_ALERT_3_C Missing # of FCF Reflection(s) Below Theta(Min).          6 Note
PLAT911_ALERT_3_C Missing FCF Refl Between Thmin & STh/L=    0.600        116 Report
PLAT913_ALERT_3_C Missing # of Very Strong Reflections in FCF ....          5 Note
PLAT918_ALERT_3_C Reflection(s) with I(obs) much Smaller I(calc) .          3 Check
PLAT977_ALERT_2_C Check Negative Difference Density on H81C      .      -0.34 eA-3
PLAT977_ALERT_2_C Check Negative Difference Density on H86B      .      -0.47 eA-3
PLAT977_ALERT_2_C Check Negative Difference Density on H93A      .      -0.33 eA-3
PLAT977_ALERT_2_C Check Negative Difference Density on H95B      .      -0.36 eA-3
```

Alert level G

```
ABSMU01_ALERT_1_G  Calculation of _exptl_absorpt_correction_mu
                not performed for this radiation type.
PLAT002_ALERT_2_G Number of Distance or Angle Restraints on AtSite         50 Note
PLAT003_ALERT_2_G Number of Uiso or Uij Restrained non-H Atoms ...         62 Report
PLAT083_ALERT_2_G SHELXL Second Parameter in WGHT  Unusually Large      30.00 Why ?
PLAT154_ALERT_1_G The s.u.'s on the Cell Angles are Equal ..(Note)      0.004 Degree
PLAT172_ALERT_4_G The CIF-Embedded .res File Contains DFIX Records          9 Report
PLAT173_ALERT_4_G The CIF-Embedded .res File Contains DANG Records         40 Report
PLAT178_ALERT_4_G The CIF-Embedded .res File Contains SIMU Records          6 Report
PLAT301_ALERT_3_G Main Residue  Disorder .............(Resd  1  )          1% Note
PLAT343_ALERT_2_G Unusual sp3      Angle Range in Main Residue for        C20 Check
```

```
PLAT343_ALERT_2_G Unusual          Angle Range in Main Residue for        C73 Check
PLAT343_ALERT_2_G Unusual sp3      Angle Range in Main Residue for       C104 Check
PLAT367_ALERT_2_G Long?  C(sp?)-C(sp?) Bond  C70      - C73      .       1.52 Ang.
PLAT367_ALERT_2_G Long?  C(sp?)-C(sp?) Bond  C73      - C84      .       1.52 Ang.
PLAT432_ALERT_2_G Short Inter X...Y Contact  C75      ..C97      .       3.15 Ang.
                                         -1+x,y,-1+z  =      1_454 Check
PLAT432_ALERT_2_G Short Inter X...Y Contact  C81      ..C98      .       3.16 Ang.
                                            -1+x,y,z  =      1_455 Check
PLAT432_ALERT_2_G Short Inter X...Y Contact  C88      ..C103     .       3.00 Ang.
                                           -x,1-y,-z  =      2_565 Check
PLAT432_ALERT_2_G Short Inter X...Y Contact  C97      ..C104     .       3.14 Ang.
                                           1+x,y,1+z  =      1_656 Check
PLAT432_ALERT_2_G Short Inter X...Y Contact  C98      ..C104     .       3.17 Ang.
                                           1+x,y,1+z  =      1_656 Check
PLAT434_ALERT_2_G Short Inter HL..HL Contact F1       ..F4       .       2.79 Ang.
                                         1-x,2-y,2-z  =      2_677 Check
PLAT793_ALERT_4_G Model has Chirality at C23        (Centro SPGR)          R Verify
PLAT793_ALERT_4_G Model has Chirality at C65        (Centro SPGR)          R Verify
PLAT860_ALERT_3_G Number of Least-Squares Restraints .............        475 Note
PLAT912_ALERT_4_G Missing # of FCF Reflections Above STh/L=  0.600        421 Note
PLAT933_ALERT_2_G Number of HKL-OMIT Records in Embedded .res File         18 Note
PLAT941_ALERT_3_G Average HKL Measurement Multiplicity ...........        2.5 Low
PLAT978_ALERT_2_G Number C-C Bonds with Positive Residual Density.          0 Info
PLAT992_ALERT_5_G Repd & Actual _reflns_number_gt Values Differ by          2 Check
```

```
  0 ALERT level A = Most likely a serious problem - resolve or explain
  2 ALERT level B = A potentially serious problem, consider carefully
 63 ALERT level C = Check. Ensure it is not caused by an omission or oversight
 28 ALERT level G = General information/check it is not something unexpected

  2 ALERT type 1 CIF construction/syntax error, inconsistent or missing data
 50 ALERT type 2 Indicator that the structure model may be wrong or deficient
 13 ALERT type 3 Indicator that the structure quality may be low
 27 ALERT type 4 Improvement, methodology, query or suggestion
  1 ALERT type 5 Informative message, check
```

It is advisable to attempt to resolve as many as possible of the alerts in all categories. Often the minor alerts point to easily fixed oversights, errors and omissions in your CIF or refinement strategy, so attention to these fine details can be worthwhile. In order to resolve some of the more serious problems it may be necessary to carry out additional measurements or structure refinements. However, the purpose of your study may justify the reported deviations and the more serious of these should normally be commented upon in the discussion or experimental section of a paper or in the "special_details" fields of the CIF. checkCIF was carefully designed to identify outliers and unusual parameters, but every test has its limitations and alerts that are not important in a particular case may appear. Conversely, the absence of alerts does not guarantee there are no aspects of the results needing attention. It is up to the individual to critically assess their own results and, if necessary, seek expert advice.

Publication of your CIF in IUCr journals

A basic structural check has been run on your CIF. These basic checks will be run on all CIFs submitted for publication in IUCr journals (*Acta Crystallographica*, *Journal of Applied Crystallography*, *Journal of Synchrotron Radiation*); however, if you intend to submit to *Acta Crystallographica Section C* or *E* or *IUCrData*, you should make sure that full publication checks are run on the final version of your CIF prior to submission.

Publication of your CIF in other journals

Please refer to the *Notes for Authors* of the relevant journal for any special instructions relating to CIF submission.

PLATON version of 19/02/2022; check.def file version of 19/02/2022

Datablock a - ellipsoid plot

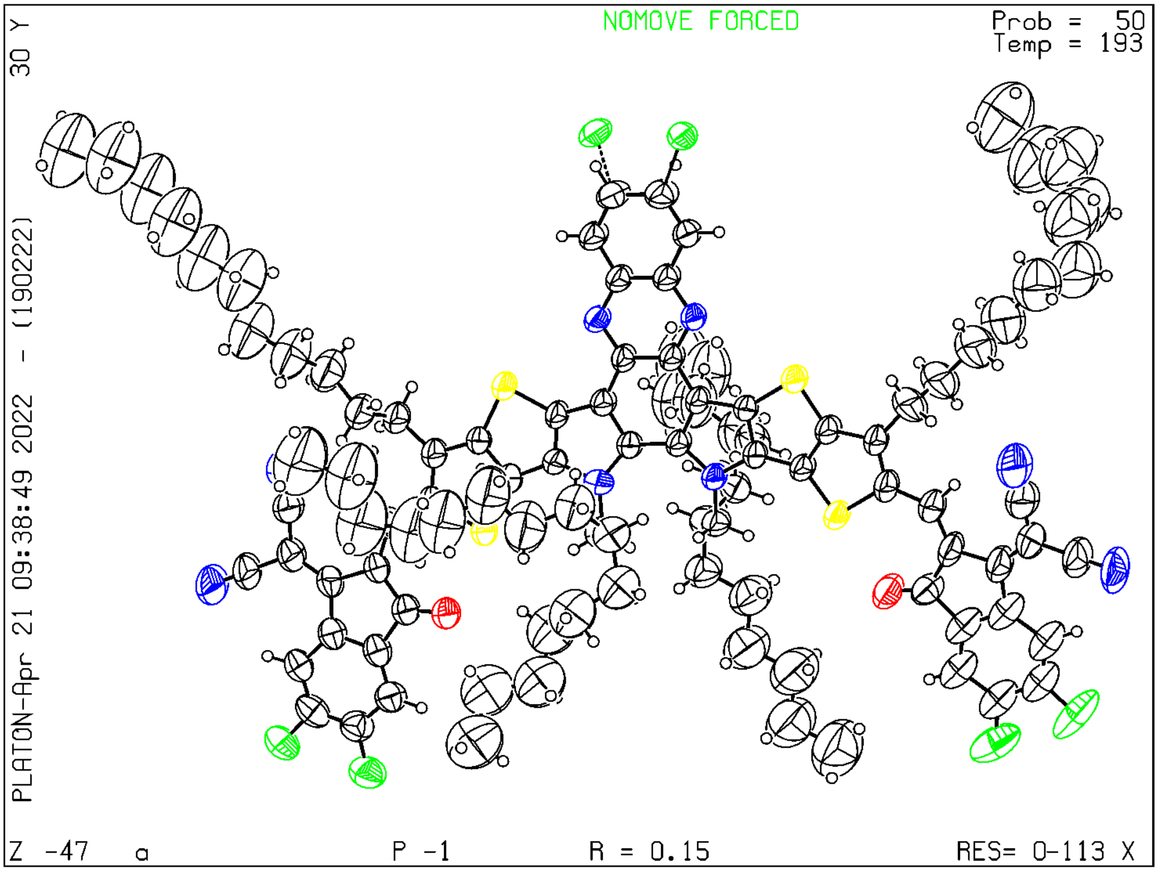

Supplement: Supplementary file 6 — Supplementary Data 3 [file 41467_2023_40423_MOESM6_ESM.pdf]
