# Supplementary material for: A rare case of brominated small molecule acceptors for high-efficiency organic solar cells
Source: Nat Commun. 2023 Aug 5;14:4707. doi: 10.1038/s41467-023-40423-6 (PMC10404295; doi:10.1038/s41467-023-40423-6)

checkCIF/PLATON report

Structure factors have been supplied for datablock(s) a

THIS REPORT IS FOR GUIDANCE ONLY. IF USED AS PART OF A REVIEW PROCEDURE FOR PUBLICATION, IT SHOULD NOT REPLACE THE EXPERTISE OF AN EXPERIENCED CRYSTALLOGRAPHIC REFEREE.

No syntax errors found. CIF dictionary Interpreting this report

Datablock: a

Bond precision: C-C = 0.0147 A   Wavelength=1.34139

Cell:	a=15.4802(4)	b=17.2861(5)	c=19.2225(5)
	alpha=96.771(2)	beta=102.930(1)	gamma=101.974(1)
Temperature:	193 K		

	Calculated	Reported
Volume	4830.9(2)	4830.9(2)
Space group	P -1	P -1
Hall group	-P 1	-P 1
Moiety formula	C104 H120 Br2 F4 N8 O2 S4	C104 H120 Br2 F4 N8 O2 S4
Sum formula	C104 H120 Br2 F4 N8 O2 S4	C104 H120 Br2 F4 N8 O2 S4
Mr	1878.13	1878.13
Dx,g cm-3	1.291	1.291
Z	2	2
Mu (mm-1)	1.549	1.549
F000	1972.0	1972.0
F000'	1974.18	
h,k,lmax	19,21,24	19,21,24
Nref	19868	19703
Tmin,Tmax	0.818,0.857	0.625,0.751
Tmin'	0.818	

Correction method= # Reported T Limits: Tmin=0.625 Tmax=0.751
AbsCorr = MULTI-SCAN

Data completeness= 0.992   Theta(max)= 57.137

R(reflections)= 0.1283(9434)   wR2(reflections)= 0.3405(19703)

S = 1.086   Npar= 1211

The following ALERTS were generated. Each ALERT has the format

test-name_ALERT_alert-type_alert-level.

Click on the hyperlinks for more details of the test.

Alert level B

```
PLAT230_ALERT_2_B Hirshfeld Test Diff for    C64      --C65      .       16.4 s.u.
```

Author Response: This Level B alert results from the presence of flexible carbon chain, which gives rise to larger values of Ueq.

Alert level C

```
PLAT026_ALERT_3_C Ratio Observed / Unique Reflections (too) Low ..        48% Check
PLAT082_ALERT_2_C High R1 Value .................................       0.13 Report
PLAT084_ALERT_3_C High wR2 Value (i.e. > 0.25) ..................       0.34 Report
PLAT220_ALERT_2_C NonSolvent   Resd 1  C   Ueq(max)/Ueq(min) Range       5.4 Ratio
PLAT220_ALERT_2_C NonSolvent   Resd 1  N   Ueq(max)/Ueq(min) Range       3.2 Ratio
PLAT222_ALERT_3_C NonSolvent Resd 1  H   Uiso(max)/Uiso(min) Range       5.7 Ratio
PLAT234_ALERT_4_C Large Hirshfeld Difference C57      --C58      .       0.16 Ang.
PLAT234_ALERT_4_C Large Hirshfeld Difference C59      --C60      .       0.16 Ang.
PLAT234_ALERT_4_C Large Hirshfeld Difference C85      --C86      .       0.18 Ang.
PLAT234_ALERT_4_C Large Hirshfeld Difference C93      --C94      .       0.18 Ang.
PLAT241_ALERT_2_C High   'MainMol' Ueq as Compared to Neighbors of        C65 Check
PLAT242_ALERT_2_C Low    'MainMol' Ueq as Compared to Neighbors of        C30 Check
PLAT242_ALERT_2_C Low    'MainMol' Ueq as Compared to Neighbors of        C64 Check
PLAT260_ALERT_2_C Large Average Ueq of Residue Including      Br1      0.122 Check
PLAT334_ALERT_2_C Small <C-C> Benzene Dist.   C1       -C6       .       1.37 Ang.
PLAT341_ALERT_3_C Low Bond Precision on  C-C Bonds ...............    0.01472 Ang.
PLAT360_ALERT_2_C Short  C(sp3)-C(sp3) Bond  C80      - C81      .       1.39 Ang.
PLAT361_ALERT_2_C Long   C(sp3)-C(sp3) Bond  C64      - C65      .       1.66 Ang.
PLAT411_ALERT_2_C Short Inter H...H Contact  H70B     ..H72B     .       2.03 Ang.
                                              1-x,-y,1-z  =      2_656 Check
PLAT412_ALERT_2_C Short Intra XH3 .. XHn     H76B     ..H79A     .       1.88 Ang.
                                                   x,y,z  =      1_555 Check
PLAT413_ALERT_2_C Short Inter XH3 .. XHn     H75A     ..H89C     .       2.06 Ang.
                                               1-x,-y,-z  =      2_655 Check
PLAT413_ALERT_2_C Short Inter XH3 .. XHn     H78A     ..H79B     .       2.04 Ang.
                                              2-x,-y,1-z  =      2_756 Check
PLAT413_ALERT_2_C Short Inter XH3 .. XHn     H78B     ..H79B     .       2.02 Ang.
                                              2-x,-y,1-z  =      2_756 Check
PLAT413_ALERT_2_C Short Inter XH3 .. XHn     H79B     ..H79B     .       2.14 Ang.
                                              2-x,-y,1-z  =      2_756 Check
PLAT431_ALERT_2_C Short Inter HL..A Contact  Br2      ..S3       .       3.52 Ang.
                                              -x,1-y,1-z  =      2_566 Check
PLAT906_ALERT_3_C Large K Value in the Analysis of Variance ......     15.372 Check
PLAT906_ALERT_3_C Large K Value in the Analysis of Variance ......      3.461 Check
PLAT911_ALERT_3_C Missing FCF Refl Between Thmin & STh/L=    0.600         41 Report
PLAT977_ALERT_2_C Check Negative Difference Density on H73A      .      -0.50 eA-3
PLAT977_ALERT_2_C Check Negative Difference Density on H73B      .      -0.39 eA-3
PLAT977_ALERT_2_C Check Negative Difference Density on H77A      .      -0.31 eA-3
```

Alert level G

```
ABSMU01_ALERT_1_G  Calculation of _exptl_absorpt_correction_mu
                not performed for this radiation type.
PLAT002_ALERT_2_G Number of Distance or Angle Restraints on AtSite         66 Note
PLAT003_ALERT_2_G Number of Uiso or Uij Restrained non-H Atoms ...         70 Report
PLAT083_ALERT_2_G SHELXL Second Parameter in WGHT  Unusually Large      30.00 Why ?
PLAT172_ALERT_4_G The CIF-Embedded .res File Contains DFIX Records          8 Report
PLAT173_ALERT_4_G The CIF-Embedded .res File Contains DANG Records         53 Report
PLAT178_ALERT_4_G The CIF-Embedded .res File Contains SIMU Records          8 Report
PLAT186_ALERT_4_G The CIF-Embedded .res File Contains ISOR Records          2 Report
PLAT188_ALERT_3_G A Non-default SIMU Restraint Value has been used     0.0030 Report
PLAT188_ALERT_3_G A Non-default SIMU Restraint Value has been used     0.0050 Report
PLAT188_ALERT_3_G A Non-default SIMU Restraint Value has been used     0.0050 Report
PLAT188_ALERT_3_G A Non-default SIMU Restraint Value has been used     0.0050 Report
PLAT188_ALERT_3_G A Non-default SIMU Restraint Value has been used     0.0050 Report
PLAT188_ALERT_3_G A Non-default SIMU Restraint Value has been used     0.0050 Report
PLAT188_ALERT_3_G A Non-default SIMU Restraint Value has been used     0.0050 Report
PLAT188_ALERT_3_G A Non-default SIMU Restraint Value has been used     0.0050 Report
PLAT300_ALERT_4_G Atom Site Occupancy of C1A        Constrained at        0.5 Check
PLAT300_ALERT_4_G Atom Site Occupancy of C97        Constrained at        0.5 Check
PLAT300_ALERT_4_G Atom Site Occupancy of C98        Constrained at        0.5 Check
PLAT300_ALERT_4_G Atom Site Occupancy of C99        Constrained at        0.5 Check
PLAT300_ALERT_4_G Atom Site Occupancy of C100       Constrained at        0.5 Check
PLAT300_ALERT_4_G Atom Site Occupancy of C101       Constrained at        0.5 Check
PLAT300_ALERT_4_G Atom Site Occupancy of C102       Constrained at        0.5 Check
PLAT300_ALERT_4_G Atom Site Occupancy of C103       Constrained at        0.5 Check
PLAT300_ALERT_4_G Atom Site Occupancy of C104       Constrained at        0.5 Check
PLAT300_ALERT_4_G Atom Site Occupancy of C105       Constrained at        0.5 Check
PLAT300_ALERT_4_G Atom Site Occupancy of C106       Constrained at        0.5 Check
PLAT300_ALERT_4_G Atom Site Occupancy of C107       Constrained at        0.5 Check
PLAT300_ALERT_4_G Atom Site Occupancy of C108       Constrained at        0.5 Check
PLAT300_ALERT_4_G Atom Site Occupancy of C109       Constrained at        0.5 Check
PLAT300_ALERT_4_G Atom Site Occupancy of C110       Constrained at        0.5 Check
PLAT300_ALERT_4_G Atom Site Occupancy of C111       Constrained at        0.5 Check
PLAT300_ALERT_4_G Atom Site Occupancy of C112       Constrained at        0.5 Check
PLAT300_ALERT_4_G Atom Site Occupancy of C113       Constrained at        0.5 Check
PLAT300_ALERT_4_G Atom Site Occupancy of C114       Constrained at        0.5 Check
PLAT300_ALERT_4_G Atom Site Occupancy of C115       Constrained at        0.5 Check
PLAT300_ALERT_4_G Atom Site Occupancy of H1AA       Constrained at        0.5 Check
PLAT300_ALERT_4_G Atom Site Occupancy of H1AB       Constrained at        0.5 Check
PLAT300_ALERT_4_G Atom Site Occupancy of H10A       Constrained at        0.5 Check
PLAT300_ALERT_4_G Atom Site Occupancy of H10B       Constrained at        0.5 Check
PLAT300_ALERT_4_G Atom Site Occupancy of H10C       Constrained at        0.5 Check
PLAT300_ALERT_4_G Atom Site Occupancy of H10D       Constrained at        0.5 Check
PLAT300_ALERT_4_G Atom Site Occupancy of H10E       Constrained at        0.5 Check
PLAT300_ALERT_4_G Atom Site Occupancy of H10F       Constrained at        0.5 Check
PLAT300_ALERT_4_G Atom Site Occupancy of H10G       Constrained at        0.5 Check
PLAT300_ALERT_4_G Atom Site Occupancy of H10H       Constrained at        0.5 Check
PLAT300_ALERT_4_G Atom Site Occupancy of H10I       Constrained at        0.5 Check
PLAT300_ALERT_4_G Atom Site Occupancy of H10J       Constrained at        0.5 Check
PLAT300_ALERT_4_G Atom Site Occupancy of H10K       Constrained at        0.5 Check
PLAT300_ALERT_4_G Atom Site Occupancy of H10L       Constrained at        0.5 Check
PLAT300_ALERT_4_G Atom Site Occupancy of H10M       Constrained at        0.5 Check
PLAT300_ALERT_4_G Atom Site Occupancy of H10N       Constrained at        0.5 Check
PLAT300_ALERT_4_G Atom Site Occupancy of H10O       Constrained at        0.5 Check
PLAT300_ALERT_4_G Atom Site Occupancy of H10P       Constrained at        0.5 Check
PLAT300_ALERT_4_G Atom Site Occupancy of H10Q       Constrained at        0.5 Check
```

```
PLAT300_ALERT_4_G Atom Site Occupancy of H10R       Constrained at        0.5 Check
PLAT300_ALERT_4_G Atom Site Occupancy of H10S       Constrained at        0.5 Check
PLAT300_ALERT_4_G Atom Site Occupancy of H10T       Constrained at        0.5 Check
PLAT300_ALERT_4_G Atom Site Occupancy of H10U       Constrained at        0.5 Check
PLAT300_ALERT_4_G Atom Site Occupancy of H11A       Constrained at        0.5 Check
PLAT300_ALERT_4_G Atom Site Occupancy of H11B       Constrained at        0.5 Check
PLAT300_ALERT_4_G Atom Site Occupancy of H11C       Constrained at        0.5 Check
PLAT300_ALERT_4_G Atom Site Occupancy of H11D       Constrained at        0.5 Check
PLAT300_ALERT_4_G Atom Site Occupancy of H11E       Constrained at        0.5 Check
PLAT300_ALERT_4_G Atom Site Occupancy of H11F       Constrained at        0.5 Check
PLAT300_ALERT_4_G Atom Site Occupancy of H11G       Constrained at        0.5 Check
PLAT300_ALERT_4_G Atom Site Occupancy of H11H       Constrained at        0.5 Check
PLAT300_ALERT_4_G Atom Site Occupancy of H11I       Constrained at        0.5 Check
PLAT300_ALERT_4_G Atom Site Occupancy of H11J       Constrained at        0.5 Check
PLAT300_ALERT_4_G Atom Site Occupancy of H11K       Constrained at        0.5 Check
PLAT300_ALERT_4_G Atom Site Occupancy of H11L       Constrained at        0.5 Check
PLAT300_ALERT_4_G Atom Site Occupancy of H11M       Constrained at        0.5 Check
PLAT300_ALERT_4_G Atom Site Occupancy of H96A       Constrained at        0.5 Check
PLAT300_ALERT_4_G Atom Site Occupancy of H96B       Constrained at        0.5 Check
PLAT300_ALERT_4_G Atom Site Occupancy of H96C       Constrained at        0.5 Check
PLAT300_ALERT_4_G Atom Site Occupancy of H96D       Constrained at        0.5 Check
PLAT300_ALERT_4_G Atom Site Occupancy of H97A       Constrained at        0.5 Check
PLAT300_ALERT_4_G Atom Site Occupancy of H97B       Constrained at        0.5 Check
PLAT300_ALERT_4_G Atom Site Occupancy of H98A       Constrained at        0.5 Check
PLAT300_ALERT_4_G Atom Site Occupancy of H98B       Constrained at        0.5 Check
PLAT300_ALERT_4_G Atom Site Occupancy of H99A       Constrained at        0.5 Check
PLAT300_ALERT_4_G Atom Site Occupancy of H99B       Constrained at        0.5 Check
PLAT301_ALERT_3_G Main Residue  Disorder ..............(Resd  1  )         8% Note
PLAT343_ALERT_2_G Unusual sp3      Angle Range in Main Residue for        C70 Check
PLAT343_ALERT_2_G Unusual sp3      Angle Range in Main Residue for        C72 Check
PLAT343_ALERT_2_G Unusual          Angle Range in Main Residue for        C73 Check
PLAT410_ALERT_2_G Short Intra H...H Contact  H40      ..H96C     .       2.03 Ang.
                                                   x,y,z  =      1_555 Check
PLAT411_ALERT_2_G Short Inter H...H Contact  H10T     ..H93A     .       2.10 Ang.
                                               -x,1-y,-z  =      2_565 Check
PLAT411_ALERT_2_G Short Inter H...H Contact  H11K     ..H94A     .       2.01 Ang.
                                               -x,1-y,-z  =      2_565 Check
PLAT413_ALERT_2_G Short Inter XH3 .. XHn     H10H     ..H71B     .       2.06 Ang.
                                             -1+x,y,-1+z  =      1_454 Check
PLAT413_ALERT_2_G Short Inter XH3 .. XHn     H10I     ..H71C     .       2.05 Ang.
                                             -1+x,y,-1+z  =      1_454 Check
PLAT413_ALERT_2_G Short Inter XH3 .. XHn     H10L     ..H63B     .       2.14 Ang.
                                             1-x,1-y,1-z  =      2_666 Check
PLAT413_ALERT_2_G Short Inter XH3 .. XHn     H10M     ..H79A     .       2.14 Ang.
                                             -1+x,y,-1+z  =      1_454 Check
PLAT432_ALERT_2_G Short Inter X...Y Contact  C70      ..C72      .       3.20 Ang.
                                              1-x,-y,1-z  =      2_656 Check
PLAT432_ALERT_2_G Short Inter X...Y Contact  C78      ..C79      .       3.14 Ang.
                                              2-x,-y,1-z  =      2_756 Check
PLAT432_ALERT_2_G Short Inter X...Y Contact  C79      ..C106     .       3.20 Ang.
                                               1+x,y,1+z  =      1_656 Check
PLAT720_ALERT_4_G Number of Unusual/Non-Standard Labels .........           2 Note
PLAT773_ALERT_2_G Check long C-C Bond in CIF: C98      --C112            1.87 Ang.
PLAT773_ALERT_2_G Check long C-C Bond in CIF: C99      --C112            1.85 Ang.
PLAT773_ALERT_2_G Check long C-C Bond in CIF: C99      --C113            2.03 Ang.
PLAT773_ALERT_2_G Check long C-C Bond in CIF: C99      --C115            2.01 Ang.
PLAT779_ALERT_4_G Suspect or Irrelevant (Bond) Angle(s) in CIF ...      21.80 Deg.
```

```
              H96C -C96  -H96A    1_555   1_555   1_555 ......     #  424 Check
PLAT779_ALERT_4_G Suspect or Irrelevant (Bond) Angle(s) in CIF ...      21.70 Deg.
              H96D -C96  -H96B    1_555   1_555   1_555 ......     #  427 Check
PLAT779_ALERT_4_G Suspect or Irrelevant (Bond) Angle(s) in CIF ...      21.20 Deg.
              C97  -C96  -C115    1_555   1_555   1_555 ......     #  434 Check
PLAT779_ALERT_4_G Suspect or Irrelevant (Bond) Angle(s) in CIF ...      39.40 Deg.
              C112 -C97  -H97B    1_555   1_555   1_555 ......     #  449 Check
PLAT779_ALERT_4_G Suspect or Irrelevant (Bond) Angle(s) in CIF ...      36.50 Deg.
              C115 -C97  -C98     1_555   1_555   1_555 ......     #  453 Check
PLAT779_ALERT_4_G Suspect or Irrelevant (Bond) Angle(s) in CIF ...      17.30 Deg.
              C115 -C98  -C97     1_555   1_555   1_555 ......     #  465 Check
PLAT779_ALERT_4_G Suspect or Irrelevant (Bond) Angle(s) in CIF ...      32.80 Deg.
              C98  -C99  -C115    1_555   1_555   1_555 ......     #  474 Check
PLAT779_ALERT_4_G Suspect or Irrelevant (Bond) Angle(s) in CIF ...      40.00 Deg.
              C113 -C99  -H99B    1_555   1_555   1_555 ......     #  487 Check
PLAT779_ALERT_4_G Suspect or Irrelevant (Bond) Angle(s) in CIF ...      20.20 Deg.
              C115 -C112 -C97     1_555   1_555   1_555 ......     #  579 Check
PLAT779_ALERT_4_G Suspect or Irrelevant (Bond) Angle(s) in CIF ...      36.30 Deg.
              C115 -C112 -C98     1_555   1_555   1_555 ......     #  580 Check
PLAT779_ALERT_4_G Suspect or Irrelevant (Bond) Angle(s) in CIF ...      38.30 Deg.
              C98  -C115 -H11M    1_555   1_555   1_555 ......     #  613 Check
PLAT789_ALERT_4_G Atoms with Negative _atom_site_disorder_group  #         33 Check
PLAT793_ALERT_4_G Model has Chirality at C65         (Centro SPGR)          R Verify
PLAT793_ALERT_4_G Model has Chirality at C81         (Centro SPGR)          R Verify
PLAT860_ALERT_3_G Number of Least-Squares Restraints ............         776 Note
PLAT883_ALERT_1_G No Info/Value for _atom_sites_solution_primary .     Please Do !
PLAT910_ALERT_3_G Missing # of FCF Reflection(s) Below Theta(Min).          1 Note
PLAT912_ALERT_4_G Missing # of FCF Reflections Above STh/L=  0.600        124 Note
PLAT933_ALERT_2_G Number of HKL-OMIT Records in Embedded .res File         14 Note
PLAT941_ALERT_3_G Average HKL Measurement Multiplicity ..........         2.8 Low
PLAT978_ALERT_2_G Number C-C Bonds with Positive Residual Density.          1 Info
PLAT992_ALERT_5_G Repd & Actual _reflns_number_gt Values Differ by          8 Check
```

---

0 **ALERT level A** = Most likely a serious problem - resolve or explain
1 **ALERT level B** = A potentially serious problem, consider carefully
31 **ALERT level C** = Check. Ensure it is not caused by an omission or oversight
123 **ALERT level G** = General information/check it is not something unexpected

2 ALERT type 1 CIF construction/syntax error, inconsistent or missing data
43 ALERT type 2 Indicator that the structure model may be wrong or deficient
19 ALERT type 3 Indicator that the structure quality may be low
90 ALERT type 4 Improvement, methodology, query or suggestion
1 ALERT type 5 Informative message, check

It is advisable to attempt to resolve as many as possible of the alerts in all categories. Often the minor alerts point to easily fixed oversights, errors and omissions in your CIF or refinement strategy, so attention to these fine details can be worthwhile. In order to resolve some of the more serious problems it may be necessary to carry out additional measurements or structure refinements. However, the purpose of your study may justify the reported deviations and the more serious of these should normally be commented upon in the discussion or experimental section of a paper or in the "special_details" fields of the CIF. checkCIF was carefully designed to identify outliers and unusual parameters, but every test has its limitations and alerts that are not important in a particular case may appear. Conversely, the absence of alerts does not guarantee there are no aspects of the results needing attention. It is up to the individual to critically assess their own results and, if necessary, seek expert advice.

Publication of your CIF in IUCr journals

A basic structural check has been run on your CIF. These basic checks will be run on all CIFs submitted for publication in IUCr journals (*Acta Crystallographica*, *Journal of Applied Crystallography*, *Journal of Synchrotron Radiation*); however, if you intend to submit to *Acta Crystallographica Section C* or *E* or *IUCrData*, you should make sure that full publication checks are run on the final version of your CIF prior to submission.

Publication of your CIF in other journals

Please refer to the *Notes for Authors* of the relevant journal for any special instructions relating to CIF submission.

PLATON version of 28/11/2022; check.def file version of 28/11/2022

Datablock a - ellipsoid plot

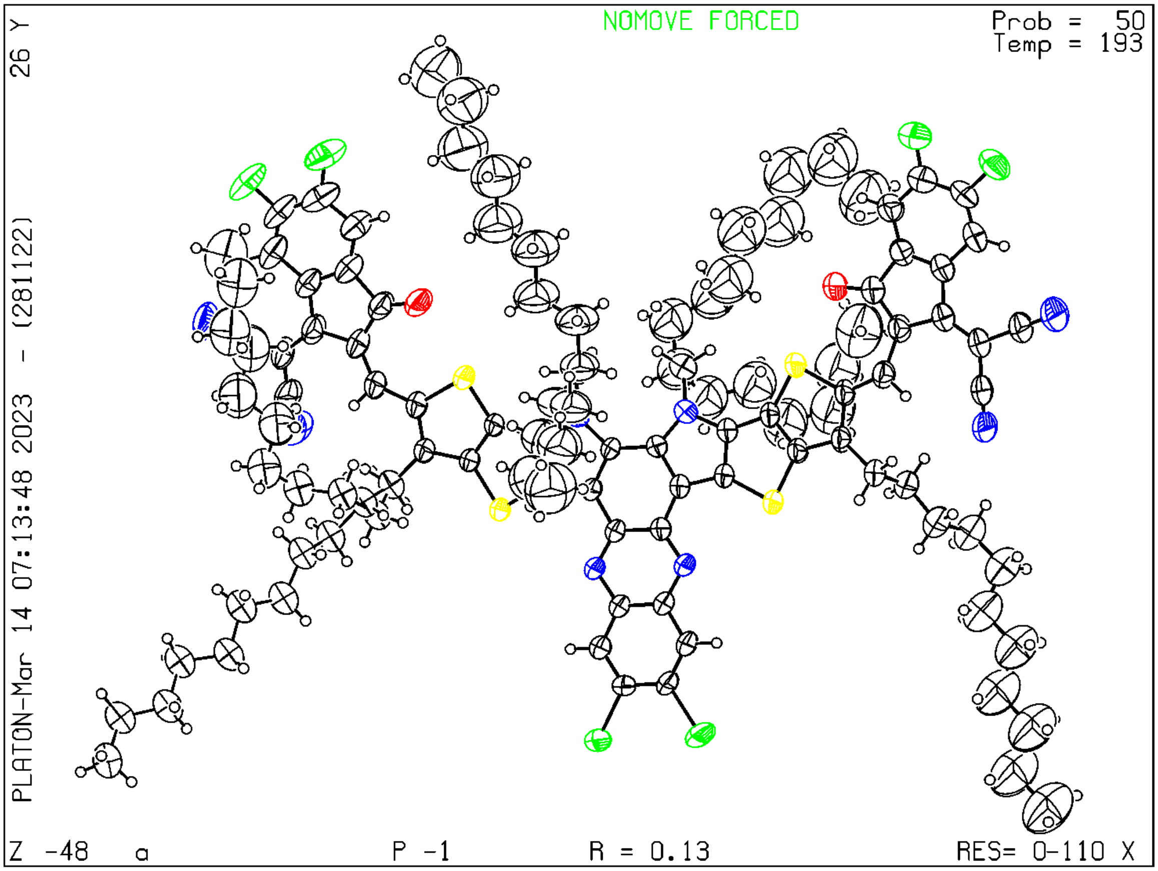

Supplement: Supplementary file 7 — Supplementary Data 4 [file 41467_2023_40423_MOESM7_ESM.pdf]
